# Data-Driven Approach for Bioreactor Monitoring and Prediction in Monoclonal Antibody Production

**DOI:** 10.1021/acsomega.6c04405

**Published:** 2026-09-08

**Authors:** Kadeejathul Kubra, Munawar A. Shaik

**Affiliations:** Department of Chemical and Petroleum Engineering, College of Engineering, UAE University, Al Ain P.O. Box 15551, UAE

## Abstract

The upstream biopharmaceutical processes of monoclonal antibody (mAb) production generate high-dimensional and irregularly sampled time-series data with many missing values, which can limit the robustness and reusability of data-driven models. In this study, a data-driven workflow has been developed for a previously published industrial fed-batch dataset for the mAb production process. The workflow provides comprehensive data analysis from data cleaning and imputation through model benchmarking and interpretability to enable soft sensing and predictive modelling of mAb bioreactors. Initially, the missing value imputation and their evaluation were carried out using eight supervised machine learning (ML) regressors with temporally engineered time-dependent features. Light gradient boosting machine (LightGBM) provided the best imputed data among the eight ML models and was used as the imputation model for all the variables. Compared with a previously published dual-hybrid imputation method, the LightGBM-based imputation pipeline preserved all observed measurements exactly. It reproduces more accurately both marginal distributions and the multivariate geometry of the data. Using this imputed dataset, thirty-four single and stacked ML models were benchmarked for two industrially relevant tasks. These were used for (i) pointwise soft sensing of the mAb titer using concurrent process variables and (ii) early-stage prediction of final mAb titer using data from 1–7 days. Nonlinear tree-based ensembles achieved test R^2^ up to 0.98 for soft sensing and 0.93 for early prediction as compared to linear and kernel-based models. However, stacked ensembles provided modest but consistent gains in accuracy and robustness over the best single tree-based ensembles. Finally, permutation variable importance, Shapley additive explanations, and partial dependence analysis showed that elapsed culture time, total cell density, culture volume, glutamine, and glutamate are the most important variables of the predicted titer, whereas tightly controlled variables such as glucose and pH contribute little within the studied operating range. This is the first study that combined modern ML-based imputation, extensive model benchmarking, and explainable artificial intelligence on an industrial mAb dataset to develop both soft sensors and early prediction models. Overall, the workflow offers a simple, reusable, and explainable framework for handling missing data and building soft sensors in industrial mAb upstream development with the potential to reduce analytical burden, speed up feedback, and support data-driven decision-making in mAb upstream processes.

## Graphical abstract

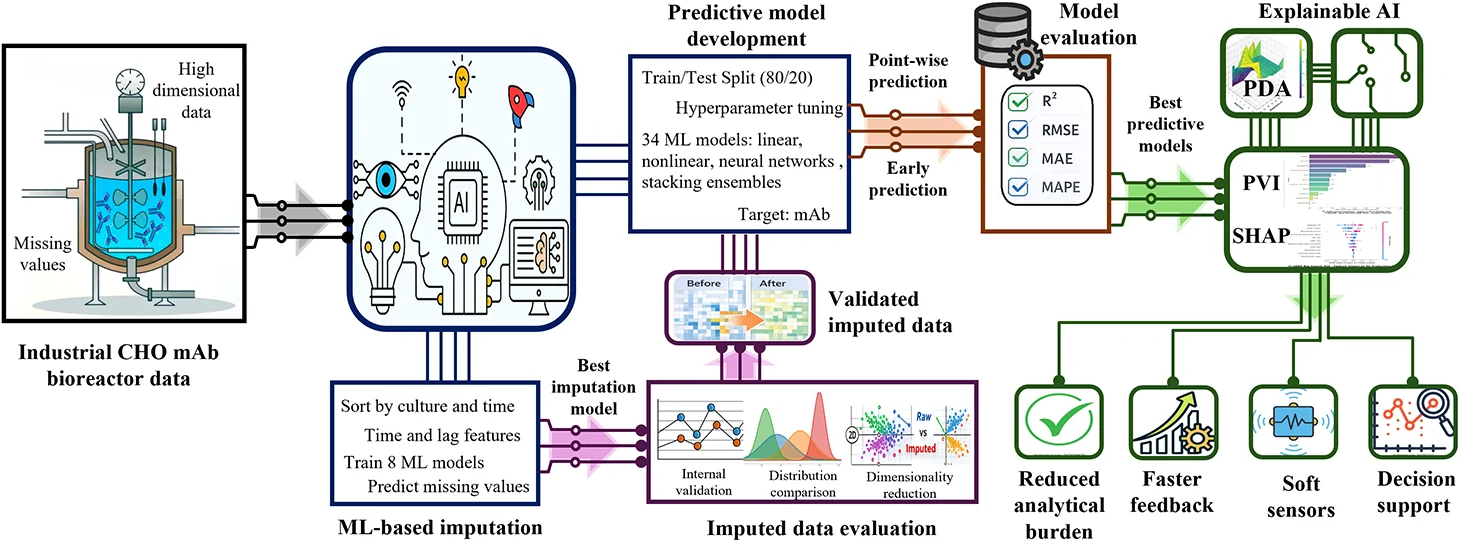

## 1 Introduction

Biopharmaceutical manufacturing of monoclonal antibodies (mAbs) has grown significantly since the first approval of Orthoclone OKT3 for the prevention of kidney transplant rejection.^1^ Since then, this class of drugs has expanded significantly. Currently, these are used to treat autoimmune and inflammatory diseases, infectious diseases, and various cancers. Because of their biologically obtained nature and specific mechanism, they do not have as many side effects compared with other therapeutics. Reflecting this success, the global mAb market value in 2025 is estimated to be approximately USD 292.7 billion and is expected to expand to approximately USD 909.0 billion by 2035.^2^ This rapid expansion puts a great burden on manufacturers to give more titers, product quality, and lower the cost of goods through optimized upstream and downstream bioprocesses.

However, biopharmaceutical processes are inherently complex. Mammalian cell culture processes are characterized by nonlinear and time-dependent dynamics, interactions among nutrients, metabolites, and environmental conditions, and close relations between process parameters and critical quality attributes (CQAs).^3,4^ The effect of culture parameters on the process performance initially started with one-factor-at-a-time (OFAT) experimentation, in which the variables of the bioreactor, including temperature, pH, dissolved oxygen, and feed rates, are varied separately.^5^ OFAT is easy to implement but is not effective in characterizing a multivariate process as univariate problems and hence does not capture interactions among variables. Apart from OFAT, design of experiments (DoE) methods have been used widely to systematically understand the effect of culture parameters on process performance.^6,7^ Even though their main goal is to reduce the number of required experiments, DoE becomes challenging when dealing with high-dimensional, time-dependent industrial datasets.^8,9^ Multivariate data analysis (MVDA) is often used to address the complexity and multidimensionality of bioprocess data.^10^ Common MVDA techniques include principal component analysis (PCA)^11^ and partial least-squares regression (PLSR)^12^ techniques for visualization and regression data analysis. Such linear chemometric methods can reduce data dimensionality and provide a systematic approach to finding correlations, importance, and outliers among variables.^13^ However, because PCA and PLSR are fundamentally linear methods and typically depend on Gaussian latent-variable assumptions,^14^ their ability to capture the nonlinear dynamics of cell culture processes is limited,^13^ and they may give misleading results when applied to highly nonlinear, high-dimensional industrial datasets.^14,15^ Mechanistic and empirical models have also been applied to correlate variables such as temperature, glucose concentration, density of the cells, and product formation rates to understand cell growth and mAb productivity and to investigate cell aggregation and glycosylation.^16,17^ Even though these models are useful in understanding cell behavior and product quality issues, they are often computationally expensive, require significant expertise in model development and parameter estimation, and may not be easily transferable from one cell line to another.^18^

The use of data-driven methods has introduced new opportunities for optimization. Artificial intelligence (AI) and machine learning (ML) are useful tools for addressing the limitations of the traditional pharmaceutical processes. They allow for better prediction, optimization, and decision-making based on data.^19^ These are particularly useful for managing high-dimensional datasets and speeding up research processes that normally take a long time.^20^ For example, statistical learning techniques such as PLSR were among the earliest tools that were used to find relationships between multivariate process data and critical quality attributes.^21,22^ Narayanan et al. examined decision tree-based PLS (DT-PLS) regression to account for nonlinearities within the fed-batch bioreactor.^23^ The findings indicated that the DT-PLS algorithm has automatically determined clusters of characteristic processes and constructed local PLS models on each cluster. This simplifies the modeling of bioprocess nonlinearities while increasing the robustness and interpretability. The results showed that DT-PLS-based regression improved CQA prediction accuracy compared to more traditional approaches. The prediction of multiple quality attributes was also conducted by combining process performance based modeling (PBM) with ML, which allowed the manufacturers to predict the product quality and quantity early and accurately from process data.^24^

Soft-sensor approaches have also been investigated for estimating cell growth, mAb production, intracellular metabolites, and glycan profiles from process measurements. For example, Liang et al. compared mechanistic Michaelis–Menten kinetic (MMK), orthogonal partial least-squares (OPLS), and neural-network (NN) models.^25^ The mechanistic model provided the most accurate predictions of cell growth and mAb production, whereas the OPLS model was more effective for predicting intracellular nucleotide-sugar donors and glycan composition.

Other studies focused on improving mAb production through metabolic and media components optimization. For example, Hernández et al. used data-driven modelling to analyze the effects of different medium compositions on cell metabolism, growth, and mAb production.^26^ Similarly, Kang et al. analyzed the effect of polyamines in cell cultures.^27^ They found that polyamine addition can enhance cell growth and mAb production. The end-to-end ML approach was used by Gangwar et al. to optimize media composition and to predict the acidic and basic charge variants in Chinese Hamster Ovary (CHO) cell cultures.^28^ Feature selection using random forest, Pearson correlation, and Shapley additive explanations (SHAP) identified Zn and Fe as important contributors to the basic and acidic charge variant profiles, respectively. This demonstrates an effective method that could enable faster and more targeted media development.

However, data quality is another issue. Gangadharan et al. reviewed metaheuristic approaches for data preprocessing analysis, highlighting that upstream datasets are multivariate time series with heterogeneous variables and complex missing-data patterns.^29^ Because of the temporal patterns and mixed missingness, they mentioned that previously available simple methods are not sufficient to capture complex trends and may introduce bias in gap filling. Building on these insights, they proposed a dual-hybrid imputation method and applied it to an upstream fed-batch dataset of mAb production to predict performance using the sixth day of culture data.^30^ The dual-hybrid methodology, composed of a genetic algorithm (GA), fuzzy C-means clustering (FCM), and support vector regression (SVR) was used to deal with time-dependent parameters, and Stineman interpolation was used to handle time-independent parameters. The authors stated that they were able to predict the performance of the harvest with only four process parameters halfway through the operation of the process. Moreover, they demonstrated that the proposed hybrid methodology satisfies certain incompatible statistical mechanisms, therefore positively influencing the analysis of bioprocess data of different structures and avoiding technical errors.

A review further emphasized that missing data is one of the most common practical problems in mAb process development.^31^ A variety of methods have been used to handle missing data, ranging from modified time-series imputation algorithms to hybrid schemes based on clustering, SVR, and genetic algorithms to simple heuristic approaches like mean imputation and polynomial regression for time-series data.^29,31^ In a study of the historical biologics manufacturing databases, mean imputation and multivariate regression were analyzed as easy alternatives for filling missing values.^32^ Based on a comparison, they reported that mean substitution is an easy and efficient approach compared to smooth nondynamical datasets, while regression-based imputation better preserves the standard deviation and distributional shape in dynamical datasets with less than 30% missing values. They also emphasized that the missing data mechanism is a key factor in selecting an appropriate imputation strategy.

Despite the major progress that has been made in modeling and data-driven process development for mAb production, several practical challenges remain in applying ML to upstream bioprocesses. In practice, industrial and often historical cell culture datasets are incomplete and irregularly sampled with missing values and high dimensionality.^33^ These issues can reduce the reproducibility and robustness of the ML models. Although Gangadharan et al. proposed a dual-hybrid framework for the imputation of missing data and prediction of the performance of mAb,^30^ their method requires manual grouping of the variables and multiple software environments, which increases complexity and limits reusability.

Previous studies have addressed different components of the upstream data-analysis workflow, including imputation, soft sensing, final titer prediction, media optimization, model comparison, or explainable feature analysis. Recent literature also identifies missing and irregularly sampled data, limited benchmark datasets, model interpretability, and transferability as major barriers to reliable ML implementation in bioprocessing.^33^ However, based on the literature reviewed, no previous study was identified that jointly evaluated imputation quality, broad single and stacked model benchmarking, complementary soft-sensing and early final-titer prediction tasks, and explainable AI analysis using the same industrial upstream dataset.

Motivated by these challenges, this study develops an integrated Python-based ML workflow for the industrial CHO mAb dataset, which was originally published by Gangadharan et al.^30^ The academic contribution and novelty of this work lie in four main aspects: (i) we develop a simple ML-based imputation pipeline for irregularly sampled upstream mAb process data without manual variable manipulation and compare its performance with the previously published dual-hybrid imputation method on the same dataset. (ii) Imputation quality is quantitatively evaluated not only using error metrics but also through observed value preservation, distribution similarity, and dimensionality-reduction-based visualization of multivariate data geometry. (iii) 34 single and stacked ML models are systematically benchmarked for two complementary prediction tasks: soft sensing of mAb concentration and early prediction of the final harvest concentration. (iv) The final predictive models are interpreted by using a set of explainable artificial intelligence (XAI) techniques to identify key process variables and their relationships with mAb production. Therefore, although the dataset originates from an industrial case, the main contribution of this study is a quantitative, reusable, explainable ML workflow for missing data handling, soft sensing, early titer prediction, and biological interpretation in upstream mAb process development.

## 2 Methodology

This section discusses the data-driven workflow that was developed in this study. The overview of the main steps from raw data to model interpretation is shown in Figure 1.

**Figure 1:**
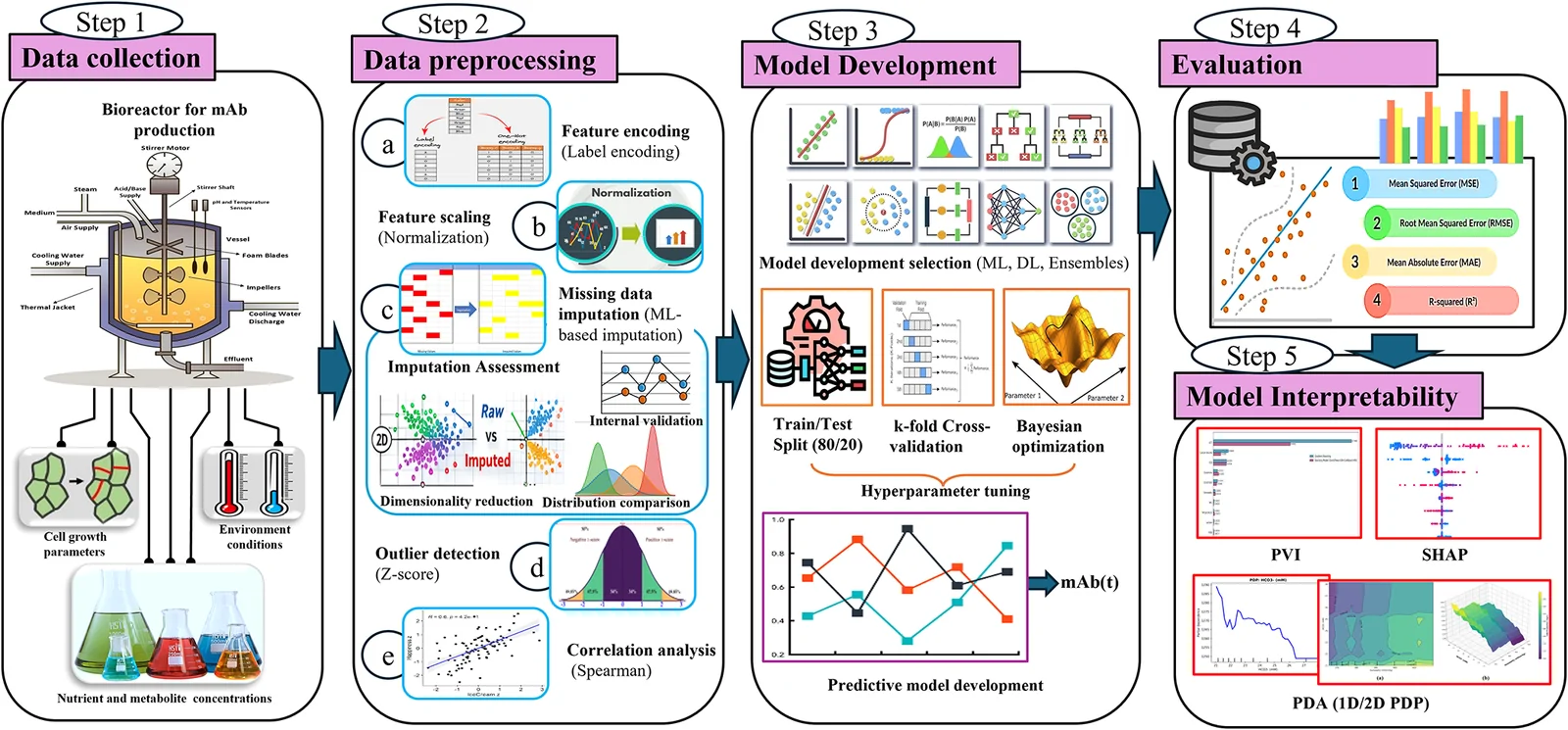
Overview of data-driven workflow for imputation and prediction in mAb bioreactor.

### 2.1 Dataset Description

In this study, the dataset is taken from the work by Gangadharan et al.,^30^ which published upstream bioprocess data from AstraZeneca’s process development and manufacturing records. The data is based on mammalian cell culture and the use of CHO cells to produce various mAb products. They shared normalized raw data to protect confidential information. The complete data consists of 106 independent bioreactor cultures with 23 process variables, which were collected over a seven-year period (2010–2016). Every culture is followed for 17 days, creating a multivariate time-series dataset. The parameters of upstream culture over time include cell growth parameters (viable cell density (VCD), total cell density (TCD), average cell volume (ACV), elapsed generation number (EGN), average cell compactness (ACC)), culture environment parameters (temperature, pH, pCO_2_ expressed as percentage CO_2_, pO_2_ expressed as percentage air saturation, osmolality), nutrient and metabolite concentrations (glutamine, glutamate, glucose, lactate, ammonia (NH_3_), sodium (Na^+^), potassium (K^+^), bicarbonate (HCO_3_^–^)), and mAb titer. Before moving to data preprocessing, the normalized data are converted back to their actual values. For this purpose, the range of each process parameter in the bioreactor for the mAb production is obtained from the literature.^22,34–40^ The pO_2_ variable was expressed as percentage air saturation, using a literature-based range of 10–40%, whereas pCO_2_ was expressed as percentage CO_2_, using a range of 5–20%. At approximately atmospheric pressure, 5–20% CO_2_ corresponds to a pCO_2_ range of approximately 38–150 mmHg.

### 2.2 Data Preprocessing

The data preprocessing step is to produce clean, structured data that are suitable for model development.^41,42^ This step involves feature encoding, feature scaling, missing data handling and imputation, outlier detection, and analysis of the correlation between the variables.^42,43^ Initially, the categorical variables are encoded into an integer representation (Culture_ID_Code) using label encoding so that it can be used as an input feature for the learning algorithm. Then, rows containing zero or biologically impossible values were manually removed.

Feature scaling transforms numerical features to a comparable scale so that no single feature dominates the learning process because of its magnitude. Numerical features are standardized using the scikit-learn StandardScaler, which centers each feature to a mean of zero and scales it to unit variance. This procedure prevents variables with larger numerical ranges from disproportionately influencing the scale-sensitive learning algorithms. The procedures for missing data handling and imputation, outlier detection, and correlation analysis are presented in the following subsections.

#### 2.2.1 Missing Data Imputation Using ML Models

To address the missing data in the upstream process variables, an ML-based imputation is adopted. For this purpose, the cleaned raw data are sorted on bioreactor time to keep the time sequence of measurements within each culture. To better capture the temporal dynamics of the bioreactor cultures, we generated engineered time-dependent features. For each culture, transformed bioreactor time includes culture-specific time index (culture_time_idx) together with nonlinear transformations like squared (time_squared) and logarithmic time (time_log). Further, to study the temporal structure, lagged features (feature_lag_1 to feature_lag_5) are generated for each numerical process variable within each culture. These features are used to enhance the ability of the model to learn nonlinear and time-dependent relationships in upstream bioprocess data. Eight supervised ML models are implemented for missing-value imputation using Python, either through scikit-learn or through the scikit-learn-compatible interface provided by the corresponding ML libraries.

For each variable with missing values, the ML models with specified parameters are trained on the rows in which that variable is experimentally observed. All of the other available process variables, time-related features, and lag features are used as inputs to the model. The imputation models were evaluated using an internal masking-validation procedure. For each variable, approximately 10% of the experimentally observed values are temporarily masked in continuous time blocks within each culture and used as an internal validation set. The remaining observed values were used to train the imputation model. The model then predicted the masked values. For validation, coefficient of determination (*R* ^2^) and root-mean-square error (RMSE) are calculated by comparing these predictions with the corresponding original measurements. After validation, the models are used to predict only the originally missing values of each variable without any change in all original (nonmissing) measurements. This step is repeated iteratively for all missing variables, resulting in a complete imputed dataset to produce mAb in the bioreactor. The final imputed dataset contains both the originally measured values and the corresponding gap-filled values for all variables, including mAb titer. This complete structured dataset is used for outlier-culture detection before subsequent predictive modeling.

Because the true experimental values at the originally missing positions are unavailable, the accuracy of the final gap-filled values cannot be evaluated directly. Therefore, the imputation quality is assessed using multiple complementary approaches. First, the raw and imputed datasets are compared at positions where observations are available in the original dataset. *R* ^2^ and RMSE were calculated at these observed positions to assess whether the imputation procedure preserves the original measurements without alteration. In addition, distribution-based metrics and dimensionality-reduction analyses are used to examine whether the statistical distributions and overall multivariate structure of the dataset are preserved after imputation.

The empirical distributions of each variable in the raw and imputed datasets are analyzed using error- and distance-based metrics. For each process variable, the raw and imputed values are compared at the observed positions and the RMSE and *R* ^2^ are calculated. Further, three commonly used measures are evaluated, namely, Wasserstein distance,^44^ Jensen-Shannon (JS) divergence,^45^ and Kolmogorov-Smirnov (KS) statistic^46^ with associated p-value, for comparing probability distributions. These measures have their own characteristics and strengths and can be useful in the fields of ML, goodness-of-fit testing, and nonparametric statistics. Using them together can give a more comprehensive picture of the similarity between the two distributions.

Finally, to analyze the global structure of the data, we apply dimensionality reduction techniques. These methods include principal component analysis,^47,48^ t-distributed stochastic neighbor embedding (t-SNE),^49^ and uniform manifold approximation and projection (UMAP).^50^ These methods provide a visualization of the higher-dimensional data on a lower-dimensional space and allow a comparative analysis of the entire geometry of data before and after imputation. Raw and imputed data are projected onto two-dimensional spaces and examined to analyze whether the overall geometric structures and clustering patterns are preserved after imputation. The raw and imputed datasets are provided as Supporting Information Excel files.

#### 2.2.2 Outlier Culture Detection

The presence of outliers and extreme values in datasets may have strong impacts on the overall performance of the model.^51^ Thus, after imputation, abnormal cultures are identified from the globally standardized and imputed dataset using the global *Z*-score method. To detect outliers, culture-wise means (μ_culture_) of each variable are computed. The global mean (μ_global_) and global standard deviation (σ_global_) of these culture-wise means are then computed across cultures. A *Z*-score for each culture and variable is then calculated as

1 $$Z=\frac{\mu_{\mathrm{culture}}-\mu_{\mathrm{global}}}{\sigma_{\mathrm{global}}}$$

A threshold of |*Z*| > 3.5 is chosen to flag extreme deviations and to avoid many false outliers. If a culture has |*Z*| > 3.5 for a given variable, it is flagged as an outlier for that variable and marked with a binary indicator (is_outlier_culture). Culture-level metrics are further calculated using summary metrics from the globally scaled data. These include mean value, mean absolute value, variance, and maximum absolute *Z*-score for all variables. These are used to rank and interpret cultures that have abnormal multivariate profiles.

#### 2.2.3 Exploratory Correlation Analysis

Exploratory feature analysis is conducted to investigate relationships between process variables and output variables.^52^ mAb titer is the output variable, and explanatory variables include cell growth parameters, nutrient and metabolite concentrations, and culture environment measurements. Monotonic associations between the process variables and mAb are calculated using Spearman rank correlation. The Spearman rank coefficient (ρ*_j_*) for each variable *j* is calculated as

2 $$\rho_{j}=\frac{\sum_{i=1}^{n}{(x}_{j,i}-\overset{®}{x_{j}\;})\left(y_{i}-\mathrm{y̅}\right)}{\sqrt{\sum_{i=1}^{n}{{(x}_{j,i}-\overset{®}{x_{j}\;})}^{2}\sum_{i=1}^{n}{{(y}_{i}-\mathrm{y̅})}^{2}}},\;\left(j=1,2,3,..\right)$$

where, *x*_*j,i*_ and *y_i_* are the rank-transformed values of variable *j* and target mAb for sample i (average ranks for ties), *x̅_j_* and *y̅* are their mean ranks, and *n* is the number of samples. The coefficient is bounded over [−1, 1].^53,54^

### 2.3 Predictive ML Model Development

Following completion of the imputation and preprocessing stage, a separate predictive-modeling analysis is conducted using the completed dataset. Supervised ML models are developed to predict the mAb titer under two different scenarios. In scenario 1, pointwise mAb prediction is performed, where the mAb concentration at each time point is predicted based on the process conditions measured at that same time point. In scenario 2, early-stage prediction of final mAb titer is carried out, where data from 1 to 7 days are used as inputs with a target of final harvest day mAb titer for each culture.

A set of 34 benchmark models from machine learning, neural networks, and stacking ensemble approaches was analyzed. These comprise random forest (RF), extremely randomized trees (ExtraTrees), gradient boosting regressor (GBR), extreme gradient boosting (XGBoost), categorical boosting (CatBoost), light gradient boosting (LightGBM), k-nearest neighbors (KNN), decision tree, support vector regression, linear regression (LR), ridge regression (RR), lasso regression (LASSO), elastic net (EN) regression, and a multilayer perceptron (MLP) with a single hidden layer.

Beyond these single models, stacked ensemble models are also constructed by combining different base learners.^55,56^ Stacking is an ensemble technique in which several base models are trained, and their predictions are then combined by a meta-learner. Hence, stacking can improve predictive accuracy by combining the strengths of different algorithms and reducing model-specific bias and variance. The stacked ensembles use various combinations of bagging methods like RF and ExtraTrees, boosting methods include GBR, XGBoost, CatBoost, and LightGBM, as well as nontree-based models such as SVR and MLP, which provide architectural diversity. In these stacks, regularized linear models, primarily RR, and in some cases LR and XGBoost are used as metalearners. The number and type of base learners are varied systematically for the development of different stacked models. In the simplest case, two base learners are stacked (e.g., RF + GBR, RF + XGBoost, and RF + CatBoost) with RR or LR as the meta-learner. More complex stacks use three or more base learners (e.g., ExtraTrees + GBR + LightGBM, RF + GBR + SVR + MLP). Moreover, all-boosting stacks are also considered, where GBR, XGBoost, CatBoost, and LightGBM, sometimes with MLP and SVR, are used as the base learners.

#### 2.3.1 Training Configuration

For model development and evaluation, the complete imputed dataset is randomly split into separate training and test sets. The split can be chosen on the basis of the size of the data. However, an 80/20 split is commonly used in practice and provides a good balance between model fitting and independent evaluation. In this study, the data are divided into a training set and a test set, in the ratio of 80 and 20%. To ensure the reproducibility of the analysis, a fixed random seed is used (random_state = 42). Therefore, both originally measured and imputed values were included in the training and test sets.

To identify the best-suitable hyperparameters for the ML models, hyperparameter tuning is carried out so that model performance and generalizability can be improved, and bias and variance can be balanced.^57^ Hyperparameters are tuned on the training set using Bayesian optimization through the scikit-optimize BayesSearchCV interface, where 5-fold cross-validation and the coefficient of determination are used as objectives for the hyperparameter selection. Bayesian optimization was performed for 50 iterations for each model. The test set is used only for the evaluation of the final, unbiased performance to avoid data leakage.

#### 2.3.2 Evaluation of ML Models

The performance of different ML models is evaluated using four standard regression metrics to find the best models for mAb prediction. These include coefficient of determination, root mean squared error, mean absolute error (MAE), and mean absolute percentage error (MAPE). Here *y_i_* and *ŷ_i_* denote true and predicted targets, *y̅* is the split-wise mean of target (*y*), and n is the number of samples. These metrics are given as follows:

3 $$R^{2}=1-\frac{\sum_{i}{\left(y_{i}-{ŷ}_{i}\right)}^{2}}{\sum_{i}{\left(y_{i}-\mathrm{y̅}\right)}^{2}}$$

4 $$\mathrm{RMSE}=\sqrt{\frac{1}{n}\sum_{i}{\left(y_{i}-{ŷ}_{i}\right)}^{2}}$$

5 $$\mathrm{MAE}=\frac{1}{n}\sum_{i}\left|y_{i}-{ŷ}_{i}\right|$$

6 $$\mathrm{MAPE}=\frac{100}{n}\sum_{i}\left|\frac{y_{i}-{ŷ}_{i}}{y_{i}}\right|$$

### 2.4 Methods for Interpretability and Sensitivity Analysis

To understand the relationships between process variables and mAb titer and to interpret the predictions of the trained models, explainable artificial intelligence (XAI) techniques are applied after the model prediction. The model interpretability and sensitivity analyses include permutation variable importance (PVI), Shapley additive explanations (SHAP), and partial dependence analysis (PDA).

#### 2.4.1 Permutation Variable Importance

One of the general methods for quantifying the effect of each input variable on the predictive performance of the ML model is the PVI. The concept behind this is to compare the performance of the model on the original data with its performance after deliberately breaking the relationship between one variable and the target.^58,59^ In this case, the analysis is conducted in the independent test set to be consistent with the reported test performance.

The performance of the trained model (best_model) is calculated on the test set by using *R* ^2^. Then, the values of each input variable *j* are randomly permuted across the test set while all other variables are kept unchanged. The performance of the model on this permuted dataset is then recalculated. This breaks the relationship between variable *j* and the target, while maintaining the marginal distribution of the rest of the variables. The original test set is denoted as *D*_test_, the test set in which feature *j* has been selected at random permutation repeat *k* as *D*_test_^(*j,k*)^, the *R* ^2^ of the model on dataset *D* as *R* ^2^(*D*), the number of permutations as *K*. Then, the permutation importance of feature *j* is defined as

7 $${\mathrm{PVI}}_{j}=\frac{1}{K}\sum_{k=1}^{K}\left[R^{2}\left(D_{\mathrm{test}}\right)-R^{2}\left(D_{\mathrm{test}}^{\left(j,k\right)}\right)\right]$$

A larger value of PVI*_j_* means the feature *j* has a greater effect in reducing *R* ^2^, and therefore, that feature is more important.

#### 2.4.2 Shapley Additive Explanations

Further, the predictions of the trained models are interpreted using SHAP, used to measure the contribution of each input variable to individual predictions. SHAP is inductive and relies on cooperative game theory; it describes the model output as a cumulative sum of feature contributions towards every sample.^59^ In the case of a given sample *x_i_*, the model prediction *f*(*x_i_*) can be written as

8 $$f\left(x_{i}\right)=\phi_{0}+\sum_{j=1}^{p}\phi_{\mathrm{ij}}$$

where *p* is the number of input features, ϕ_0_ is a baseline prediction, i.e., the mean model prediction on the training data, and ϕ*_ij_* is the SHAP value representing the contribution of feature *j* to the prediction for sample *i*. A positive ϕ*_ij_* means feature *j* increases the prediction of mAb titer (target) compared to the baseline but a negative ϕ*_ij_* means it reduces the prediction. The feature importance is obtained globally by summing up the absolute SHAPs over all the samples. For feature *j*, the global importance *I_j_* is computed as

9 $$I_{j}=\frac{1}{N}\sum_{i=1}^{N}\left|\phi_{\mathrm{ij}}\right|$$

where *N* is the number of samples used in the analysis. A large value of *I_j_* indicates that the variables have a stronger overall influence on the model output.

#### 2.4.3 Partial Dependence Analysis

PDA examines the impact of independent variables on the dependent variables. It is a model-agnostic method for studying the marginal impact of a single variable (one-dimensional partial dependence) or two variables (two-dimensional partial dependence) on the model output when averaged over the empirical distribution of the other inputs.^59^

Suppose *f*(*x*) is the trained prediction model with an input vector *x* = (*x*_1_,···,*x*_p_). Let *x*_S_ denote a subset of features of interest (one or two process variables), with all remaining features denoted as *x*_C_ (here, S∪C = {1,···,*p*}). *x*_C,i_ denotes the observed values of the complementary features for sample *i*. Using a training dataset with m samples, the one-dimensional partial dependence function of the S feature set is defined as

10 $${\mathrm{f̂}}_{S}\left(x_{S}\right)=\frac{1}{m}\sum_{i=1}^{m}f(x_{S},x_{C,i})$$

In practice, a grid of values of *x*_S_ is selected. For each grid point, the feature of interest is fixed at that value, then the other features are kept at their observed values in the training data. Further, the model predictions are computed and the results are averaged. This gives a smooth curve known as the one-dimensional partial dependence plot (1D-PDP), that relates the marginal effect of that variable on the predicted mAb titer. Similarly, the two-dimensional partial dependence surface (2D-PDP) of two features of interest (*x*_S_1__ and *x*_S_2__) is given by

11 $${\mathrm{f̂}}_{S_{1},S_{2}}\left(x_{S_{1}},x_{S_{2}}\right)=\frac{1}{m}\sum_{i=1}^{m}f(x_{S_{1}},x_{S_{2}},x_{C,i})$$

When evaluated on a grid of (*x*_S_1__|*x*_S_2__) values, it produces a response surface (2*D*/3D PDP) that can help to identify the nonlinearities, interaction effects, plateaus, saturation, and threshold behaviors in the model response.

## 3 Results and Discussion

### 3.1 Data Analysis

The original bioreactor dataset contained substantial and structured missingness. Of the 1609 observations, 469 mAb values were missing, corresponding to 29.15%. Several process variables also contained a considerable amount of missing data. Among the 1140 observations with experimentally measured mAb values, only 138 had complete measurements of all process variables. Therefore, 1002 measured mAb observations, or approximately 87.9%, contained at least one missing process variable. Furthermore, the 138 fully complete observations represented only 38 of the 106 cultures. Restricting the prediction analysis to these complete observations would remove most of the available data and exclude a large proportion of the cultures, potentially resulting in a small and unrepresentative dataset.

The pattern of missingness in the upstream bioprocess dataset is shown in Figure 2. The heatmap shows that missing values are not scattered randomly but rather occur in blocks of certain variables in specific periods of time. This structured and time-dependent missing pattern suggests that the data are unlikely to be missing completely at random. Many recent studies have emphasized that supervised ML imputation methods can explicitly exploit intervariable correlations, temporal dependencies, and lagged dynamics when missingness involves nonlinear relationships.^60,61^ Hence, there is a need for the use of supervised ML models that utilize correlations between variables and temporal features (time indices and lagged values) for imputation.

**Figure 2:**
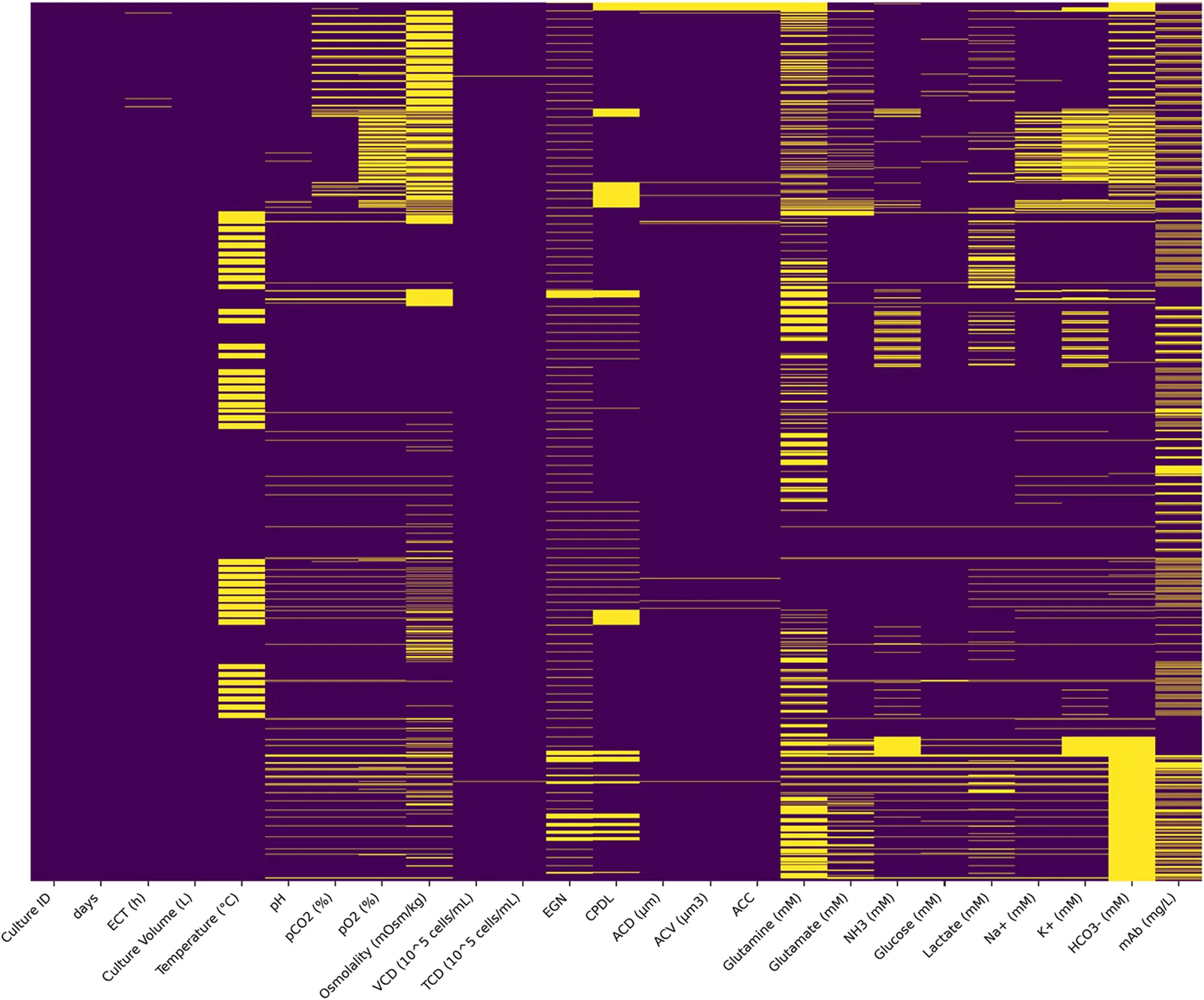
Heatmap of missing data patterns for upstream bioprocess dataset.

To analyze missingness of each variable, Table 1 summarizes the percentage of missing observations across 106 bioreactor cultures over the culture period. The percentage of missingness varies from 34.37 to 0.12%, with the largest portion of missing values was observed for glutamine, followed by HCO_3_^–^ and mAb titer. Other variables with significant missingness included K^+^, pO_2_, CPDL, and EGN, whereas VCD, TCD, ECT, and ACC had very few missing values.

**Table 1:** Summary of the Missing Observations in Percentage for Each Upstream Process Variable

variables	missing percentage (%)
glutamine (mM)	34.37
HCO_3_^–^ (mM)	29.21
mAb (mg/L)	29.15
osmolality (mOsm/kg)	28.15
temperature (°C)	24.55
K^+^ (mM)	15.85
pO_2_ (% air saturation)	13.49
CPDL	11.56
EGN	10.44
lactate (mM)	10.25
NH_3_ (mM)	8.76
Na^+^ (mM)	8.51
glutamate (mM)	8.02
pCO_2_ (% CO_2_)	7.40
pH	4.04
glucose (mM)	2.92
ACD (μm)	1.55
ACV (μm^3^)	1.55
ACC	1.55
ECT (h)	0.19
VCD (10^5^ cells/mL)	0.12
TCD (10^5^ cells/mL)	0.12

#### 3.1.1 ML-Based Imputation of Missing Data

For missing value imputation, several approaches were considered, including classical ML models, previously published methods, biologically constrained hybrid methods, and deep learning (DL) methods. However, DL models, which are commonly used for time series data,^62^ did not perform well for imputation. Because the data contain irregular and blockwise missing values, many time series are short and broken up by large gaps. As a result, the dataset does not provide enough continuous information to train these complex models but instead overfits and results in poor imputation and prediction performance. This is consistent with Chen et al., who reported that DL-based imputation performs poorly when many values are missing, as the nonlinear relationships are more difficult to learn, resulting in overfit models.^63^ Biologically constrained hybrid models are conceptually attractive, but they were also prone to overfitting and needed separate constraints for each variable and each culture,^64,65^ making them difficult to design and tune. In addition, most previously published methods require separate pipelines for time-independent and time-dependent variables and manual splitting of the data, which considerably increases the methodological complexity.^30,66^ Among these options, simple ML models were found to produce competitive results with less time and complexity (no time-based splitting). Therefore, the subsequent analysis focuses on the eight supervised ML regression models: RF, ExtraTrees, XGBoost, GBR, CatBoost, LightGBM, DT, and SVR.

The eight ML models, using their specified parameters, were evaluated through the internal masking-validation procedure described in Section 2.2.1. The predefined hyperparameter settings used for each model are provided in Table S1 (Supporting information). For each process variable, the validation performance of eight models was compared using error metrics *R* ^2^ and RMSE. Both metrics showed good performance; however, only training and validation *R* ^2^ values are summarized in Table S2 for all models. These results show that LightGBM achieved the highest overall imputation quality. A few variables showed marginally higher *R* ^2^ values for other models, but the differences were small. Therefore, LightGBM was chosen as the primary model for the final imputation pipeline. LightGBM is known to be computationally efficient and to scale well to high-dimensional tabular data,^53,67,68^ which makes it particularly suitable for this application.

To further evaluate the quality of imputed data, the fully imputed dataset obtained through LightGBM was compared with the original raw dataset using standard error metrics (RMSE, *R* ^2^), distribution-based metrics (Wasserstein distance, JS divergence, KS statistic, and KS *p*-value), and dimensionality-reduction analyses. A similar comparison was made between the raw dataset and imputed dataset reported by Gangadharan et al.,^30^ as presented in Table S3.

In Table S2, the error metrics (*R* ^2^ and RMSE) were calculated for the raw dataset and the corresponding values in the imputed datasets at positions where observations were available in the original dataset. The LightGBM-based algorithm used in this study does not alter any observed values from the original data, and hence, *R* ^2^ = 1 and RMSE = 0 for all variables. However, the dataset produced using the method of Gangadharan et al.^30^ showed nonzero RMSE and *R* ^2^ less than 1 for many variables, indicating that some of the observed values are distorted by the given approaches. For example, the previous method gave lower *R* ^2^ values for ACD, ACV, EGN, TCD, and VCD and a highly negative *R* ^2^ value for pH. The pH variable showed the largest discrepancy, with *R* ^2^ = −1272.14 and RMSE = 4.7218 for the Gangadharan et al. imputed dataset,^30^ whereas the proposed LightGBM method preserved the observed pH values exactly with *R* ^2^ = 1.000 and RMSE = 0. A closer pointwise comparison between the released raw and imputed datasets of Gangadharan et al.^30^ showed that their method not only fills in missing values but also modifies original observed values, especially for EGN, pH, and mAb titer, even though these variables were imputed separately with EGN and mAb using Stineman interpolation with SVR and pH using FCM-SVR-GA.

The distributional metrics measure how well the overall distributions of the variables are maintained after the imputation. Wasserstein distance measures the minimum effort needed to transform one probability distribution into another,^44^ JS divergence measures the difference between two probability distributions,^45^ and the KS test examines whether two samples come from the same distribution.^46^ In most variables, LightGBM-based imputed data showed lower distributional differences compared with Gangadharan et al. method.^30^ For example, imputed pH from LightGBM-based imputation produced only very small Wasserstein distance and JS divergence, a low KS statistic, and a high KS *p*-value, whereas Gangadharan et al.^30^ imputed pH showed a very large Wasserstein distance and JS divergence, a KS statistic nearer to 1, and a *p*-value of 0, suggesting that the underlying distribution has been significantly distorted.

Further, the multivariate structure of the imputed data was assessed by using three dimensionality reduction methods (PCA, t-SNE, and UMAP). PCA captures the overall linear structure of the data,^48^ t-SNE and UMAP are more focused on the local neighborhoods and clusters.^49,50^ Figure 3 shows an overlay plot between the raw and LightGBM-based imputed datasets in these low-dimensional spaces. It is obvious that the two-point set is completely overlapping, which indicates that the overall geometric structure and clustering patterns are well-preserved after imputation. In contrast, the overlay plot of raw and imputed data of Gangadharan et al.^30^ presented in Figure 4, shows a clear separation between original and imputed points in all three embeddings, which illustrates that there is distortion in the data structure.

**Figure 3:**
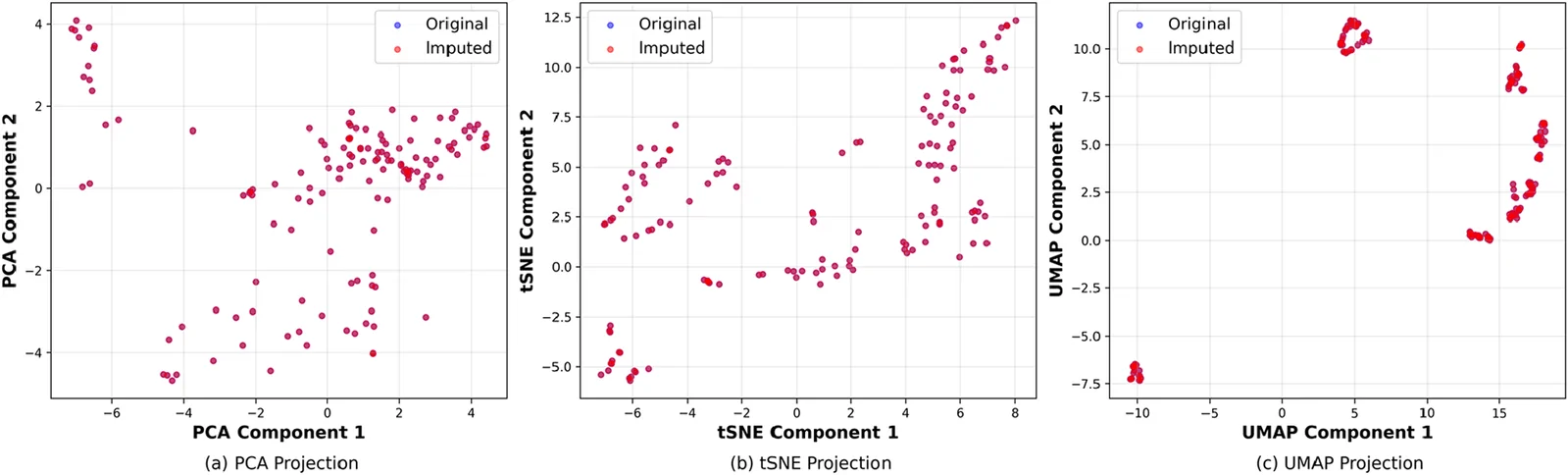
Multivariate structure of the LightGBM-imputed dataset analyzed through dimensionality reduction: (a) PCA, (b) t-SNE, and (c) UMAP projections.

**Figure 4:**
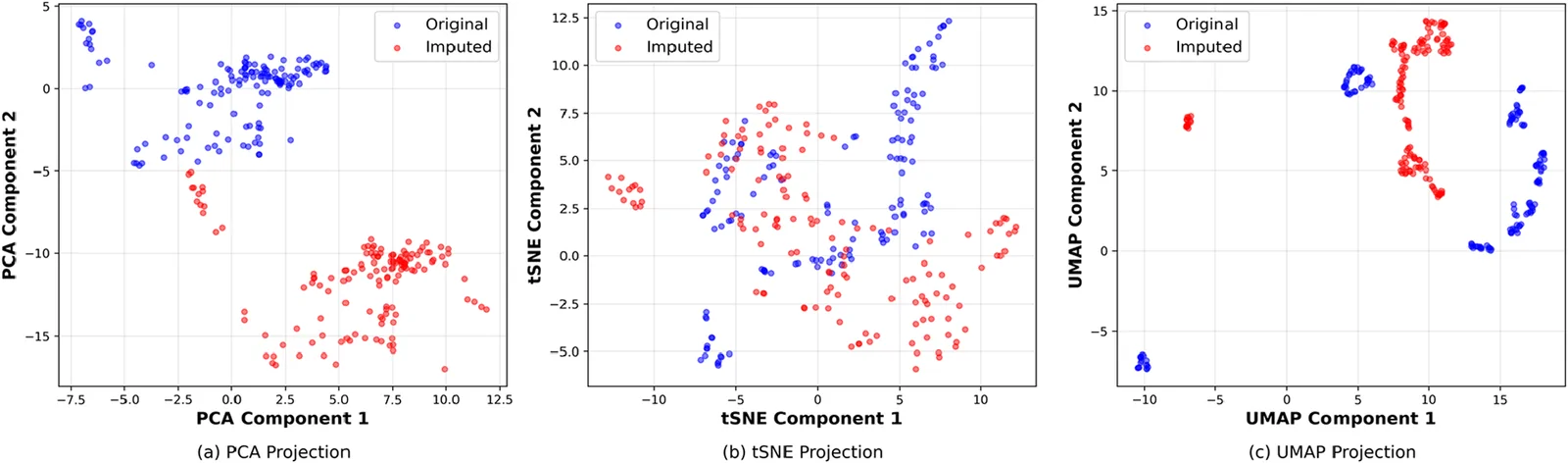
Multivariate structure reported by Gangadharan et al.,^30^ imputed dataset analyzed using dimensionality reduction: (a) PCA, (b) t-SNE, and (c) UMAP projections.

Overall, the LightGBM-based imputation pipeline is a simple and robust alternative to previously published hybrid approaches for this upstream bioprocess dataset. The algorithm by design preserves all observed measurements without modifications and produces imputations that maintain both the marginal distributions and the multivariate geometric structure of the data. Furthermore, unlike the dual-hybrid method of Gangadharan et al.,^30^ which requires separate pipelines for time-independent (FCM-SVR-GA) and time-dependent (Stineman interpolation-SVR) parameters, our approach encodes temporal information directly through time indices, lagged features, and uses a single ML framework to all variables. This avoids separate manual splitting of the data into time-dependent and time-independent blocks, reduces methodological complexity and tuning effort, and still yields an imputed dataset that is highly similar to the original data. Because the same imputation procedure can be applied to all process variables using the common set of temporal and lag-based features, the workflow is more reusable than hybrid methods that require separate model designs and tunings for different variable types. Therefore, the proposed imputation method provides a simple and transferable preprocessing step for similar irregularly sampled upstream bioprocess datasets.

#### 3.1.2 Culture-Level Outlier Analysis

Following imputation, culture-level outliers are identified using the global *Z*-score method described in Section 2.2.2. Based on the global *Z*-score criterion (|*Z*| > 3.5 on culture-wise means), nine outliers were found across eight cultures: C1, C26, C50, C96, C97, C100, C105, and C106. The flagged variable-culture pairs were ECT for C50, culture volume for C1, pO_2_ for C105, osmolality for C26, glutamate for C1, NH_3_ for C97, glucose for C96, and lactate for C100 and C106. Culture C1 was an outlier in two variables (culture volume and glutamate), while the other cultures were outliers in a single variable. Culture-level metrics (mean, mean absolute value, variance, and maximum absolute *Z*-score) further confirmed that outlier cultures had larger deviations and more extreme values than non-outlier cultures. The flagged cultures were removed before the train-test split and were therefore not included in the subsequent analyses or predictive modelling.

#### 3.1.3 Correlation Analysis

After imputation and removal of the identified outlier cultures, Spearman correlation analysis is used to examine the relationships between the process variables and mAb titer. The results are presented as a heatmap (Figure 5) and a ranked bar plot (Figure 6). Figure 5 shows that the strongest and positive relationships were between mAb titer and elapsed culture time (ECT; ρ ≈ 0.92). This suggests that longer batch times are consistently linked with the higher titers in production. Previous studies support this observation that high-titer production processes typically take 14–20 days of culture time.^69^ Several other variables also have strong positive correlations with mAb, which include glutamine concentration (ρ ≈ 0.77), EGN (ρ ≈ 0.76), osmolality (ρ ≈ 0.60), K^+^ (ρ ≈ 0.57), the cell growth parameters ACV and ACD (ρ ≈ 0.56), and total cell density TCD (ρ ≈ 0.54). These findings agree with previous studies that glutamine availability,^70,71^ osmolality regulation,^34,72^ mineral ion balance,^70^ and cell growth physiology^73,74^ are closely linked to mAb production. Especially, glutamine depletion can reduce growth and productivity,^70^ whereas appropriate supplementation, controlled osmolality, and feeding or perfusion strategies that maintain high viable cell densities have been shown to improve final titer.^34^ Moderate positive correlations were observed for NH_3_ (ρ ≈ 0.45). This positive correlation of NH_3_ should be interpreted cautiously because NH_3_ is generally considered an inhibitory byproduct in mammalian cell culture.^75,76^ The observed positive association may therefore arise from time-dependent accumulation of NH_3_ during culture, which occurs in parallel with increasing mAb titer.

**Figure 5:**
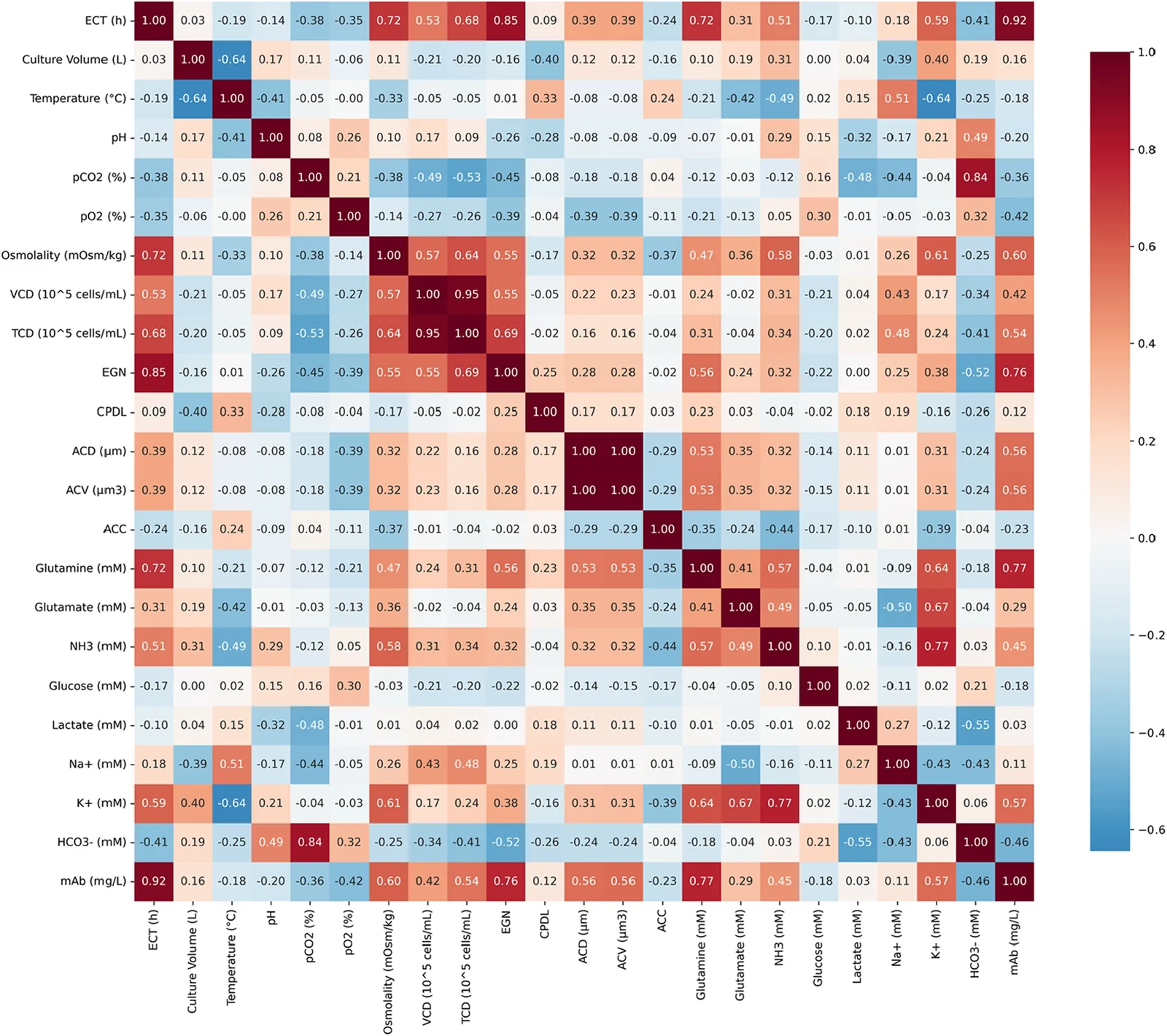
Spearman correlation matrix showing pairwise relationships among process variables and mAb titer.

**Figure 6:**
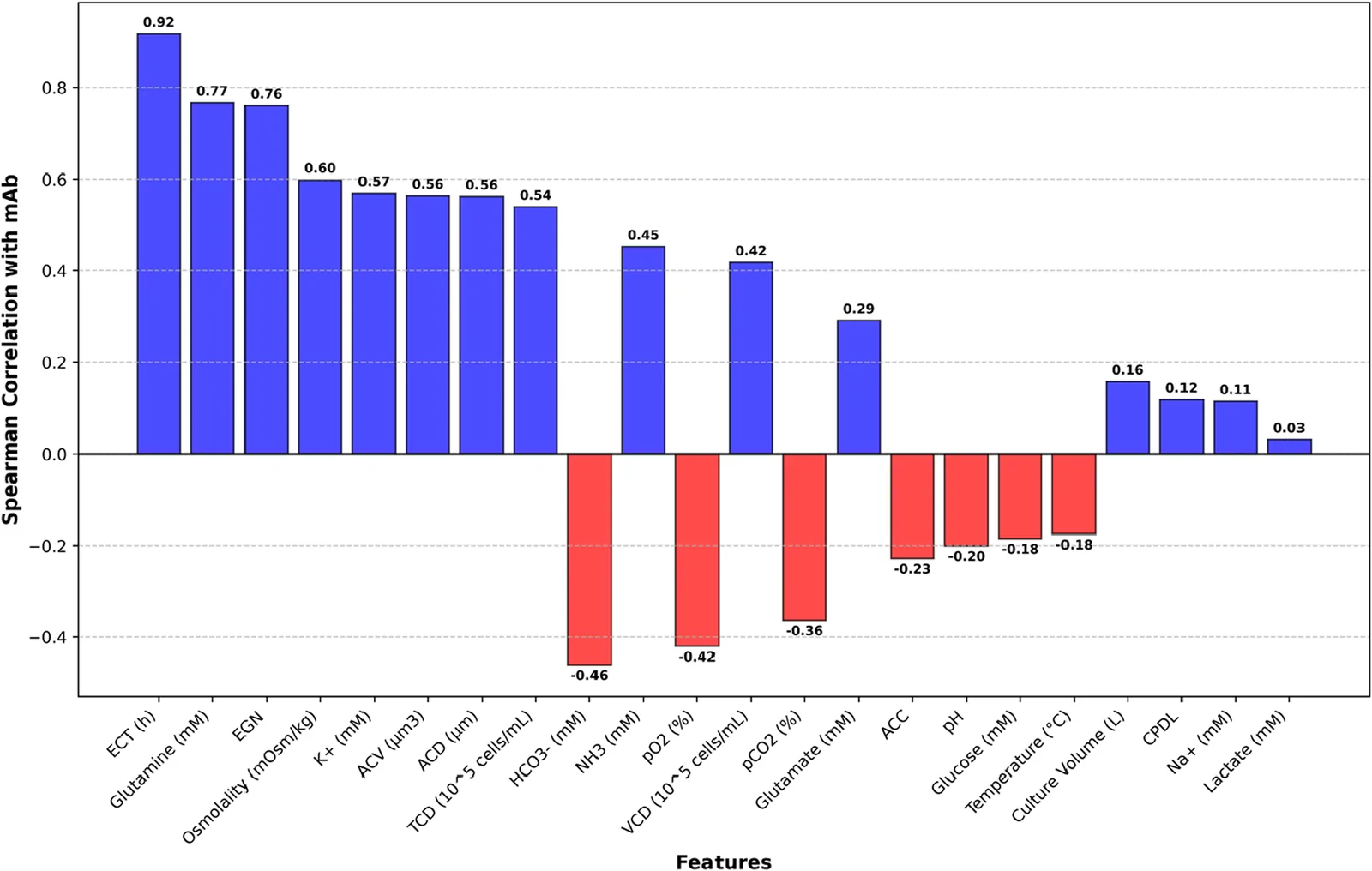
Ranked Spearman correlation coefficients of individual process variables with mAb titer.

On the other hand, HCO_3_^–^ (ρ ≈ −0.46), pO_2_ (ρ ≈ −0.42), and pCO_2_ (ρ ≈ −0.36) were moderately negatively correlated with mAb titer. Whereas pH, glucose, temperature, and other variables showed only weak associations (|ρ| < 0.3). This is likely because these parameters were tightly controlled in the bioreactor^77,78^ (temperature and pH by control loops, glucose by the feeding strategy), so there was insignificant variation left to explain differences in mAb titer.

The heatmap of correlation also revealed strong intercorrelations between process variables, which means that there is a high degree of collinearity in the process data. The variables ACV and ACD, similarly TCD and VCD, show almost similar correlation patterns with mAb as well as with other variables, indicating redundancy between the two pairs. To decrease collinearity and simplify further analyses, ACD and TCD were retained as representative variables, and ACV and VCD were excluded from further modeling. On the other hand, EGN and CPDL explain similar aspects of the cumulative cell growth. In this dataset, CPDL showed a weak correlation with mAb and did not add much information beyond EGN. Therefore, CPDL was removed and EGN was retained after imputation for the subsequent modeling.

### 3.2 Predictive Modelling for mAb Production

After data preprocessing, including the removal of outlier cultures and redundant highly correlated variables, a separate predictive-modeling analysis is conducted using the final preprocessed dataset. In this section, performance of supervised ML models is evaluated for predicting mAb titer under two relevant prediction scenarios as described in Section 2.3. Scenario 1 corresponds to the classical soft-sensing (or virtual sensing) problem of bioprocess engineering, where the concentration of the current product is predicted by using process measurements collected at the same time point. In mAb production processes, the titer is usually measured offline using techniques like high-performance liquid chromatography (HPLC) at low frequency and often with analytical delay.^35^ Several process variables such as pH, pO_2_, pCO_2_, osmolality, and cell density are measured online and in real time on a routine basis. Thus, developing a model where online and routine process variables are correlated to the actual mAb concentration can provide real-time estimation of mAb titer without needing the time-consuming analyses like HPLC. Scenario 2 focuses on predicting the final mAb titer by using only early-stage data. This kind of early prediction can be used to assess whether a production run is likely to meet specifications and to support decisions on whether to continue the culture as planned, adjust process conditions, or terminate clearly underperforming batches to avoid unnecessary resource consumption.

#### 3.2.1 Scenario 1: Pointwise Prediction of the mAb Titer

A total of 34 ML models were developed for pointwise prediction of mAb titer from the corresponding process conditions. To get optimal predictive performance of these ML models, the hyperparameters must be tuned as discussed in section 2.3.1. The hyperparameter search spaces along with the best-found settings for all models are reported in Table S4. These tuned parameters were used to predict the mAb concentration based on process conditions at each time point, and the resulting predictive performances on the training, test, and cross-validation sets are reported in Table 2.

**Table 2:** Evaluation Metrics for Training, Test, and Cross-Validation Set Performance of Single and Stacked ML Models for Pointwise Prediction of mAb Titer (Scenario 1)

	training set				test set				cross-validation performance			
model	*R* ^2^	RMSE	MAE	MAPE	*R* ^2^	RMSE	MAE	MAPE	*R* ^2^	RMSE	MAE	MAPE
RF	0.991	124.77	28.56	2.06	0.974	201.89	105.8	10.57	0.9634 ± 0.0057	243.34 ± 21.98	127.07 ± 8.25	9.28 ± 0.24
ExtraTrees	0.991	122.68	26	2.04	0.973	202.47	112.1	10.91	0.9655 ± 0.0061	236.11 ± 22.64	122.65 ± 7.85	8.98 ± 0.71
GBR	0.992	115.41	32.02	3.09	0.977	188.1	108.9	10.82	0.9632 ± 0.0039	244.46 ± 18.64	127.84 ± 10.90	9.81 ± 0.32
XGBoost	0.992	113.25	38.07	4.39	0.971	209.59	123.8	13.4	0.9645 ± 0.0019	240.52 ± 14.72	130.74 ± 9.44	11.17 ± 0.31
CatBoost	0.992	113.74	43.54	5.69	0.976	190.96	109.4	13.34	0.9660 ± 0.0040	234.79 ± 13.98	127.91 ± 8.69	11.70 ± 1.12
LightGBM	0.992	111.6	37.74	4.44	0.97	214.52	130.9	14.57	0.9673 ± 0.0027	230.47 ± 12.17	129.10 ± 5.42	11.76 ± 0.77
DT	0.965	239.43	146.9	17.77	0.931	325.65	192.3	21.91	0.9136 ± 0.0117	374.58 ± 34.46	216.29 ± 23.86	20.78 ± 1.70
KNN	0.991	122.83	27.72	2.24	0.962	242.13	139.2	13.55	0.9552 ± 0.0019	270.02 ± 12.12	147.80 ± 14.95	12.00 ± 0.69
SVR	0.961	253.64	113	9.63	0.95	277.83	152.5	17.82	0.9403 ± 0.0047	311.87 ± 22.62	161.36 ± 16.05	15.78 ± 1.60
MLP	0.972	214.05	105.3	12.48	0.954	267.54	151.9	19.16	0.9573 ± 0.0063	263.05 ± 23.64	158.75 ± 16.96	21.50 ± 1.87
LR	0.897	412.11	294.4	61.92	0.888	415.55	297.2	60.08	0.8887 ± 0.0154	424.11 ± 27.19	302.83 ± 16.04	63.21 ± 6.67
RR	0.897	412.21	294.3	61.51	0.888	415.79	297.4	59.66	0.8890 ± 0.0157	423.47 ± 27.75	302.35 ± 16.34	62.55 ± 6.22
LASSO	0.897	412.17	294.2	61.63	0.888	416.03	297.7	59.82	0.8888 ± 0.0154	423.75 ± 27.18	302.32 ± 16.11	62.74 ± 6.14
EN	0.897	412.28	294.4	61.38	0.888	415.67	297.4	59.56	0.8890 ± 0.0157	423.44 ± 27.77	302.37 ± 16.37	62.49 ± 6.21
RF + GBR + RR	0.989	131.83	59.27	5.79	0.977	189.57	102.8	10.54	0.9685 ± 0.0059	225.96 ± 27.04	123.65 ± 11.94	10.60 ± 0.53
RF + XGBoost + RR	0.992	117.17	45.56	6.31	0.975	197.23	111.7	12.44	0.9659 ± 0.0031	235.72 ± 16.98	129.37 ± 6.82	12.16 ± 1.17
RF + CatBoost + RR	0.992	112.38	44.94	7.13	0.976	192.34	114.4	13.71	0.9675 ± 0.0034	230.10 ± 19.47	129.22 ± 9.52	13.92 ± 1.35
RF + LightGBM + RR	0.992	116.23	42.05	7.29	0.976	192.99	114.1	13.92	0.9641 ± 0.0032	241.68 ± 17.65	130.52 ± 9.87	12.69 ± 0.66
ExtraTrees + GBR + RR	0.992	112.52	41.8	3.88	0.975	195.57	113.1	11.17	0.9658 ± 0.0034	236.13 ± 19.51	127.59 ± 11.59	9.99 ± 0.25
ExtraTrees + XGBoost + RR	0.992	116.51	50.52	5.47	0.972	206.61	113.9	12.75	0.9674 ± 0.0023	230.29 ± 14.19	124.24 ± 7.10	10.47 ± 0.19
ExtraTrees + CatBoost + RR	0.99	126.31	48.1	7.96	0.976	193.8	119.1	16.01	0.9641 ± 0.0071	240.14 ± 23.79	140.53 ± 9.15	16.04 ± 1.46
ExtraTrees + LightGBM + RR	0.992	112.72	42.99	4.35	0.975	194.49	111.9	12.15	0.9647 ± 0.0044	239.68 ± 22.74	127.81 ± 13.36	10.81 ± 0.66
RF + GBR + XGBoost as meta	0.985	157.76	76.54	7.56	0.972	207.47	110.9	12.76	0.9644 ± 0.0048	240.40 ± 21.71	132.31 ± 12.78	11.77 ± 0.32
GBR + CatBoost + RR	0.993	110.46	38.15	5.74	0.977	188.08	110.5	12.48	0.9673 ± 0.0040	230.09 ± 12.38	123.55 ± 7.83	12.27 ± 1.36
GBR + LightGBM + RR	0.992	113.7	46.3	7.18	0.976	193.68	114	13.85	0.9665 ± 0.0027	233.45 ± 16.83	132.60 ± 10.19	13.78 ± 0.62
ExtraTrees + GBR + LightGBM + RR	0.991	119.42	54.07	6.69	0.975	196.31	118.9	13.35	0.9656 ± 0.0045	236.53 ± 23.26	128.53 ± 12.61	10.65 ± 0.96
ExtraTrees + GBR + CatBoost + RR	0.992	113.04	45.08	6.15	0.98	175.25	107.6	12.71	0.9690 ± 0.0044	224.24 ± 18.50	122.76 ± 9.96	12.02 ± 0.60
RF + GBR + CatBoost + RR	0.991	122.91	61.85	6.78	0.979	180.51	102.2	11.72	0.9688 ± 0.0021	225.18 ± 13.37	127.27 ± 9.24	11.86 ± 0.91
RF + GBR + XGBoost + RR	0.992	118.42	52.55	5.86	0.976	190.6	111.9	12.82	0.9661 ± 0.0023	234.78 ± 12.53	127.50 ± 8.18	11.14 ± 0.33
RF + XGBoost + CatBoost + RR	0.993	106.31	44.97	7.17	0.977	189.14	115.1	13.12	0.9677 ± 0.0034	228.98 ± 13.32	127.32 ± 10.30	12.43 ± 0.80
RF + GBR + SVR + MLP + RR	0.991	122.21	62.2	7.64	0.97	216.05	124.8	14.13	0.9660 ± 0.0051	234.74 ± 21.13	127.82 ± 12.42	12.44 ± 1.26
GBR + CatBoost + LightGBM + MLP + LR	0.994	102.04	36.8	5.12	0.978	184.19	112.0	14.96	0.9691 ± 0.0042	224.27 ± 21.69	126.99 ± 11.02	13.80 ± 0.60
RF + GBR + ExtraTrees + CatBoost + RR	0.992	111.72	46.91	5.27	0.979	178.15	103.8	11.38	0.9697 ± 0.0056	221.62 ± 25.46	123.80 ± 13.70	11.81 ± 0.31
GBR + XGBoost + CatBoost + LightGBM + LR	0.989	132.97	71.64	8.15	0.976	193.89	117.6	13.21	0.9696 ± 0.0039	221.98 ± 15.52	130.06 ± 5.42	13.84 ± 0.51

Overall, tree-based ensemble methods showed better results compared to the linear and kernel-based models. More specifically, single-model ensembles RF, ExtraTrees, GBR, XGBoost, CatBoost, and LightGBM, achieved test-set *R* ^2^ values between 0.97 and 0.98, with RMSE values in the range of 180 to 215 mg/L and MAPE values mostly between 10 to 15%. On the other hand, Linear, Ridge, LASSO, and Elastic Net regressors showed noticeably lower performance (test *R* ^2^ ≈ 0.89, RMSE ≈ 416 mg/L, MAPE ≈ 60%). These patterns are consistent with the fact that the relationship between process variables and mAb titer is strongly nonlinear. Linear models are limited by their linear functional form, whereas tree-based ensemble models can better capture complex nonlinear dependencies and interactions, and improve prediction accuracy and stability by combining many decision trees.^79^

Nonlinear models, such as KNN, SVR, and MLP, achieved intermediate accuracy. The MLP regressor achieved a test *R* ^2^ of 0.95 with an RMSE of 268 mg/L and an MAPE of 19%, which is better than the linear models but worse than the best tree-based models. This suggests that these models are reasonable but not a top-performing choice compared to ensemble methods. Similar patterns have been reported in other nonlinear regression problems, where ensemble tree-based methods achieved higher accuracy and better generalization than SVMs, MLPs, and linear models for adsorption^79,80^ and composite property^81^ prediction.

As anticipated from the ensemble learning theory, the stacked ensemble models provided an additional performance gain over the individual base learners.^82–85^ In this study, the stacked configuration ExtraTrees+GBR+CatBoost+RR increased test *R* ^2^ only slightly relative to the best single ensemble (from 0.977 to 0.98), but decreased RMSE to 175.25 mg/L and MAE to 107.64 mg/L. Similarly, several other stacked models such as RF + GBR + ExtraTrees + CatBoost + RR and GBR + CatBoost + LightGBM + MLP + LR also showed satisfactory performance with test *R* ^2^ values around 0.98 and comparatively low error. This type of modest improvement in accuracy was found in the flexural bond strength prediction, where the stacked ensemble achieved an 8% gain in explained variance over the best individual model.^85^

Further, the effect of stack composition was examined by changing the number and type of the base learners. In a simple two-model stack (e.g., RF + GBR + RR), test performance was similar to the best single model, with the same test *R* ^2^ = 0.977 and a small trade-off between RMSE and MAE. Adding a third or fourth strong tree-based model gave the best results, as shown in the ExtraTrees + GBR + CatBoost + RR stack. This trend is consistent with previous studies on stacking ensembles, which reported that increasing the number of strong base learners can improve performance up to a point; however, adding too many or redundant models leads to saturation or even degradation of the ensemble.^86,87^

Many studies have shown that diversity of base learners is the key factor for improving the performance of stacking ensembles.^88,89^ In this study, stacking heterogeneity was introduced by combining different tree-based ensembles (RF, ExtraTrees, GBR, XGBoost, CatBoost, LightGBM), by adding nontree learners such as SVR and MLP, and using alternative meta-learners such as LR and XGBoost. The results showed that the nontree learners (more complex heterogeneous stacks) did not consistently improve performance compared with simpler stacks of strong tree-based models with a regularized linear meta-learner. This is because SVR and MLP exhibited moderate performance as single models, so their combination added noisy predictions, and hence, the introduction of this complex model led to marginal and inconsistent gains over the simpler tree-based stacks. Thus, to achieve optimal stacking performance, it is preferable to stack only the best-performing individual models rather than adding more models without careful selection.

Overall, the best single model was GBR, while the stacked model, ExtraTrees + GBR + CatBoost + RR, achieved the best overall performance. Figure 7 shows actual vs predicted mAb titer for selected models for Scenario 1 using GBR, CatBoost, and two stacked ensembles (ExtraTrees + GBR + CatBoost + RR, and GBR + CatBoost + LightGBM + MLP + LR). The small difference between training, cross-validation, and test *R* ^2^ values across these best-performing models indicates good generalization and limited overfitting. These findings show that nonlinear tree-based methods already capture most of the variance in mAb titer, while stacking configurations can provide an additional gain in accuracy and stability. To conclude from these results, nonlinear tree-based ensembles can be considered appropriate methods for sensing of mAb titer from online process data.

**Figure 7:**
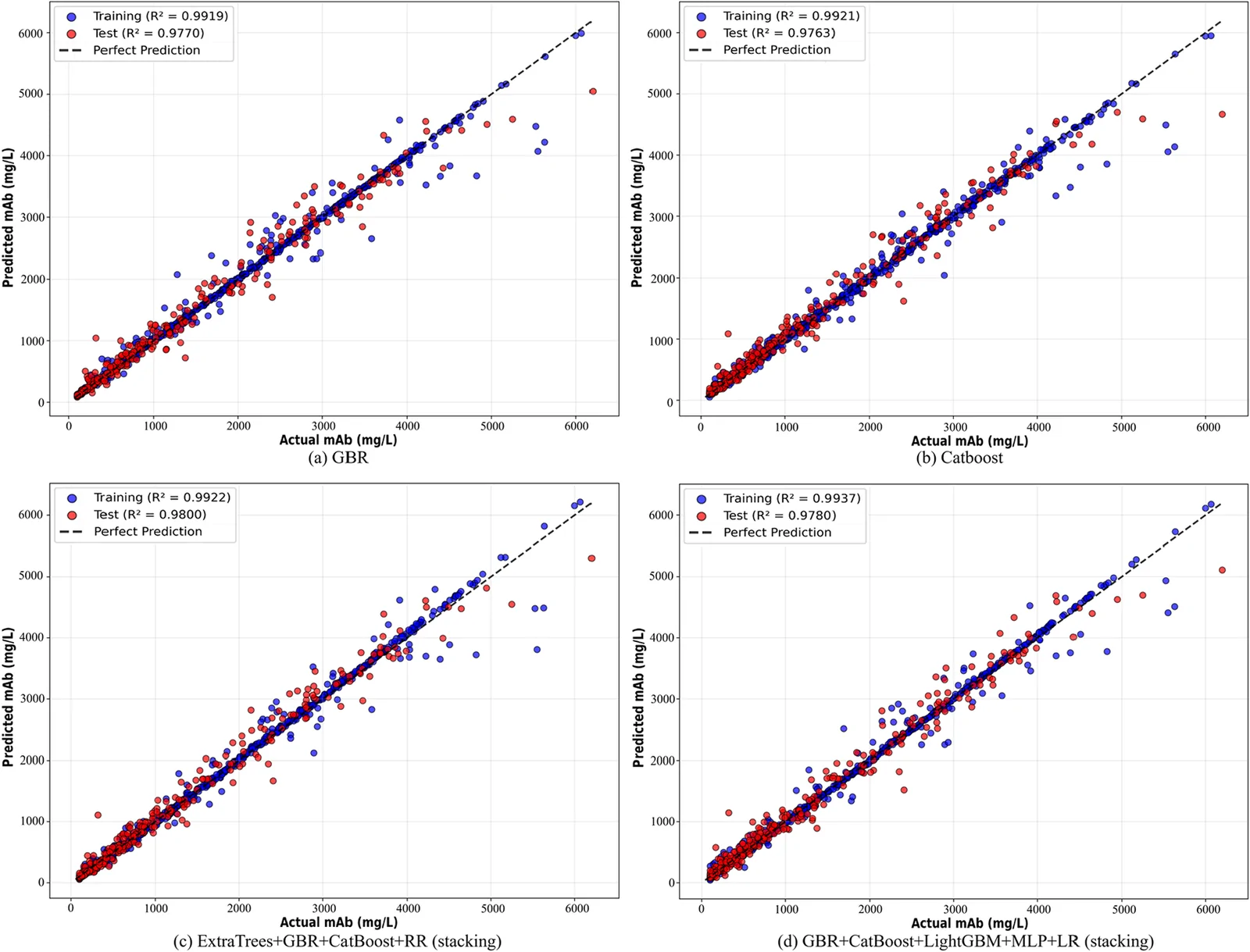
Actual vs predicted plot of mAb titer of selected models in scenario 1: (a) GBR, (b) CatBoost, (c) stacked ensemble ExtraTrees + GBR + CatBoost + RR, and (d) stacked ensemble GBR + CatBoost + LightGBM + MLP + LR.

#### 3.2.2 Scenario 2: Early-Stage Prediction of Final mAb Titer

In this scenario, the same set of 34 ML models was used to predict the final mAb titer based on data from 1 to 7 days to be used as early-stage process information. First, the hyperparameters of all models were tuned using Bayesian optimization and the best settings are reported in Table S5. These tuned model parameters were used to predict the final mAb concentration based on early process conditions. The resulting predictive performances on the training, test, and cross-validation sets are summarized in Table 3. As expected, the overall predictive accuracy was lower compared with scenario 1, indicating the greater challenge of determining the final titer using the previous process measurements.

**Table 3:** Evaluation of Metrics for Training, Test, and Cross-Validation Set Performance of Single and Stacked ML Models for Early-Stage Prediction of mAb Titer (Scenario 2)

	training set				test set				cross-validation performance			
model	*R* ^2^	RMSE	MAE	MAPE	*R* ^2^	RMSE	MAE	MAPE	*R* ^2^	RMSE	MAE	MAPE
RF	0.990	137.49	45.39	1.59	0.913	381.91	209.81	6.48	0.9303 ± 0.0356	348.23 ± 92.62	208.22 ± 50.16	7.46 ± 1.92
ExtraTrees	0.990	137.56	42.85	1.60	0.919	368.33	247.43	8.93	0.9174 ± 0.0376	383.13 ± 82.60	259.02 ± 47.20	9.89 ± 1.50
GBR	0.989	141.54	57.08	2.16	0.913	381.57	221.52	6.90	0.9353 ± 0.0272	338.00 ± 74.02	209.92 ± 40.66	7.51 ± 1.34
XGBoost	0.990	134.74	50.43	1.96	0.907	393.45	230.34	7.78	0.9356 ± 0.0200	342.63 ± 51.91	217.07 ± 35.44	8.03 ± 0.92
CatBoost	0.988	151.94	49.40	1.79	0.925	353.87	239.48	8.43	0.9121 ± 0.0335	396.83 ± 75.48	274.32 ± 46.13	10.63 ± 1.65
LightGBM	0.991	128.39	48.15	1.83	0.930	341.77	233.07	8.18	0.9330 ± 0.0276	346.11 ± 71.83	243.16 ± 43.28	9.21 ± 1.61
DT	0.941	333.87	175.34	5.47	0.752	642.99	324.74	9.79	0.8572 ± 0.0505	508.08 ± 92.44	291.57 ± 71.51	9.72 ± 2.18
KNN	0.979	201.43	62.99	2.20	0.864	476.64	347.24	12.02	0.8448 ± 0.0386	535.87 ± 70.61	372.69 ± 44.67	15.04 ± 3.67
SVR	0.899	436.32	277.52	11.56	0.855	491.43	371.47	14.26	0.8398 ± 0.0381	544.30 ± 64.59	389.74 ± 41.91	17.58 ± 4.01
LR	0.714	734.66	558.67	26.09	0.698	710.39	553.12	25.69	0.6703 ± 0.0236	785.40 ± 23.25	594.64 ± 15.16	27.88 ± 4.39
RR	0.666	793.66	613.05	30.78	0.645	770.50	592.86	28.88	0.6341 ± 0.0170	828.31 ± 31.71	643.18 ± 22.31	32.62 ± 5.18
LASSO	0.678	779.52	592.17	28.88	0.665	747.97	578.44	27.68	0.6532 ± 0.0236	805.67 ± 25.37	610.81 ± 29.65	29.77 ± 3.67
EN	0.653	808.88	627.52	31.86	0.629	786.98	605.36	29.74	0.6320 ± 0.0168	830.64 ± 32.03	645.49 ± 22.72	32.79 ± 5.23
MLP	0.967	248.14	139.33	5.58	0.825	540.27	389.56	14.27	0.8419 ± 0.0326	540.60 ± 52.26	385.98 ± 41.14	15.50 ± 1.13
RF + GBR + RR	0.989	145.52	68.50	2.89	0.909	389.82	219.59	7.00	0.9324 ± 0.0290	346.83 ± 75.79	211.13 ± 44.17	7.59 ± 1.69
RF + XGBoost + RR	0.986	163.96	115.43	4.99	0.929	345.02	217.72	7.98	0.9439 ± 0.0239	316.93 ± 67.89	210.04 ± 39.38	8.32 ± 1.28
RF + CatBoost + RR	0.987	159.25	100.86	4.37	0.924	357.22	233.09	8.08	0.9344 ± 0.0268	342.25 ± 70.75	225.41 ± 38.30	8.52 ± 1.42
RF + LightGBM + RR	0.990	137.55	57.05	2.35	0.913	381.31	213.71	6.65	0.9301 ± 0.0380	347.71 ± 98.14	206.24 ± 55.23	7.44 ± 2.16
ExtraTrees + GBR + RR	0.989	147.04	85.63	3.37	0.909	390.66	240.99	7.72	0.9368 ± 0.0269	335.41 ± 69.71	220.23 ± 35.15	7.67 ± 1.02
ExtraTrees + XGBoost + RR	0.994	111.08	50.55	2.23	0.921	363.58	226.48	7.83	0.9448 ± 0.0251	313.79 ± 69.36	195.52 ± 36.89	7.33 ± 1.12
ExtraTrees + CatBoost + RR	0.990	136.75	70.53	3.31	0.915	377.41	243.11	8.22	0.9264 ± 0.0350	360.47 ± 82.78	247.40 ± 42.53	9.17 ± 1.23
ExtraTrees + LightGBM + RR	0.991	132.80	72.80	3.12	0.932	336.88	228.21	8.23	0.9316 ± 0.0307	348.33 ± 78.53	243.38 ± 47.58	9.19 ± 1.55
RF + GBR + XGBoost as meta	0.968	245.68	168.66	6.53	0.915	377.67	250.37	8.61	0.9199 ± 0.0315	377.36 ± 77.60	262.69 ± 43.44	9.82 ± 1.19
GBR + CatBoost + RR	0.990	136.25	76.25	3.11	0.915	375.76	226.33	7.52	0.9284 ± 0.0360	353.83 ± 87.21	244.76 ± 47.68	9.19 ± 1.33
GBR + LightGBM + RR	0.989	143.06	82.44	3.18	0.906	395.58	237.52	7.62	0.9346 ± 0.0265	341.62 ± 69.92	222.15 ± 36.54	7.80 ± 1.24
ExtraTrees + GBR + LightGBM + RR	0.991	132.80	69.90	2.85	0.913	380.24	227.48	7.45	0.9313 ± 0.0333	346.81 ± 86.33	219.54 ± 50.82	7.72 ± 1.71
ExtraTrees + GBR + CatBoost + RR	0.989	144.230	88.010	3.410	0.906	396.610	239.720	7.550	0.9407 ± 0.0235	326.27 ± 64.27	213.87 ± 30.14	7.53 ± 0.90
RF + GBR + CatBoost + RR	0.988	150.410	86.300	4.210	0.919	367.720	228.720	7.820	0.9336 ± 0.0293	342.56 ± 77.12	219.82 ± 42.56	7.96 ± 1.47
RF + GBR + XGBoost + RR	0.991	127.800	61.140	2.340	0.934	332.910	209.730	6.700	0.9356 ± 0.0343	334.02 ± 86.93	209.06 ± 47.53	7.33 ± 1.35
RF + XGBoost + CatBoost + RR	0.990	136.880	69.000	3.030	0.912	384.470	222.690	7.140	0.9350 ± 0.0284	339.60 ± 75.33	214.39 ± 39.27	7.79 ± 1.32
RF + GBR + SVR + MLP + RR	0.989	145.180	71.800	2.890	0.909	390.750	218.620	6.770	0.9414 ± 0.0226	323.33 ± 64.22	201.17 ± 36.20	7.21 ± 1.07
GBR + CatBoost + LightGBM + MLP + LR	0.990	135.040	67.460	2.800	0.918	370.240	227.850	7.410	0.9357 ± 0.0310	334.85 ± 83.76	212.36 ± 45.28	7.49 ± 1.53
RF + GBR + ExtraTrees + CatBoost + RR	0.981	191.460	132.860	4.760	0.912	384.000	245.160	7.590	0.9436 ± 0.0269	315.89 ± 73.61	212.75 ± 37.84	7.77 ± 1.28
GBR + XGBoost + CatBoost + LightGBM + LR	0.989	141.630	95.690	3.780	0.915	376.110	228.200	7.680	0.9398 ± 0.0327	324.47 ± 87.70	212.68 ± 49.82	8.27 ± 1.78

Similar to scenario 1, nonlinear tree-based ensembles showed better results than linear and kernel-based approaches among single models. Even though the training *R* ^2^ values were close to one; RF, ExtraTrees, GBR, XGBoost, CatBoost, and LightGBM achieved test-set *R* ^2^ values in the range of 0.91 to 0.93; with RMSE values of about 340–395 mg/L; and MAPE values between 6 to 9%. In contrast, linear models (LR, RR, Lasso, and EN) showed poorer performance with test *R* ^2^ values of ≈0.63 to 0.70, RMSE around 710–790 mg/L and MAPE of ≈26–30%. Other nonlinear models such as KNN, SVR, and MLP showed intermediate performance.

The stacked ensemble models gave a modest improvement over the individual tree-based learners. Some combinations of multiple tree-based models with a ridge meta-regressor, such as RF + GBR + XGBoost + RR, ExtraTrees + LightGBM + RR, and ExtraTrees + XGBoost + RR, achieved test-set *R* ^2^ values between 0.92 and 0.93, with RMSE values of 330 to 380 mg/L, and MAPE values of about 7 to 9%. These stacking models showed slightly higher and more stable cross-validation *R* ^2^ values between 0.93 to 0.94 compared to their base models. This points to small improvements in robustness, rather than dramatic boosts in accuracy. The actual vs predicted plot of predicted final mAb titer for selected best models in this scenario (LightGBM, CatBoost, ExtraTrees + LightGBM + RR, and RF + GBR + XGBoost + RR) is shown in Figure 8.

**Figure 8:**
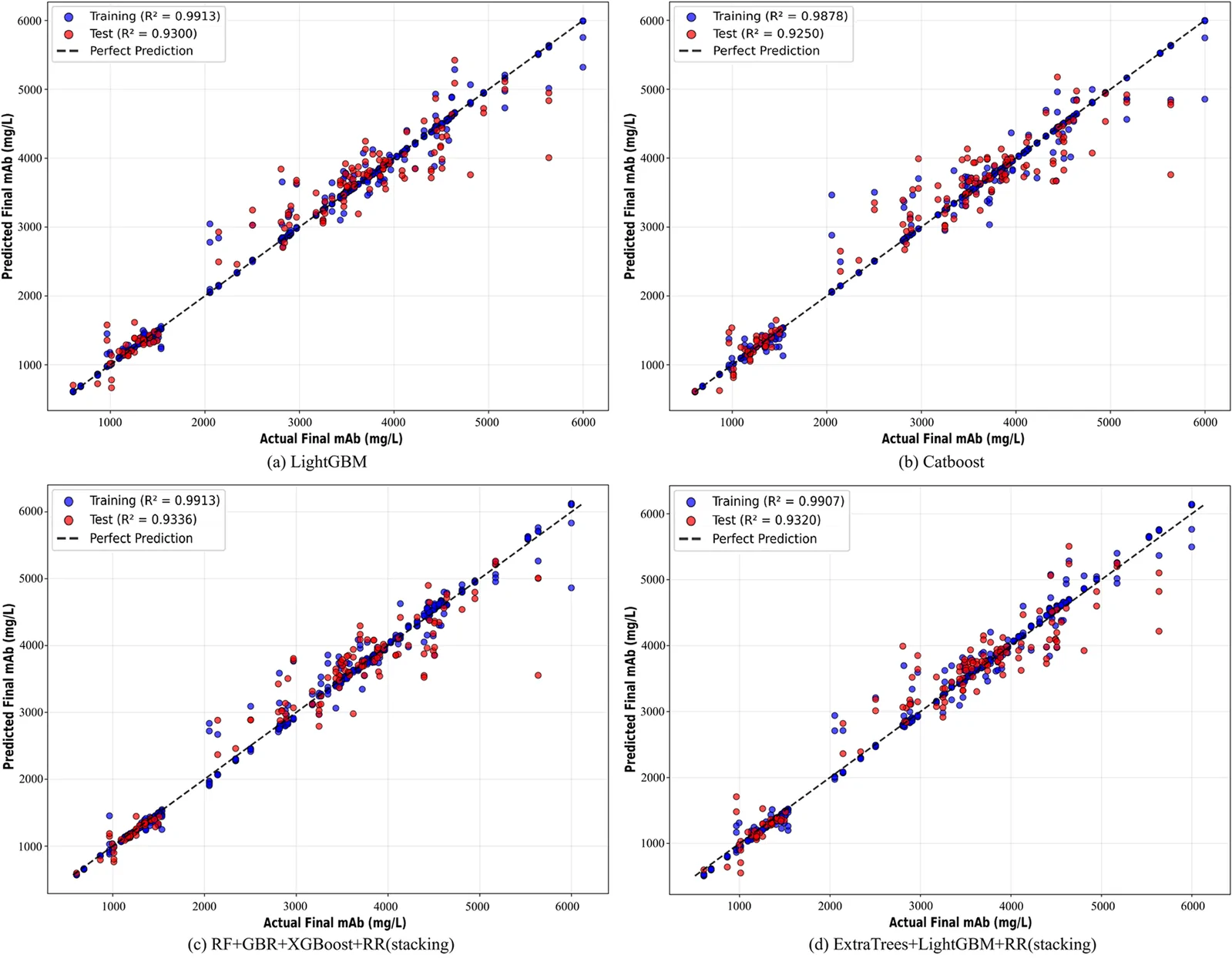
Actual vs predicted plot of final mAb titer for selected models in Scenario 2: (a) LightGBM, (b) CatBoost, (c) stacked ensemble RF + GBR + XGBoost + RR, and (d) stacked ensemble ExtraTrees + LightGBM + RR.

The effect of stack composition in scenario 2 was consistent with the result of scenario 1. The stack comprising three strong tree-based ensembles (e.g., RF + GBR + XGBoost + RR) with a ridge meta-regressor gave the best trade-off between accuracy and robustness, although only modest improvements in test *R* ^2^ were observed over the best single model, with lower RMSE and a little higher cross-validation *R* ^2^. Incorporating diverse base learners like SVR and MLP did not further improve performance and, in some cases, decreased the test performance, indicating saturation of the ensemble. A similar observation was reported in a study, where the addition of heterogeneous models such as SVM, KNN, and MLP did not improve performance once strong tree-based learners are already included.^90^ Therefore, in this early prediction scenario as well, stacking of best-performing tree-based learners is more effective than building very heterogeneous or very large ensembles.

For the best models, the training *R* ^2^ values were close to 0.99, whereas test and cross-validation *R* ^2^ values were about 0.90 to 0.94. This suggests that process data from 1 to 7 days are not much capable of predicting all factors that determine final mAb titer. This may be due to the fed-batch bioreactor operation and the intermittent adjustment of nutrients later in the process. Overall, nonlinear tree-based methods already achieve most of the explainable variance, and stacked ensembles provide only small additional gains in the accuracy and stability. In conclusion, for the early prediction task, the accuracy is lower but adequate to give valuable insight into the final batch performance and to support early decisions on process continuation, adjustment, or termination.

Collectively, these two scenarios provide quantitative evidence for the two industrially relevant tasks (i) using online or routinely measured process data to estimate the present mAb level in real-time (soft sensing), and (ii) predicting the final titer in advance to support proactive monitoring and control of a process. In Scenario 1, the best stacked model, ExtraTrees + GBR + CatBoost + RR, achieved a test *R* ^2^ of 0.980 for pointwise mAb prediction. This supports the use of routinely measured process variables for near-real-time estimation of current mAb titer and may reduce reliance on frequent offline HPLC measurements. In Scenario 2, the best stacked model, RF + GBR + XGBoost + RR, achieved a test *R* ^2^ of 0.934 for final titer prediction using only data from days 1–7. This demonstrates that early-stage process data contains sufficient information to support earlier feedback prediction on final batch performance. Therefore, the two scenarios together support the practical value of the workflow for real-time titer estimation, early feedback, and data-driven decision-making in upstream mAb process development. Since the same methodology for preprocessing, selection of models, hyperparameter tuning, validation, and testing was used to evaluate 34 models across two prediction tasks, the proposed modelling workflow can be reused for related upstream bioprocess prediction tasks.

### 3.3 Model Interpretability and Sensitivity Analysis

Following the prediction of mAb titer in scenario 1, previously described model interpretability and sensitivity analyses (PVI, SHAP, and PDA) were applied to the best-performing models. In particular, the analysis focuses on gradient boosting (which was the best among the single models) and stacked ensemble, ExtraTrees+GBR+CatBoost+RR (which was the best configuration among the stacked models). These results are presented in the following sub-sections.

#### 3.3.1 Permutation Variable Importance Analysis

The contribution of each of the process variables to the predictive performance of the models was quantified by permutation variable importance described earlier in Section 2.4.1. The two best models mentioned above were compared and analyzed using the independent test set, and their permutation importance for the top 15 variables is presented in Figure 9.

**Figure 9:**
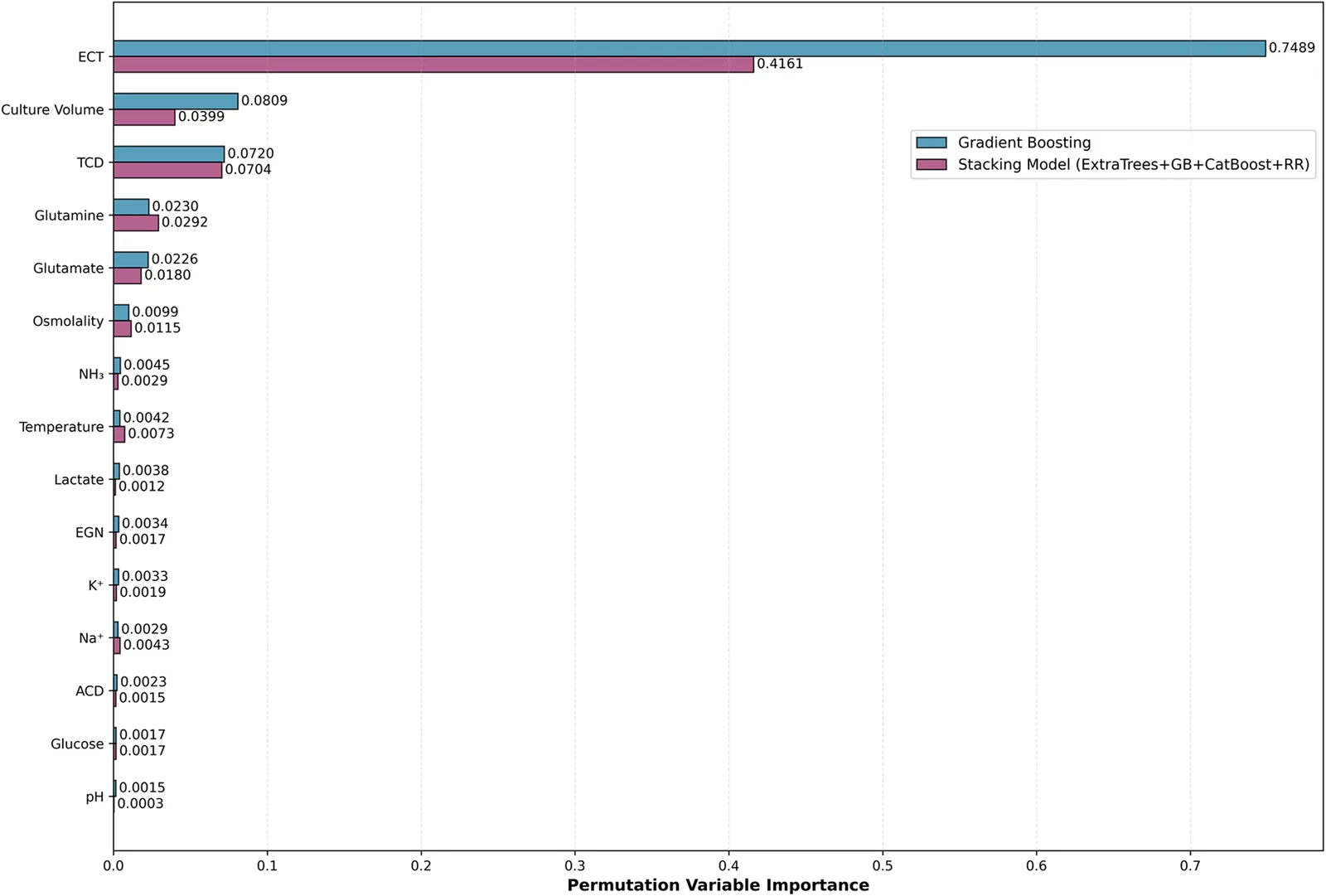
Permutation-based feature importance of the 15 top process variables for mAb titer prediction using GBR and stacking models.

In both models, the ECT was by far the strongest predictor of mAb titer. Permutation of ECT in the GBR model caused an average decrease in test-set *R* ^2^ of about 0.75, while in the stacking model the PVI was about 0.42. These values were an order of magnitude higher than those of any other variable, which demonstrates that effective culture time is the strongest driver of mAb predictions. This is consistent with the analysis of Hagel et al. that longer culture times can yield higher titers.^91^ Further, the culture volume and TCD showed noticeable PVI values, whose permutation leads to a moderate decrease in *R* ^2^, which indicates that overall culture volume and viable cell density play a key role in determining the amount of product formed. This widely reported observation that achieving higher viable cell densities in fed-batch processes leads to increased production titers.^92,93^ Glutamine, glutamate, and osmolality have smaller but non-negligible PVI values, suggesting that nutrient availability and the integrated physicochemical state of the culture also contribute to the prediction accuracy. The remaining variables exhibited only small PVI values in both models, which means they function as tertiary contributors to the prediction task.

Overall, the PVI analysis shows that both the single GBR model and the stacking ensemble depend on a common set of biologically relevant variables like ECT, culture volume, TCD, glutamine, glutamate, and osmolality to obtain their predictive performance and that other process variables have only a small contribution to the prediction of mAb titer. The stacking ensemble offered a small accuracy gain over GBR because it combines several complementary tree-based learners and relies on a fundamentally diverse set of input variables.

#### 3.3.2 Shapley Additive Explanation Analysis

Both the gradient boosting and the stacked models were analyzed through SHAP analysis to further understand how the predicted mAb titer varies with key process variables. The global feature importance was measured in all of the samples by the mean absolute SHAP value of each variable (Figure 10). SHAP summary (beeswarm) plots are used to visualize the distribution of SHAP values across all samples and to identify the direction of effect and nonlinearities (Figure 11).

**Figure 10:**
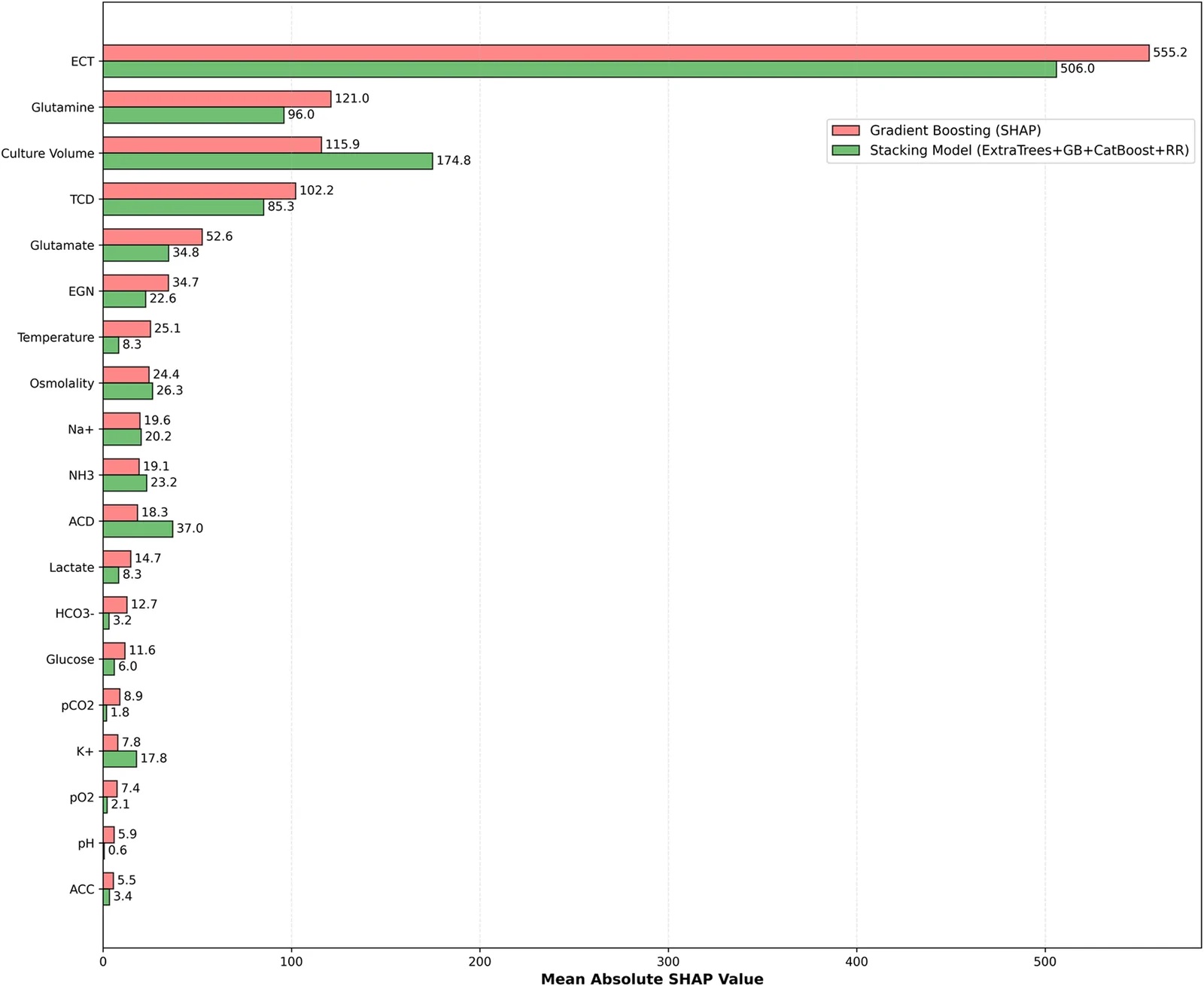
Global feature importance based on the mean absolute SHAP value of each process variable for the best models.

**Figure 11:**
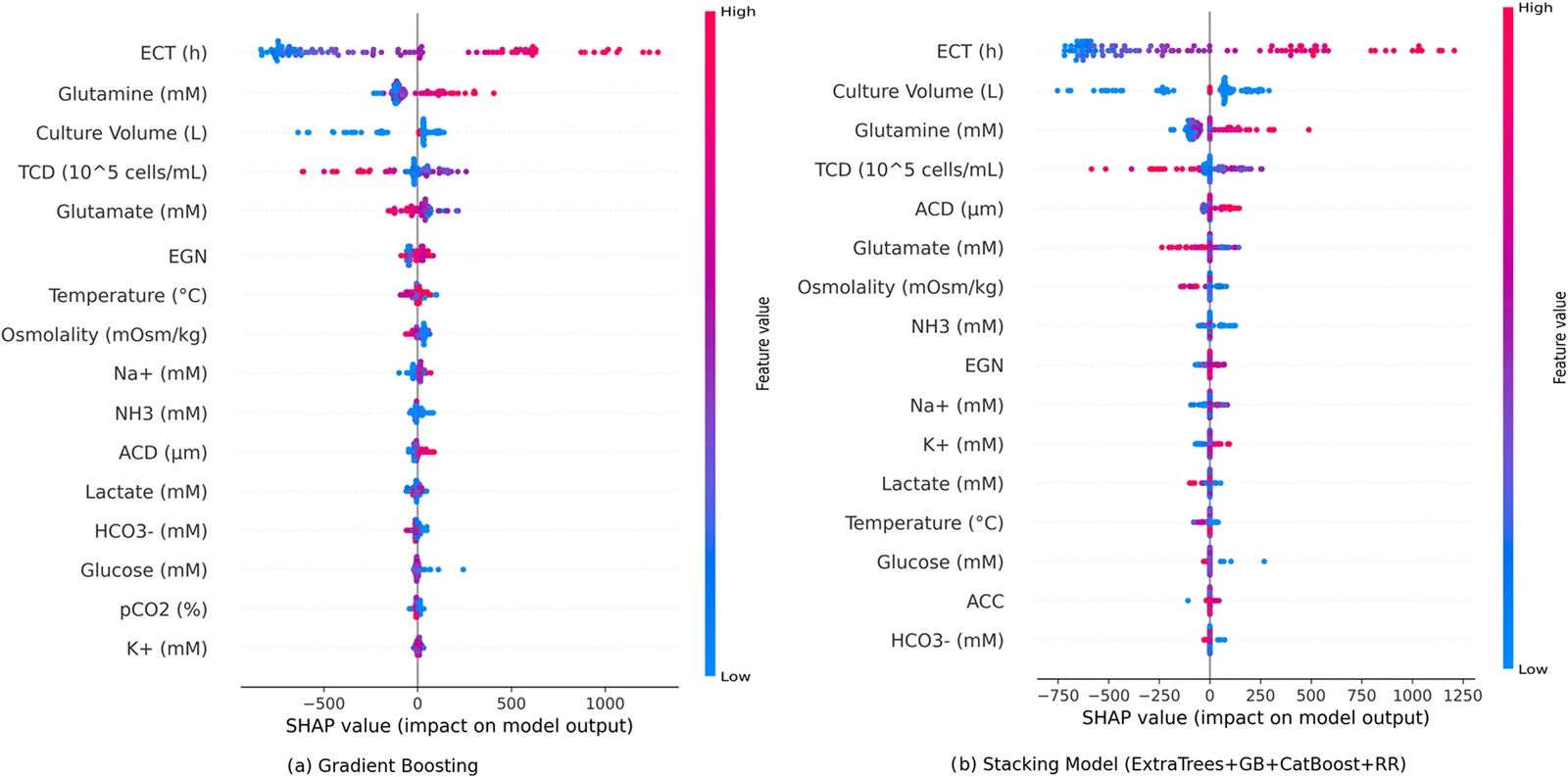
SHAP summary (beeswarm) plot of each process variable for the best models. Each point represents the SHAP value of a feature for a single sample colored by the corresponding feature value.

The global SHAP ranking closely matched the PVI results. In both models, ECT was again the dominant variable, with mean absolute SHAP values being many folds greater than the values of other features. Glutamine, culture volume, TCD, and glutamate formed a second level of important variables, followed by EGN, temperature, and osmolality, which showed moderate but consistent contributions. Other variables such as NH_3_, Na^+^, K^+^, and lactate had significantly smaller mean absolute SHAP values, which means that they are secondary in determining the predictions. Glucose, pH, pO_2_, and HCO_3_^–^ were among the least influential variables in both models, in line with their low PVIs. These ranking trends are consistent with previous reports, which highlighted the importance of culture duration, nutrient status, and cell-growth-related variables in mAb production.^34,69,70,73,75,91^

The SHAP summary plots gave further information about the directions of these effects. In ECT, low values (early culture times) were inversely correlated with negative SHAP values, whereas high ECT values generated positive SHAP values, which is the anticipated increase in mAb titer during the period of the culture.^35,69^ For glutamine, a higher concentration was likely to be associated with increased productivity as it helps in supporting cell growth and productivity. This is biologically reasonable because glutamine supports CHO cell growth, tricarboxylic acid cycle activity, and biosynthetic pathways linked to productivity.^94–96^ Glutamate showed a weaker directional trend but slightly increased predicted titer at higher concentrations, which is consistent with its role in central metabolism and productivity-related pathways.^97,98^ On the other hand, higher osmolality values were linked to negative SHAP values, indicating reduced predicted titer due to growth-inhibiting effects. This is consistent with the reports that hyperosmolality can inhibit CHO cell growth and induce stress responses.^34,99^ The controlled variables (i.e., glucose- and pH-related variables) had SHAP values that were tightly clustered around zero, suggesting that these variables were employed within the limited operating ranges.

These results are broadly consistent with the correlation analysis presented in Section 3.1.3. Variables that showed high positive correlations with mAb titer (ECT, glutamine, TCD, and osmolality) have high permutation importance and SHAP values, indicating that the models effectively exploit these relationships. On the other hand, some of the variables known to be biologically significant in mAb production (glucose, pH, NH_3_, and pO_2_) showed low PVI and low mean absolute SHAP values. Moreover, this result is in line with the description of the process provided by Gandharan et al.,^30^ who applied the same dataset. They reported that glucose and pH were actively controlled within a narrow range of values, and therefore, the related online profiles did not provide much data on the limiting and inhibitory conditions. Similarly, NH_3_ concentrations and dissolved oxygen are mechanistically important, but both were tightly controlled. Thus, varying glucose, pH, NH_3_, and pO_2_ does not cause a considerable influence on the titer of mAb under these operating conditions, even though they are mechanistically important to cell growth and metabolism.

#### 3.3.3 Analysis of Partial Dependence Plots

Further, PDA was conducted to visualize the effect of individual process variables on the model predictions of the mAb titer. In these plots, the curve shows the average model prediction for a given variable, while the effect of all other variables is averaged over their empirical distributions. PDA was performed for the best models, and both models provided similar results for the process variables. Thus, only the results of the stacked ensemble model are reported. The 1D-PDPs for all variables are provided in Figure 12.

**Figure 12:**
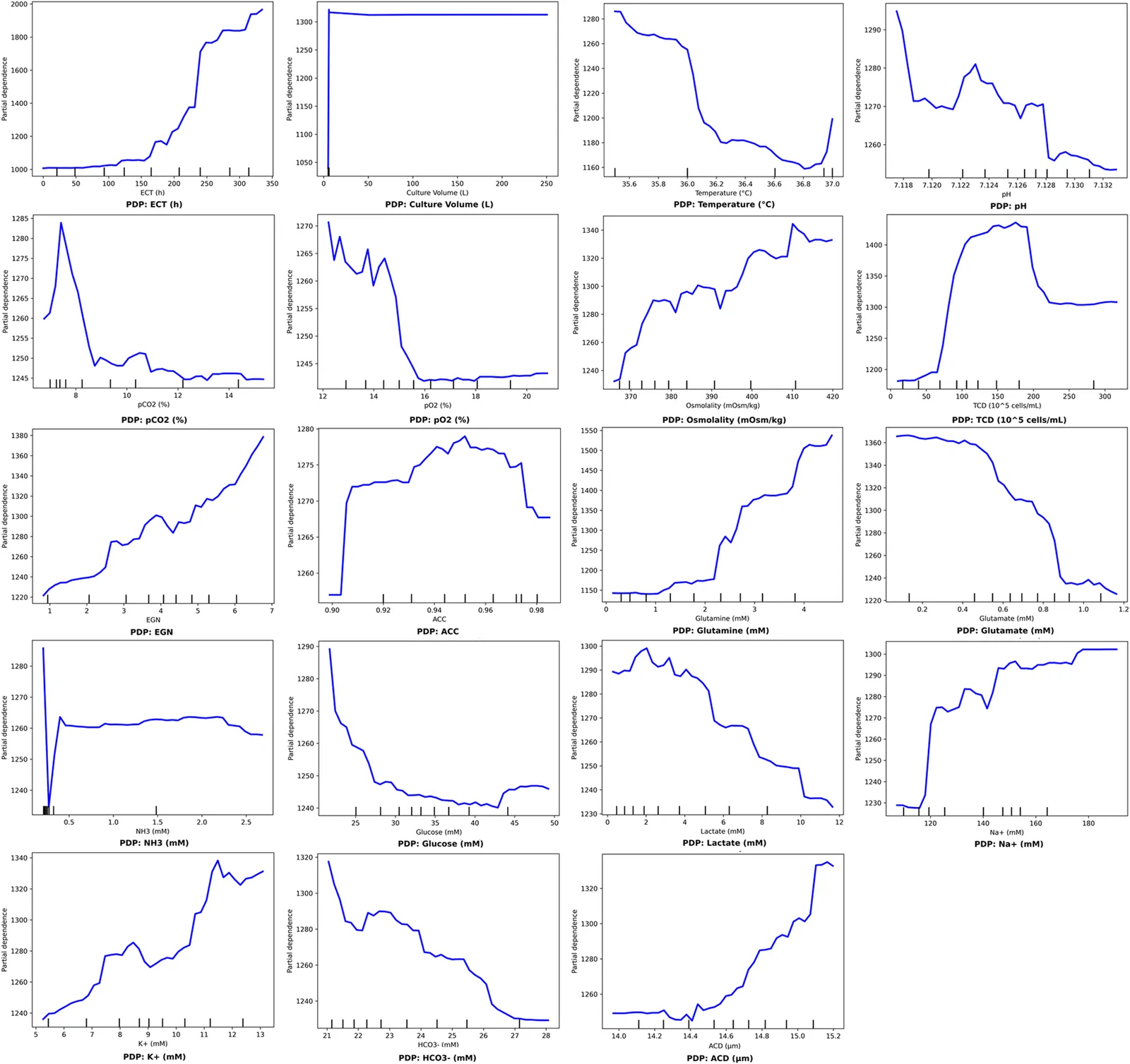
1D-PDPs for the key process variables. Each plot shows how the corresponding variable influences the predicted mAb titer, with the remaining variables averaged over their empirical distributions.

The most important variable, ECT, showed a monotonically increasing relationship with predicted mAb titer. This indicates that increasing culture time can produce larger mAb titers provided that cell health (viability) was maintained and nutrients were not strongly limiting.^35,36^ In the case of TCD, the mAb titer was low at very low TCD, highest at an intermediate TCD, and then decreased again at very high TCD. The 2D-PDP of ECT and TCD (Figure 13) illustrated this combined effect, with the highest predicted titers occurring at longer ECT and moderate TCD, whereas shorter ECT or higher TCD resulted in lower titers. This is consistent with the reports that culture becomes nutrient-limited at very high TCD and is affected by the accumulation of waste products such as lactate, NH_3_, and CO_2_.^34–36^ Further, PDP of EGN showed low predicted titer at small EGN and then increased steadily with the increase in EGN. This implies that the cells are undergoing several generations from pregrowth state to a more production state as ECT increases, which is clearly seen in the 2D-PDP of ECT and EGN (Figure 14).

**Figure 13:**
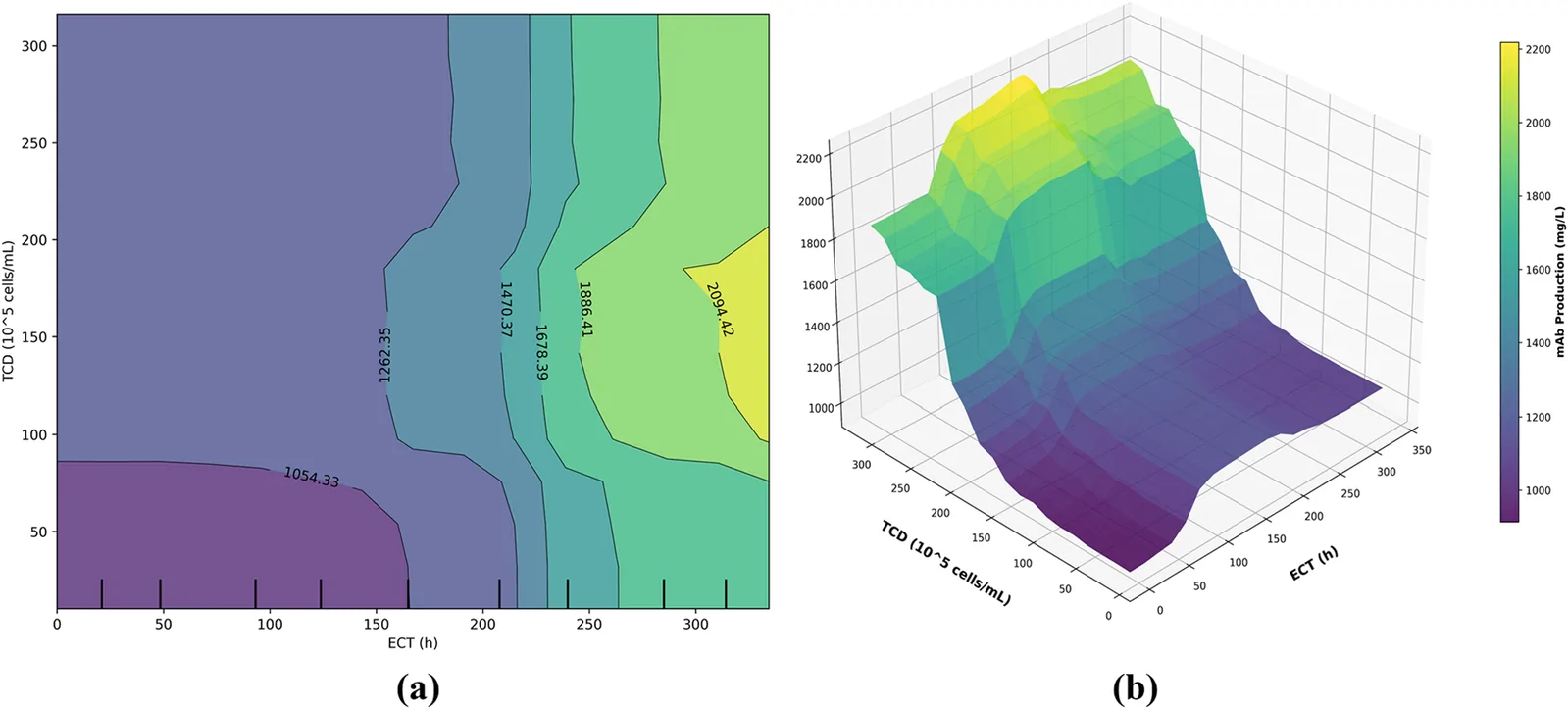
2D-PDP of ECT and TCD on the predicted mAb titer: (a) 2D contour PDP and (b) 3D surface.

**Figure 14:**
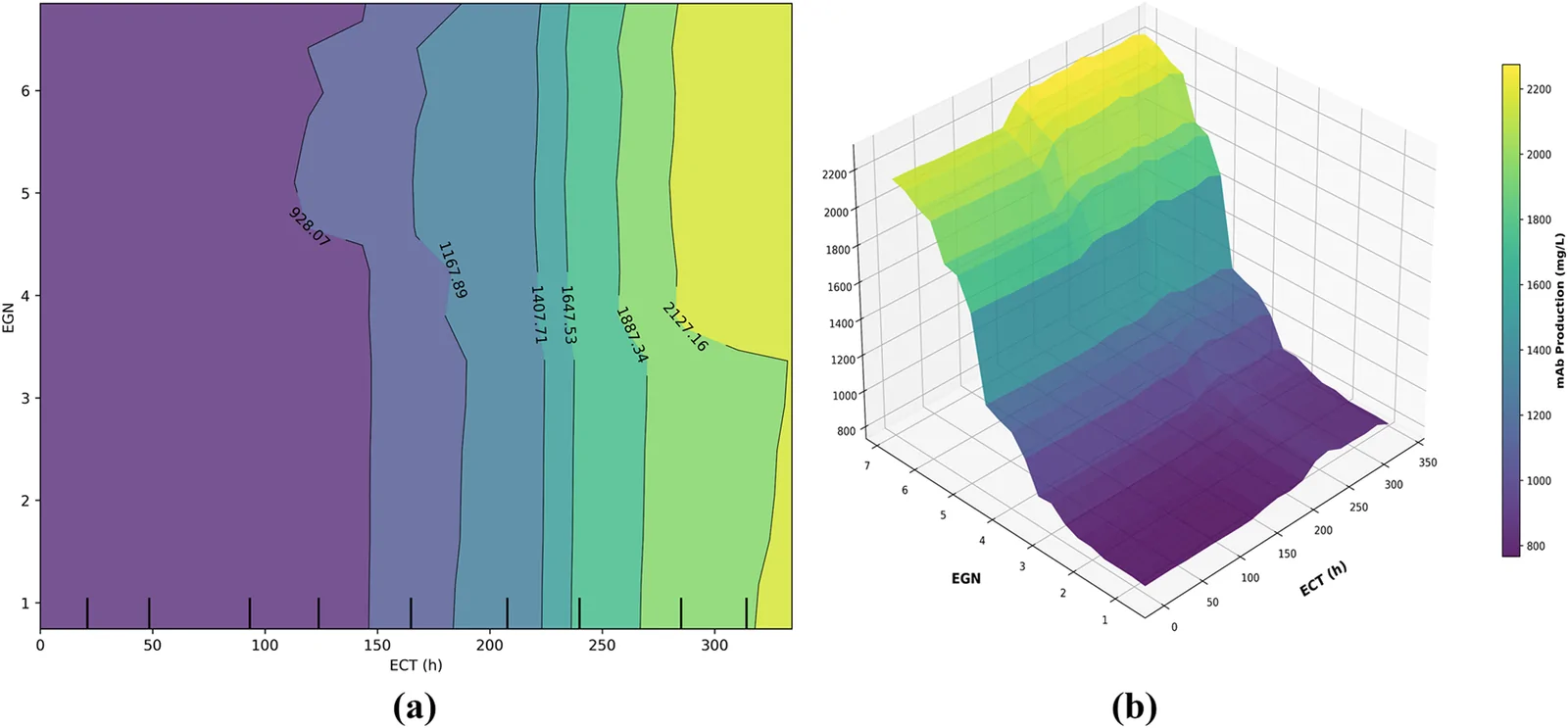
2D-PDP of ECT and EGN on the predicted mAb titer: (a) 2D contour PDP and (b) 3D surface.

Cell-state variables showed patterns that are consistent with bioreactor physiology. The PDP of ACD showed a typical cell growth profile, with constant prediction at small cell sizes and increased titer at large diameters. The higher titer corresponds to the late exponential and stationary phases, when cells reduce the rate of division and spend more energy on production. The PDP of ACC exhibited rapid growth in the mAb titer prediction as ACC changed between 0.90–0.91 and was followed by a wide plateau. This suggested that cells with a moderate level of compactness are the most productive, whereas very low or extreme cell compactness indicates less favorable physiological conditions. Culture volume has an almost flat PDP, as expected, because the mAb titer was expressed as concentration (per unit volume) and all scales were run under the same conditions.

In nutrient management, glucose is the main carbon and energy source for the CHO cells. The PDP showed the highest predicted mAb at a low glucose level, with titer decreasing as glucose concentration increased. In a typical run, glucose is high early in the run and is consumed as cells grow and produce mAb. This suggests that keeping glucose at a controlled level helps cells use energy more efficiently and make more product, while higher glucose drives wasteful glycolysis, producing excess lactate, raising osmolality, and reducing the overall efficiency.^35^ The PDP for lactate showed that titers increased when lactate increased from 0 to 2 mM lactate and then dropped sharply at higher levels. This suggested that a small amount of lactate is simply a sign of active growth, but higher accumulation is associated with reduced growth, changed glycosylation, and lower productivity.^34,35,37^ This combined behavior is captured in the 2D-PDP, Figure 15, where the highest titers occur at low glucose with low-to-moderate lactate, while high glucose and high lactate are associated with reduced titers.

**Figure 15:**
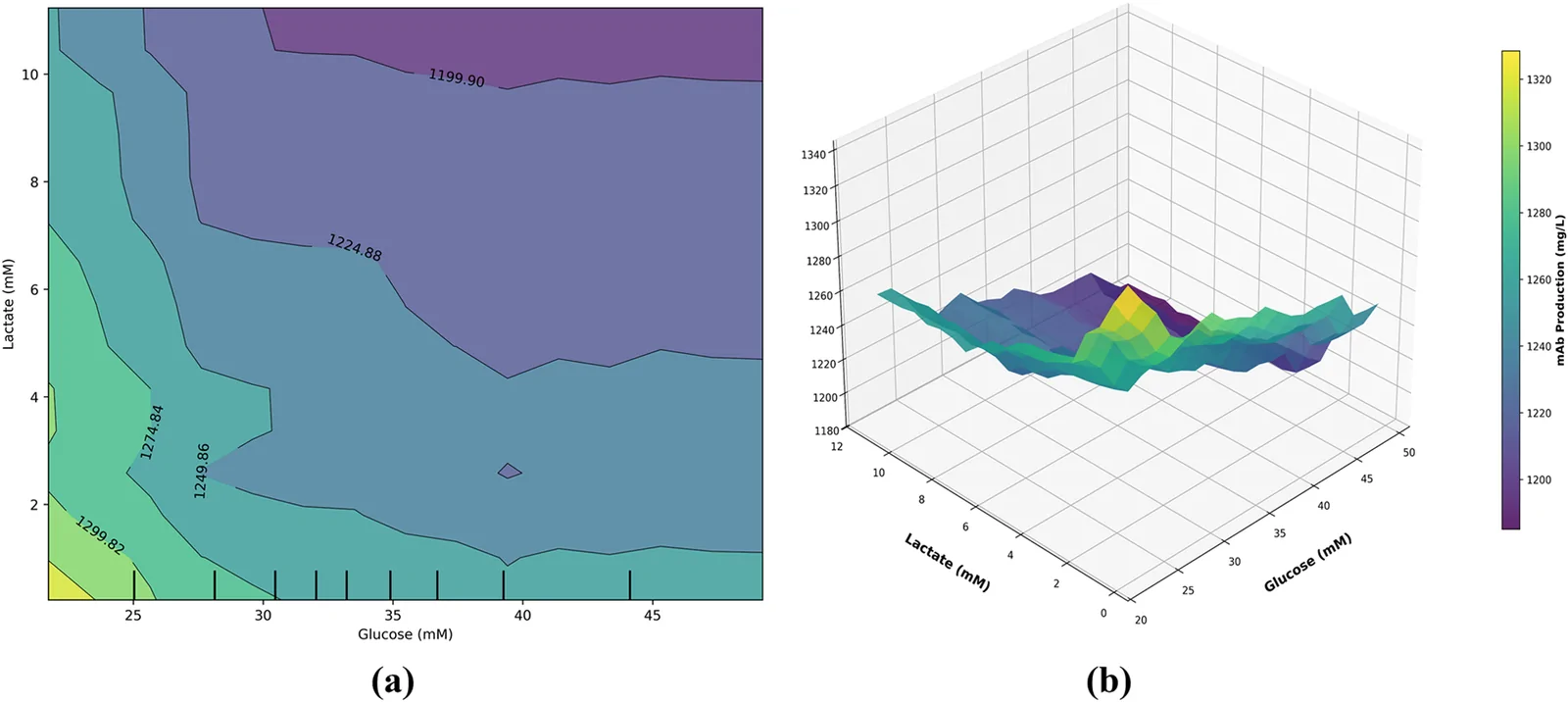
2D-PDP of glucose and lactate on the predicted mAb titer: (a) 2D contour PDP and (b) 3D surface.

Nitrogen metabolism variables showed similar trends. The PDP of glutamine showed that titers increased steadily as the glutamine concentration increased. This suggested that more glutamine within this range supported better growth and biosynthesis. This is consistent with the reports that glutamine depletion tends to lead to poorer metabolic and glycosylation states^37^ and that glutamine is a major nitrogen and energy source in high specific productivity.^35^ The glutamate PDP is flat at low concentrations; however, predicted titers decreased with an increase in glutamate. This suggested that high glutamate comes from stressed cultures grown under nutrient-imbalanced conditions, where nitrogen metabolism is disturbed, and the mAb productivity is reduced. This agrees with the reports that healthy cultures tend to consume glutamate, whereas imbalanced cultures produce it.^34,37^ The PDP for NH_3_ showed a steep drop. Predicted titers were highest when the NH_3_ concentration was zero. Even small increases above 0.2 to 0.3 mM sharply reduced the prediction, after which it stayed low. The 2D-PDP of glutamine and NH_3_ were presented in Figure 16, which showed that the highest titers were only obtained when glutamine was sufficiently high and NH_3_ remained close to zero. However, regions with elevated NH_3_ showed reduced titers even at high glutamine. This means that any accumulation of NH_3_ was harmful for growth and productivity and is consistent with reports that a high level of NH_3_ impacts cell growth and modifies the mAb glycosylation.^34,35,37^

**Figure 16:**
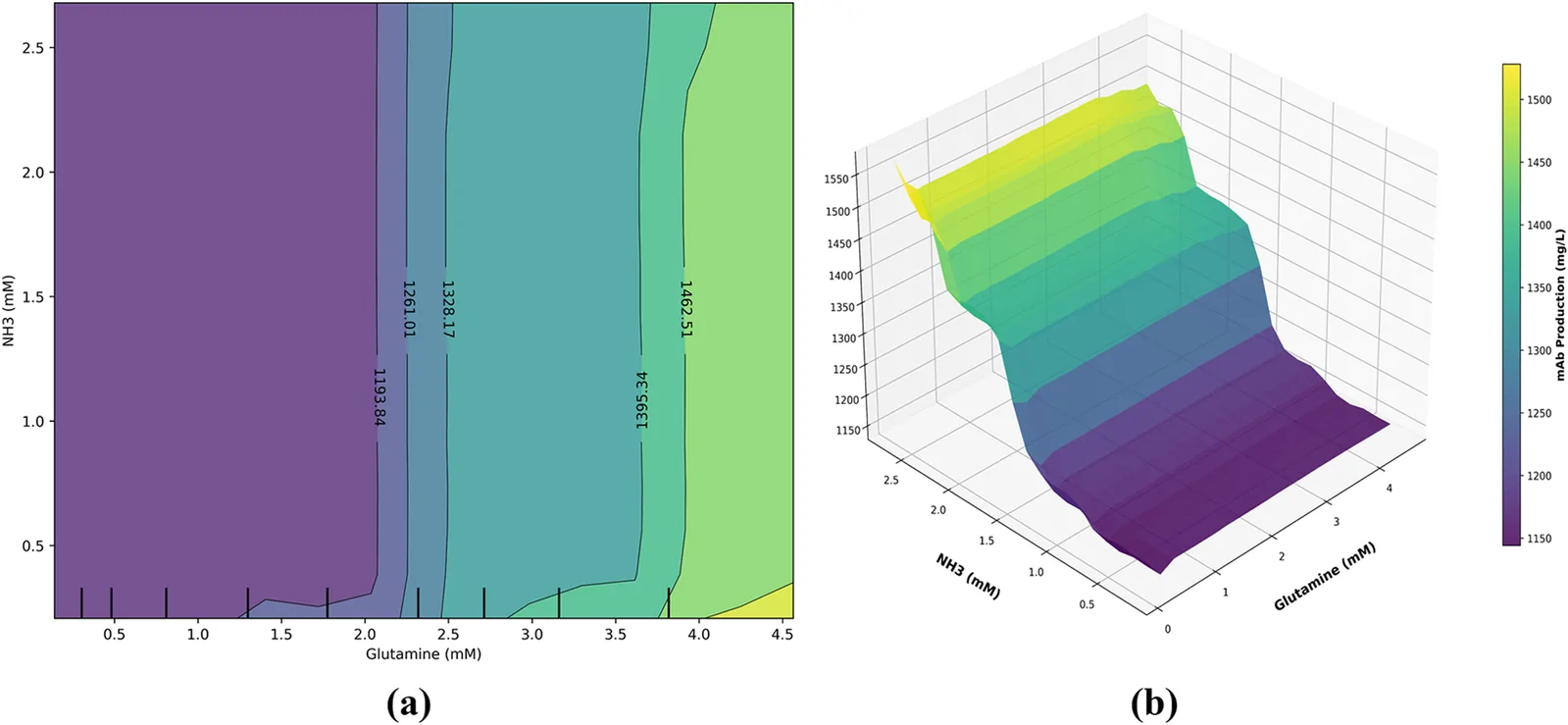
2D-PDP of glutamine and NH_3_ on the predicted mAb titer: (a) 2D contour PDP and (b) 3D surface.

The physicochemical conditions mattered less, but their effects were still clear. Temperature PDPs showed decreasing trends for titer as temperature increased from 35–36 °C, and then increased slightly again up to 37 °C. This aligns with the report that decreasing temperature below 37 °C can reduce acidic charge variants but may increase basic variants or change glycosylation.^22,35^ The 2D-PDP of ECT and temperature (Figure 17) confirmed this trend, with slightly lower temperatures associated with higher predicted titers at any given ECT. In this fed-batch process, pH was tightly controlled around 6.85–7.15, and by using CO_2_ and bicarbonate, the PDP showed only minor fluctuations with slightly higher predicted mAb at lower pH. The related parameter pCO_2_ showed the highest predicted titers at low pCO_2_, followed by a decreasing trend when the pCO_2_ was increased. The 2D-PDP of pH and pCO_2_ (Figure 18) highlighted that the highest predicted mAb titer was at slightly lower pH and low pCO_2_, with titers gradually declining as either pH or pCO_2_ increased. Together, these patterns suggest that high CO_2_ and the associated acid load can reduce growth and productivity, and can change mAb charge variants.^22,34^ The pO_2_ PDP was slightly wavy, but overall showed that the predicted titers was increased at low pO_2_ and decreased as pO_2_ increased. In cell cultures, oxygen consumption is highest during the early growth phase and then declines in later stages as the culture goes through maturity or stationary phase. The small waves in the curve are likely due to interactions with other process variables, such as intermittent feeding and the associated changes in oxygen demand.

**Figure 17:**
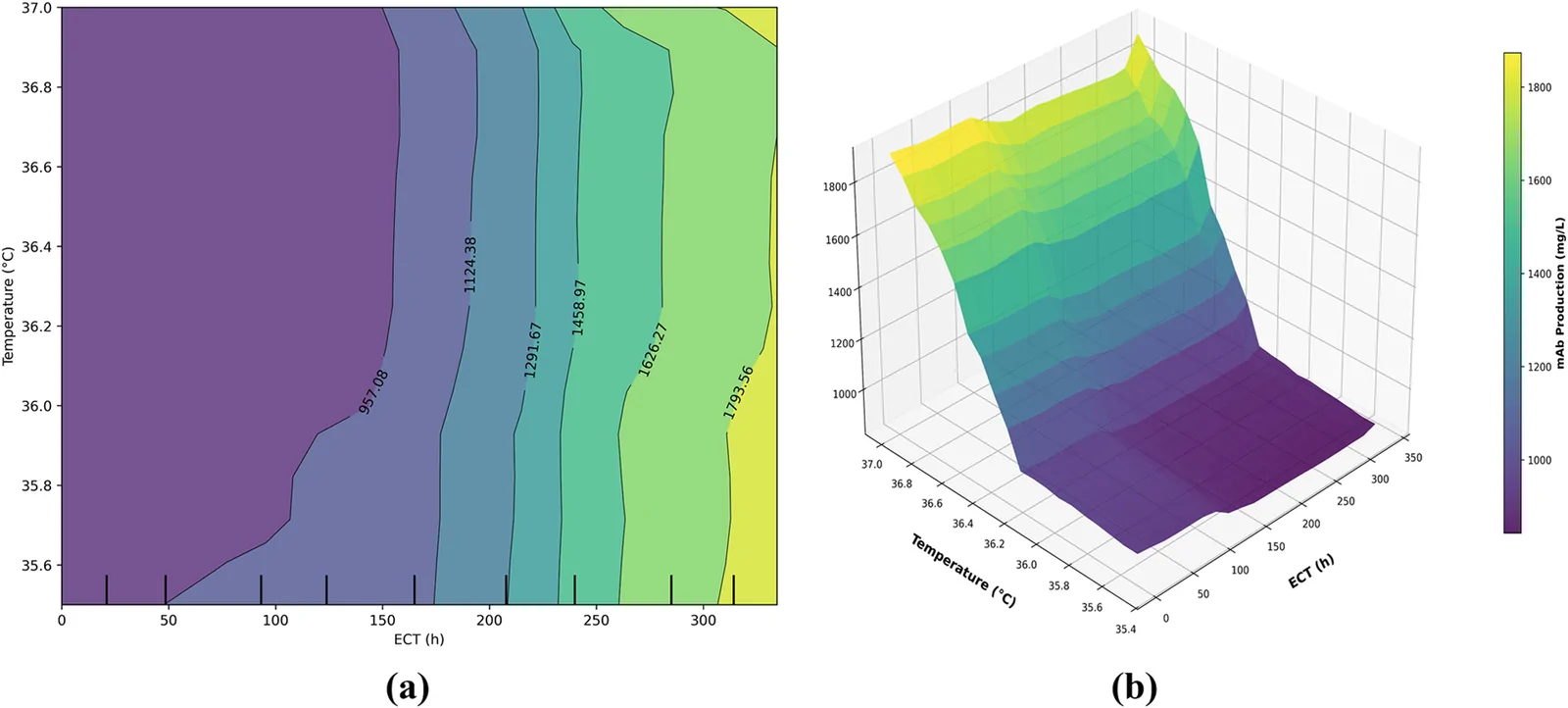
2D-PDP of ECT and temperature on the predicted mAb titer: (a) 2D contour PDP and (b) 3D surface.

**Figure 18:**
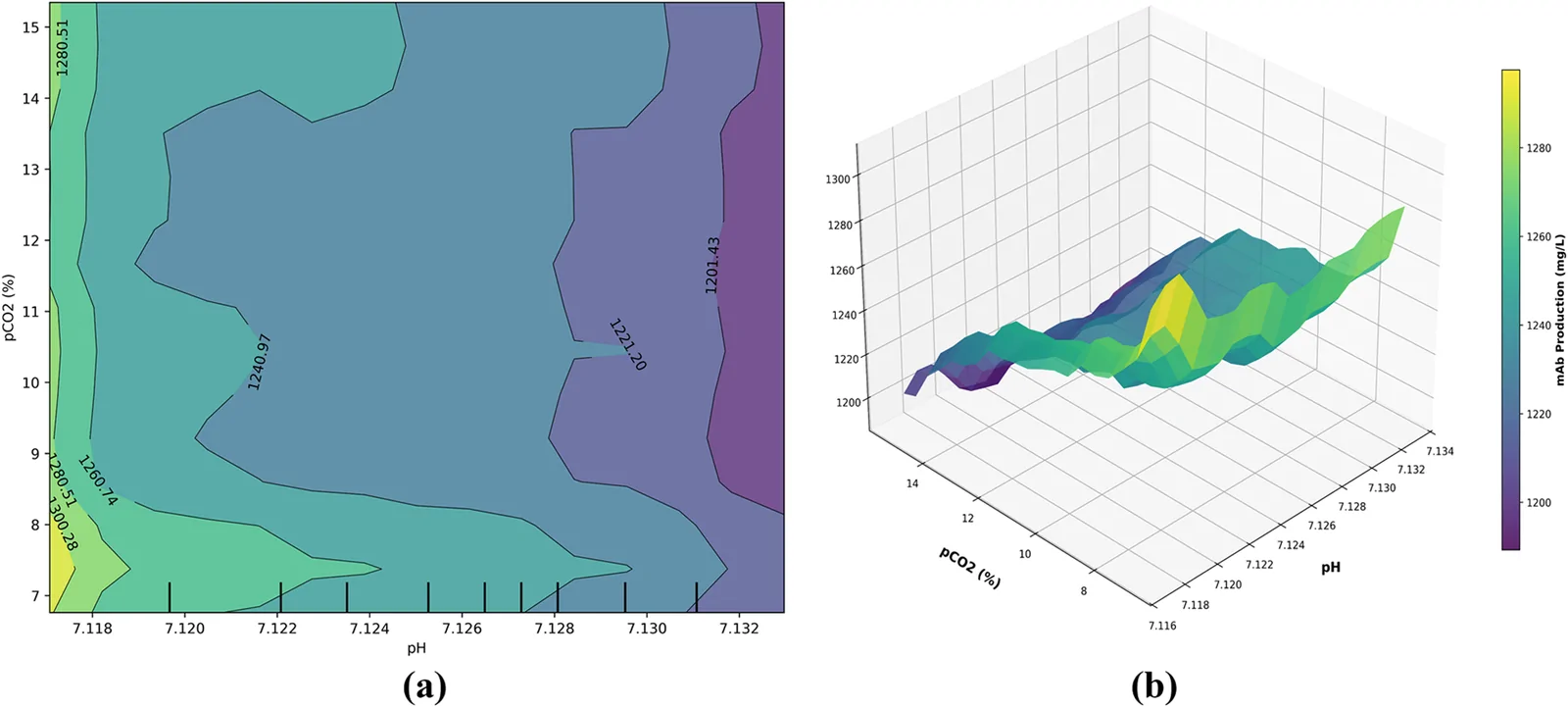
2D-PDP of pH and pCO_2_ on the predicted mAb titer: (a) 2D contour PDP and (b) 3D surface.

Ionic composition and osmolality captured the overall state of the culture medium. The PDP for HCO_3_^–^ showed higher titers at low HCO_3_^–^ and steadily decreased as HCO_3_^–^ increased. This suggested that conditions requiring a lot of HCO_3_^–^ addition are for neutralizing excess CO_2_ and lactate. These are mainly related to stressed cultures under high acid production and lower productivity. The PDP for Na^+^ revealed a rapid increase in titers at low levels of Na^+^ followed by an almost flat level at higher concentrations. This indicated that cells were struggling below a certain ionic strength, but once it reached a standard medium range (around 140 mM), additional sodium did not improve titer; instead sodium would serve as a main contributor to osmotic pressure. The PDP for K^+^ showed increasing titers with higher K^+^. This is probably because K^+^ in the medium only rises in the late stages of the culture, where there are many cells dying and the total titer is already high. Therefore, K^+^ shows that the run was long and productive rather than directly causing higher productivity.^34^ Osmolality PDP showed a gradual increase in predicted titers with increasing osmolality up to 420 mOsm/kg. The higher osmolality is associated with improved performance, which is consistent with the reports that mild hyperosmolality (around 400–420 mOsm/kg) can enhance specific productivity, and very strong hyperosmotic stress (≥470 mOsm/kg) reduces cell growth and final titer.^34^ Selected 2D-PDPs of osmolality with Na^+^ (Figure 19) and HCO_3_^–^ (Figure 20) further support that favorable titers are obtained at moderately elevated osmolality with balanced ionic composition rather than very high salt or buffer additions.

**Figure 19:**
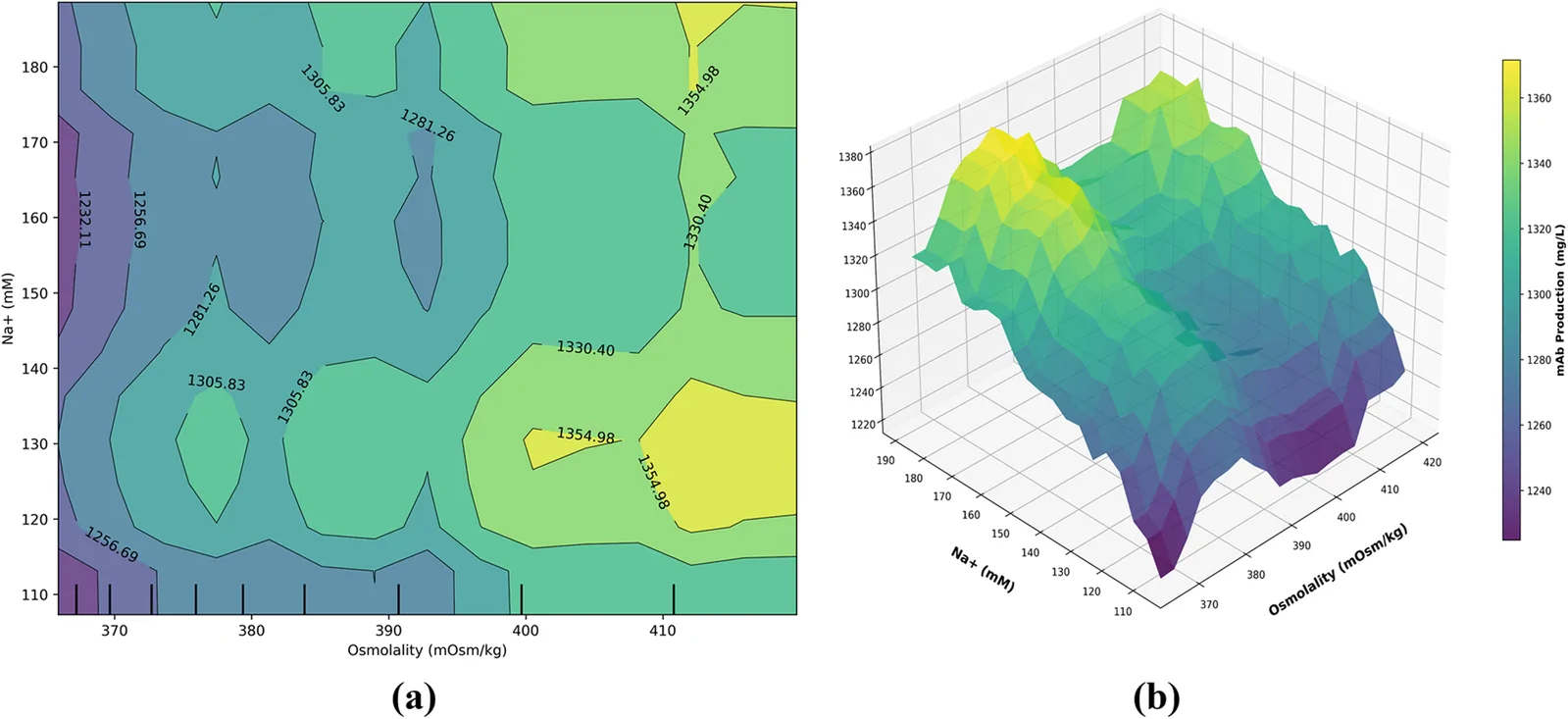
2D-PDP of osmolality and Na^+^ on the predicted mAb titer: (a) 2D contour PDP and (b) 3D surface.

**Figure 20:**
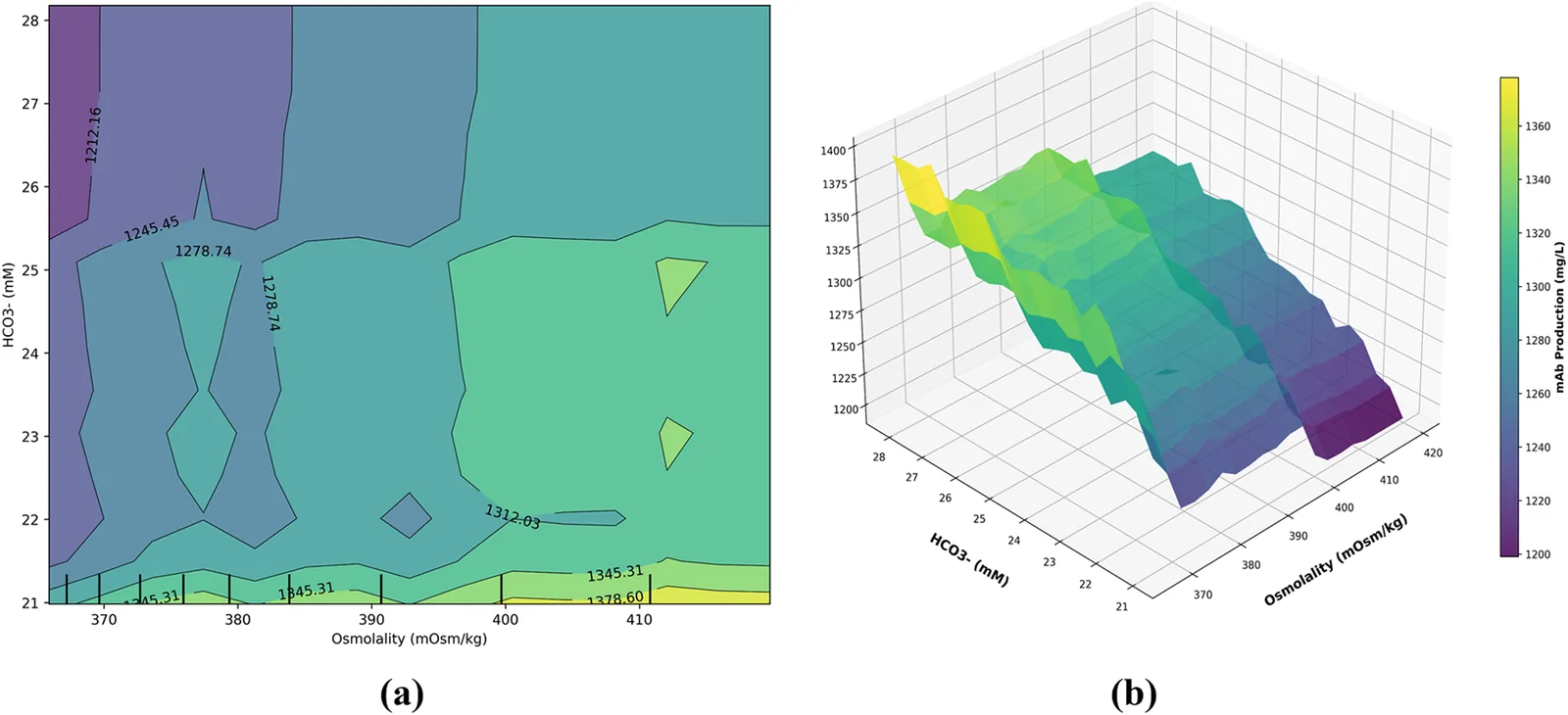
2D-PDP of osmolality and HCO_3_^–^ on the predicted mAb titer: (a) 2D contour PDP and (b) 3D surface.

## 4 Conclusion

This study presents a comprehensive data-driven workflow for a previously published industrial fed-batch dataset of mAb production. The work addressed major challenges that commonly limit the use of historical upstream data, such as the presence of extensive missing values in high-dimensional, irregularly sampled time-series data, lack of systematically benchmarked models, and the difficulty of interpreting complex machine learning models.

First, an imputation pipeline was developed for handling missing data, which was evaluated based on eight supervised ML regressors using systematically engineered temporal features. Among these ML models, LightGBM provided the most accurate imputation results and was selected as the final imputation model. When compared with the previously published dual-hybrid method on the same dataset, the LightGBM-based pipeline maintained all observed measurements exactly. Moreover, it reproduced both the marginal distributions and the multivariate geometry of the data more accurately. This shows that modern ML methods are not only useful for prediction tasks but also for restructuring missing process data in industrial bioreactors.

After imputation, thirty-four extensively benchmarked ML models including linear, nonlinear, neural network, and stacking ensembles were applied for two industrially relevant scenarios. These are (i) pointwise predictions of mAb titer from online process data in real-time (soft sensing) and (ii) early-stage predictions of final titer in advance to support proactive monitoring and control of a process. Among the thirty-four models, nonlinear tree-based ensembles (RF, ExtraTrees, GBR, XGBoost, CatBoost, and LightGBM) performed well over linear and kernel-based models with test set *R* ^2^ values of up to 0.98 for soft sensing and 0.93 for early prediction. This confirms the strong nonlinear relationship between the upstream process variables and the product titer. Stacked ensembles of strong tree-based models gave modest but consistent gains over those of the best single models. Their main benefit was to reduce error metrics and make the cross-validation results more stable rather than to increase the explained variance. These results suggest that compact ensembles with carefully selected base learners are adequate for robust prediction.

Further, explainable AI analyses gave biologically meaningful results for the process variables for controlling mAb production. From PVI and SHAP analyses, the elapsed culture time, total cell density, culture volume, glutamine, glutamate, and osmolality are identified as the most important variables for predicting mAb titer. In contrast, variables such as glucose, pH, NH_3_, and pO_2_ showed lower importance, indicating that these were tightly controlled within a narrow operating range under which they do not become limiting or inhibitory. PDA analysis further revealed nonlinearities, optima, and interaction effects among important variables. More specifically, they showed the dependence of mAb titer on long ECT combined with intermediate TCD, better effects of moderate hyperosmolality, the importance of high glutamine with minimal ammonia accumulation, and the interaction between glucose and lactate in determining the culture efficiency. All of these results show that the models are highly predictive as well as compatible with established CHO cell culture behavior, supporting their use for understanding and monitoring the processes. The ML-based workflow was designed to be transparent and interpretable rather than being used only as a black-box prediction tool. Imputation quality was evaluated using error metrics, distribution metrics, and dimensionality reduction techniques, while the prediction models were interpreted by using PVI, SHAP, and PDA. This combined evaluation and interpretation make the overall workflow more explainable.

This study had some limitations. Although a wide range of tree-based and other ML models were benchmarked, neural networks were not explored systematically. Multilayer perceptrons with multiple hidden layers could not be included in the Bayesian cross-validation search in a stable way because their parameter search space would need to be discrete rather than the continuous hyperparameter space used in this workflow. All analyses were conducted on an industrial CHO fed-batch process under specific operating conditions, so the results may not apply directly to other process modes such as batch or perfusion, even though the general workflow is applicable to any upstream dataset. In the case of imputation, full-time trajectories were not modeled with sequence models such as RNNs or LSTMs. Because of the blockwise missing values, fragmented time series data led to overfitting, so time information was captured through engineered temporal features. Future work will focus on extending this study toward batch-wise dynamic modeling and optimization for improved decision-making in industrial mAb production.

To the best of our knowledge, this is the first study that combines modern ML-based imputations, extensive benchmarking of single and stacked models, and explainable AI of an industrial mAb dataset. This suggested workflow is easy to execute and reusable, and it provides a practical structure for handling missing data and implementing data-driven soft sensors in the upstream development of mAbs. This method can support faster feedback, informed decision-making, reduced analytical workload, and mechanistic insights through interpretable ML. These advantages help to achieve more efficient and robust mAb upstream processes and lead to data-driven bioprocessing strategies toward Industry 4.0.

## Supplementary Material





## Data Availability

The datasets used in this study are derived from the published literature and are provided in the Supporting Information, including both rescaled and machine learning-based imputed data.

## Notes

The dataset used in this study (XLSX)
Additional tables supporting the Results and Discussion sections (PDF)
