# Supplementary material for: Data-Driven Approach for Bioreactor Monitoring and Prediction in Monoclonal Antibody Production
Source: ACS Omega. 2026 Sep 8;11(37):55512–38. doi: 10.1021/acsomega.6c04405 (PMC13613435; doi:10.1021/acsomega.6c04405)
Supplement: Supplementary file 1 [file ao6c04405_si_002.pdf]

SUPPORTING INFORMATION

Data-Driven Approach for Bioreactor Monitoring and Prediction in Monoclonal Antibody Production

Kadeejathul Kubra, Munawar A. Shaik*

Department of Chemical and Petroleum Engineering, College of Engineering, UAE University, Al Ain P.O. Box 15551, UAE.

*Corresponding Author's Email: munawar@uaeu.ac.ae

This supporting material provides additional tables that support the results and discussion sections. Tables S1-S3 report the full training and validation R^2 values for all imputation models, as well as the error- and distribution-based comparisons between the proposed LightGBM-based imputation and the method of Gangadharan et al. Tables S4-S5 summarize the hyperparameter search spaces and optimized settings for all machine learning models considered in Scenarios 1 and 2.

SUPPORTING TABLES

Table S1. Predefined Hyperparameter Settings for the Machine-Learning Models used for Missing-Value Imputation

imputation model	specified hyperparameters
Random Forest (RF)	n_estimators = 200; max_features = sqrt; random_state = 42
Light Gradient Boosting Machine (LightGBM)	n_estimators = 200; learning_rate = 0.05; max_depth = −1; subsample = 0.8; colsample_bytree = 0.8; random_state = 42
Extremely Randomized Trees (ExtraTrees)	n_estimators = 200; max_features = sqrt; bootstrap = True; max_samples = 0.8; random_state = 42
Gradient Boosting (GBR)	n_estimators = 200; learning_rate = 0.05; max_depth = 4; subsample = 0.9; max_features = sqrt; random_state = 42
Extreme Gradient Boosting (XGBoost)	n_estimators = 200; learning_rate = 0.05; max_depth = 4; subsample = 0.8; colsample_bytree = 0.8; random_state = 42
Category Boosting (CatBoost)	iterations = 200; learning_rate = 0.05; depth = 6; l2_leaf_reg = 3; random_strength = 1; bagging_temperature = 1; subsample = 0.8; colsample_bylevel = 0.8; random_state = 42
Support Vector Regression (SVR)	kernel = rbf; C = 1.0; epsilon = 0.1; gamma = scale; max_iter = 10,000
Decision Tree (DT)	max_depth = 10; min_samples_split = 5; min_samples_leaf = 3; random_state = 42

Table S2. Training and Validation R^2 Values for the Imputation of the Missing Values of the Process Variables by the Different Machine Learning Algorithms

variable	training R^2								validation R^2							
	RF	LightGBM	Extra Trees	GBR	XGBoost	CatBoost	SVR	DT	RF	LightGBM	Extra Trees	GBR	XGBoost	CatBoost	SVR	DT
ECT	1.00	1.00	1.00	1.00	1.00	1.00	0.86	1.00	1.00	1.00	1.00	1.00	1.00	1.00	0.89	1.00
CPDL	1.00	1.00	1.00	1.00	1.00	1.00	0.84	1.00	0.99	0.97	0.99	0.99	0.99	0.99	0.83	0.95
EGN	1.00	1.00	1.00	1.00	1.00	0.99	0.99	1.00	0.99	0.99	0.98	0.99	1.00	0.99	0.95	0.99
mAb	1.00	1.00	0.99	1.00	1.00	0.99	-0.05	1.00	0.98	0.98	0.97	0.98	0.98	0.98	-0.14	0.95
TCD	1.00	1.00	1.00	1.00	1.00	1.00	0.74	1.00	0.98	0.99	0.98	0.98	0.99	0.98	0.77	0.97
K^+	1.00	1.00	1.00	1.00	1.00	0.99	0.98	1.00	0.98	0.99	0.98	0.98	0.98	0.98	0.96	0.97

VCD	1.00	1.00	0.99	1.00	1.00	0.99	0.72	1.00	0.97	0.98	0.96	0.97	0.98	0.98	0.68	0.95
Glutamate	0.99	0.98	0.99	0.99	0.99	0.97	0.95	0.97	0.97	0.96	0.96	0.95	0.92	0.96	0.93	0.94
ACV	0.99	1.00	0.98	0.99	1.00	1.00	0.39	1.00	0.96	0.99	0.94	0.97	1.00	1.00	0.33	1.00
ACD	0.99	1.00	0.98	0.99	1.00	1.00	0.97	1.00	0.96	0.99	0.94	0.97	1.00	0.99	0.96	1.00
Na^+	0.99	0.99	0.99	0.99	0.99	0.98	0.58	0.98	0.95	0.95	0.91	0.97	0.98	0.96	0.42	0.95
Glutamine	0.99	1.00	0.98	0.98	0.99	0.97	0.94	0.98	0.92	0.93	0.93	0.94	0.94	0.93	0.89	0.87
HCO_3^-	0.99	1.00	0.98	0.99	1.00	0.98	0.95	0.99	0.92	0.95	0.90	0.94	0.95	0.96	0.92	0.89
pH	0.98	0.99	0.97	0.96	0.98	0.94	-1.33	0.96	0.92	0.94	0.90	0.93	0.92	0.94	-1.61	0.48
pCO_2	0.99	1.00	0.97	0.98	0.99	0.97	0.85	0.98	0.91	0.93	0.87	0.93	0.93	0.94	0.64	0.83
NH_3	0.99	0.99	0.99	1.00	1.00	0.99	0.94	0.98	0.89	0.95	0.87	0.94	0.93	0.92	0.89	0.83
Temperature	0.99	1.00	0.98	0.99	1.00	0.99	0.82	0.98	0.86	0.92	0.81	0.91	0.89	0.91	0.57	0.90
Lactate	0.99	0.99	0.98	0.99	1.00	0.98	0.80	0.98	0.85	0.92	0.82	0.86	0.93	0.92	0.81	0.79
ACC	0.98	0.99	0.97	0.94	0.97	0.92	-1.14	0.95	0.82	0.86	0.81	0.81	0.85	0.83	-1.63	0.75
Osmolality	0.99	0.99	0.97	0.99	1.00	0.98	0.73	0.97	0.82	0.85	0.79	0.83	0.84	0.85	0.59	0.73
Culture Volume	0.95	0.97	0.91	0.93	0.98	0.87	-0.08	0.93	0.67	0.70	0.65	0.75	0.70	0.70	-0.08	0.46
pO_2	0.92	0.96	0.88	0.88	0.94	0.79	0.42	0.87	0.46	0.46	0.41	0.52	0.50	0.45	0.47	0.30
Glucose	0.93	0.97	0.90	0.84	0.93	0.76	0.38	0.84	0.43	0.52	0.38	0.44	0.50	0.43	0.34	0.14

Table S3. Error and Distribution-based Comparison between Raw and Imputed Datasets using the Proposed LightGBM Method and the Method of Gangadharan et al. for each Process Variable

	error metrics				distribution metrics							
variable	LightGBM		Gangadharan et al.		LightGBM				Gangadharan et al.			
	R^2	RMSE	R^2	RMSE	Wasserstein_Dist	JS_Divergence	KS_Statistic	KS_pvalue	Wasserstein_Dist	JS_Divergence	KS_Statistic	KS_pvalue
ACC	1	0	0.9992	0.0048	0.001	0.03	0.0061	1	0.024	0.1215	0.1617	0
ACD	1	0	0.4112	0.1196	0.0045	0.0432	0.0142	0.9959	0.0092	0.0208	0.0121	0.9997
ACV	1	0	0.8964	0.0491	0.0034	0.0149	0.0125	0.9995	0.0058	0.0019	0.0118	0.9998
CPDL	1	0	1	0	0.0362	0.0993	0.0678	0.0018	0.0076	0.0667	0.0241	0.7618
Culture Volume	1	0	1	0	0	0	0	1	0	0	0	1
EGN	1	0	0.8874	0.0493	0.0252	0.0424	0.081	0.0001	0.0296	0.1623	0.1333	0
Glucose	1	0	0.9961	0.007	0.001	0.0057	0.0064	1	0.0017	0.0072	0.0087	1
Glutamate	1	0	1	0	0.0021	0.0136	0.0171	0.9741	0.0027	0.0122	0.032	0.3994
Glutamine	1	0	1	0.0001	0.0131	0.044	0.0345	0.4216	0.0287	0.0625	0.1062	0
HCO_3^-	1	0	1	0	0.0143	0.0516	0.037	0.3097	0.0316	0.0807	0.1153	0
K^+	1	0	1	0	0.0124	0.0313	0.0358	0.2927	0.015	0.0533	0.0395	0.195
Lactate	1	0	1	0	0.006	0.0217	0.0494	0.047	0.0085	0.0051	0.0759	0.0003
mAb	1	0	1	0	0.049	0.0601	0.1327	0	0.0505	0.0554	0.1251	0
Na^+	1	0	1	0	0.003	0.0135	0.0201	0.9053	0.0046	0.0146	0.0338	0.3327
NH_3	1	0	1	0	0.0021	0.0695	0.0163	0.9846	0.0035	0.0111	0.0274	0.6007
Osmolality	1	0	1	0	0.0168	0.1429	0.0993	0	0.0108	0.0465	0.0722	0.0017
pCO_2	1	0	1	0.0008	0.0029	0.0225	0.0147	0.9949	0.0167	0.0053	0.0561	0.0151
pH	1	0	-1272.14	4.7218	0.0044	0.022	0.0188	0.9354	4.615	0.8296	0.9994	0
pO_2	1	0	0.9997	0.0019	0.0067	0.0195	0.0316	0.4338	0.0052	0.0203	0.0266	0.6601
TCD	1	0	0.8515	0.0846	0.0002	0.002	0.0006	1	0.0133	0.1329	0.0308	0.418
Temperature	1	0	1	0	0.0539	0.6157	0.1019	0	0.4427	0	0.2384	0
VCD	1	0	0.8487	0.0846	0.0003	0.0002	0.0011	1	0.0134	0.1335	0.0314	0.3948

Table S4. Hyperparameter Search Spaces and Optimized Settings for all ML Models in Scenario 1

model	search space	best values
Random Forest (RF)	n_estimators: 50–300 (integer); max_depth: 5–30 (integer); min_samples_split: 2–10 (integer); min_samples_leaf: 1–10 (integer); max_features ∈ {sqrt, log2, 0.3, 0.5, 0.7}; bootstrap ∈ {True, False}.	n_estimators = 300; max_depth = 14; min_samples_split = 2; min_samples_leaf = 1; max_features = 0.3; bootstrap = False.
Gradient Boosting (GBR)	n_estimators: 50–300 (integer); learning_rate: 0.01–0.30 (Real, log-uniform); max_depth: 3–10 (integer); min_samples_split: 2–20 (integer); min_samples_leaf: 1–10 (integer); subsample: 0.6–1.0 (Real); max_features ∈ {sqrt, log2, 0.3, 0.5, 0.7}.	n_estimators = 300; learning_rate = 0.0873; max_depth = 7; min_samples_split = 20; min_samples_leaf = 10; subsample = 1.0; max_features = 0.5.
Extreme Gradient Boosting (XGBoost)	n_estimators: 50–300 (integer); learning_rate: 0.01–0.30 (log-uniform); max_depth: 3–10 (integer); min_child_weight: 1–10 (integer); subsample: 0.6–1.0; colsample_bytree: 0.6–1.0; reg_alpha: 0–1.0; reg_lambda: 0–1.0; gamma: 0–0.5.	n_estimators = 271; learning_rate = 0.0704; max_depth = 7; min_child_weight = 7; subsample = 0.6; colsample_bytree = 0.6; reg_alpha = 0.0; reg_lambda = 1.0; gamma = 0.0.
Category Boosting (CatBoost)	iterations: 50–500 (integer); learning_rate: 0.01–0.30 (log-uniform); depth: 3–10 (integer); l2_leaf_reg: 1–10 (Real); random_strength: 0.1–10 (Real); bagging_temperature: 0–1 (Real); border_count: 32–255 (integer).	iterations = 500; learning_rate = 0.0617; depth = 6; l2_leaf_reg = 1.0; random_strength = 0.1; bagging_temperature = 1.0; border_count = 32.
Light Gradient Boosting Machine (LightGBM)	n_estimators: 50–500 (integer); learning_rate: 0.01–0.30 (log-uniform); max_depth: 3–12 (integer); num_leaves: 20–100 (integer); min_child_samples: 5–50 (integer); subsample: 0.6–1.0; colsample_bytree: 0.6–1.0; reg_alpha: 0–1.0; reg_lambda: 0–1.0.	n_estimators = 500; learning_rate = 0.0822; max_depth = 4; num_leaves = 100; min_child_samples = 5; subsample = 0.8870; colsample_bytree = 0.6004; reg_alpha = 0.0; reg_lambda = 0.1648.
Decision Tree (DT)	max_depth: 3–20 (integer); min_samples_split: 2–20 (integer); min_samples_leaf: 1–20 (integer); max_features ∈ {sqrt, log2, 0.3, 0.5, 0.7, 1.0}; max_leaf_nodes: 10–100 (integer); min_impurity_decrease: 0.0–0.1 (Real).	max_depth = 12; min_samples_split = 2; min_samples_leaf = 1; max_features = 0.7; max_leaf_nodes = 46; min_impurity_decrease = 0.0387.
k-Nearest Neighbours (KNN)	n_neighbors: 3–50 (integer); weights ∈ {uniform, distance}; p ∈ {1, 2, 3} (Minkowski metric: 1 = Manhattan, 2 = Euclidean, 3 = general Minkowski); algorithm ∈ {auto, ball_tree, kd_tree, brute}.	n_neighbors = 6; weights = distance; p = 1; algorithm = kd_tree.
Support Vector Regression (SVR)	C: 0.1–1000 (log-uniform); epsilon: 0.01–1.0 (log-uniform); kernel ∈ {linear, rbf, poly}; gamma ∈ {scale, auto}; degree: 2–5 (integer, used for polynomial kernel).	C = 1000.0; epsilon = 1.0; kernel = rbf; gamma = scale.
Extremely Randomized Trees (ExtraTrees)	n_estimators: 50–300 (integer); max_depth: 5–30 (integer); min_samples_split: 2–10 (integer); min_samples_leaf: 1–10 (integer); max_features ∈ {sqrt, log2, 0.3, 0.5, 0.7, 1.0}; bootstrap ∈ {True, False}.	n_estimators = 300; max_depth = 25; min_samples_split = 2; min_samples_leaf = 1; max_features = 1.0; bootstrap = False.
Linear Regression (LR)	Not tuned (standard linear regression).	Not applicable (no hyperparameters).
Ridge Regression (RR)	alpha: 0.001–1000.0 (log-uniform); solver ∈ {auto, svd, cholesky, lsqr, sparse_cg, sag, saga}.	alpha = 0.8802; solver = sparse_cg.

LASSO regression	alpha: 0.001–1000.0 (log-uniform); selection ∈ {cyclic, random}.	alpha = 0.2564; selection = random.
Elastic Net (EN) regression	alpha: 0.001–1000.0 (log-uniform); l1_ratio: 0.01–1.0 (uniform); selection ∈ {cyclic, random}.	alpha = 0.00427; l1_ratio = 0.7502; selection = cyclic.
Multilayer Perceptron (MLP) regressor	hidden_layer_sizes: 50–300 (integer, single hidden layer size); alpha: 1×10^{-6}–1×10^{-2} (log-uniform); learning_rate_init: 1×10^{-4}–1×10^{-1} (log-uniform); batch_size: 32–256 (integer); activation ∈ {relu, tanh, logistic}; solver ∈ {adam, lbfgs}; beta_1: 0.8–0.99; beta_2: 0.9–0.999.	hidden_layer_sizes = 238; alpha = 7.99×10^{-4}; learning_rate_init = 0.0105; batch_size = 102; activation = tanh; solver = adam; beta_1 = 0.9679; beta_2 = 0.9672.
RF+GBR+RR	RF (base learner): n_estimators: 50–300; max_depth: 5–30; min_samples_split: 2–10; min_samples_leaf: 1–10; max_features ∈ {sqrt, log2, 0.3, 0.5, 0.7, 1.0}; bootstrap ∈ {True, False}. GBR (base learner): n_estimators: 50–300; learning_rate: 0.01–0.30 (log-uniform); max_depth: 3–10; min_samples_split: 2–20; min_samples_leaf: 1–10; subsample: 0.6–1.0; max_features ∈ {sqrt, log2, 0.3, 0.5, 0.7}. RR (meta-learner): alpha: 0.001–1000.0 (log-uniform); solver ∈ {auto, svd, cholesky, lsqr, sparse_cg, sag, saga}.	RF: n_estimators = 125; max_depth = 30; min_samples_split = 10; min_samples_leaf = 10; max_features = log2; bootstrap = True.GBR: n_estimators = 300; learning_rate = 0.0251; max_depth = 10; min_samples_split = 15; min_samples_leaf = 10; subsample = 1.0; max_features = 0.3. RR (meta): alpha = 1.41; solver = auto.
RF+XGBoost+RR	RF (base learner): n_estimators: 50–300 (integer); max_depth: 5–30 (integer); min_samples_split: 2–10 (integer) min_samples_leaf: 1–10 (integer); max_features ∈ {sqrt, log2, 0.3, 0.5, 0.7, 1.0}; bootstrap ∈ {True, False}<br>XGBoost (base learner): n_estimators: 50–300 (integer); learning_rate: 0.01–0.30 (log-uniform); max_depth: 3–10 (integer); min_child_weight: 1–10 (integer); subsample: 0.6–1.0; colsample_bytree: 0.6–1.0; reg_alpha: 0–1.0; reg_lambda: 0–1.0; gamma: 0–0.5; RR (meta-learner): alpha: 0.001–1000.0 (log-uniform); solver ∈ {auto, svd, cholesky, lsqr, sparse_cg, sag, saga}	RF: n_estimators = 182; max_depth = 26; min_samples_split = 3; min_samples_leaf = 1; max_features = 0.5; bootstrap = False. XGBoost: n_estimators = 182; learning_rate ≈ 0.0909; max_depth = 5; min_child_weight = 6; subsample ≈ 0.6586; colsample_bytree ≈ 0.8076; reg_alpha = 0.0; reg_lambda ≈ 0.8402; gamma ≈ 0.2482. RR (meta): alpha ≈ 172.97; solver = sag.
RF+CatBoost+RR	RF (base learner): n_estimators: 50–300; max_depth: 5–30; min_samples_split: 2–10; min_samples_leaf: 1–10; max_features ∈ {sqrt, log2, 0.3, 0.5, 0.7, 1.0}; bootstrap ∈ {True, False} CatBoost (base learner): iterations: 50–500 (integer); learning_rate: 0.01–0.30 (log-uniform); depth: 3–10 (integer); l2_leaf_reg: 1–10; random_strength: 0.1–10; bagging_temperature: 0–1; border_count: 32–255 (integer) RR (meta-learner): alpha: 0.001–1000.0 (log-uniform); solver ∈ {auto, svd, cholesky, lsqr, sparse_cg, sag, saga}	RF: n_estimators = 300; max_depth = 18; min_samples_split = 5; min_samples_leaf = 1; max_features = 0.5; bootstrap = False. CatBoost: iterations = 500; learning_rate ≈ 0.30; depth = 5; l2_leaf_reg ≈ 9.5462; random_strength = 0.1; bagging_temperature = 1.0; border_count = 204.<br>RR (meta): alpha ≈ 0.0091; solver = lsqr.
RF+LightGBM+RR	RF (base learner): n_estimators: 50–300; max_depth: 5–30; min_samples_split: 2–10, min_samples_leaf: 1–10; max_features ∈ {sqrt, log2, 0.3, 0.5, 0.7, 1.0}; bootstrap ∈ {True, False}. LightGBM (base learner): n_estimators: 50–500 (integer); learning_rate: 0.01-0.30 (log-uniform); max_depth: 3–12 (integer); num_leaves: 20–100 (integer); min_child_samples: 5–50 (integer); subsample: 0.6–1.0; colsample_bytree: 0.6–1.0; reg_alpha: 0–1.0; reg_lambda: 0–1.0; RR (meta-learner): alpha: 0.001–1000.0 (log-uniform); solver ∈ {auto, svd, cholesky, lsqr, sparse_cg, sag, saga}	RF: n_estimators = 141; max_depth = 30; min_samples_split = 2; min_samples_leaf = 1; max_features = sqrt; bootstrap = True. LightGBM: n_estimators = 500; learning_rate ≈ 0.0379; max_depth = 12; num_leaves = 39; min_child_samples = 5; subsample = 0.6; colsample_bytree = 0.6; reg_alpha ≈ 0.6522; reg_lambda = 1.0. RR (meta): alpha = 0.001; solver = auto.

ExtraTrees+GBR+RR	ExtraTrees (base learner): n_estimators: 50–300 (integer); max_depth: 5–30 (integer); min_samples_split: 2–10 (integer); min_samples_leaf: 1–10 (integer); max_features ∈ {sqrt, log2, 0.3, 0.5, 0.7, 1.0}; bootstrap ∈ {True, False}. GBR (base learner): n_estimators: 50–300 (integer); learning_rate: 0.01–0.30 (log-uniform); max_depth: 3–10 (integer); min_samples_split: 2–20 (integer); min_samples_leaf: 1–10 (integer); subsample: 0.6–1.0; max_features ∈ {sqrt, log2, 0.3, 0.5, 0.7}. RR (meta-learner): alpha: 0.001–1000.0 (log-uniform); solver ∈ {auto, svd, cholesky, lsqr, sparse_cg, sag, saga}	ExtraTrees: n_estimators = 66; max_depth = 27; min_samples_split = 9; min_samples_leaf = 10; max_features = log2; bootstrap = False. GBR: n_estimators = 147; learning_rate ≈ 0.0869; max_depth = 10; min_samples_split = 16; min_samples_leaf = 10; subsample ≈ 0.8429; max_features = 0.5. RR (meta): alpha ≈ 0.00676; solver = cholesky.
ExtraTrees+XGBoost+RR	ExtraTrees (base learner): n_estimators: 50–300 (integer); max_depth: 5–30 (integer); min_samples_split: 2–10 (integer); min_samples_leaf: 1–10 (integer); max_features ∈ {sqrt, log2, 0.3, 0.5, 0.7, 1.0}; bootstrap ∈ {True, False}. XGBoost (base learner): n_estimators: 50–300 (integer); learning_rate: 0.01–0.30 (log-uniform); max_depth: 3–10 (integer); min_child_weight: 1–10 (integer); subsample: 0.6–1.0; colsample_bytree: 0.6–1.0; reg_alpha: 0–1.0; reg_lambda: 0–1.0; gamma: 0–0.5. RR (meta-learner): alpha: 0.001–1000.0 (log-uniform); solver ∈ {auto, svd, cholesky, lsqr, sparse_cg, sag, saga}	ExtraTrees: n_estimators = 240; max_depth = 22; min_samples_split = 9; min_samples_leaf = 9; max_features = sqrt; bootstrap = False. XGBoost: n_estimators = 233; learning_rate ≈ 0.0331; max_depth = 8; min_child_weight = 5; subsample = 0.6; colsample_bytree = 0.6; reg_alpha ≈ 0.333; reg_lambda ≈ 0.225; gamma ≈ 0.2025. RR (meta): alpha ≈ 169.98; solver = cholesky.
ExtraTrees+CatBoost+RR	ExtraTrees (base learner): n_estimators: 50–300; max_depth: 5–30; min_samples_split: 2–10; min_samples_leaf: 1–10; max_features ∈ {sqrt, log2, 0.3, 0.5, 0.7, 1.0}; bootstrap ∈ {True, False}. CatBoost (base learner): iterations: 50–500 (integer); learning_rate: 0.01–0.30 (log-uniform); depth: 3–10 (integer); l2_leaf_reg: 1–10; random_strength: 0.1–10; bagging_temperature: 0–1; border_count: 32–255 (integer). RR (meta-learner): alpha: 0.001–1000.0 (log-uniform); solver ∈ {auto, svd, cholesky, lsqr, sparse_cg, sag, saga}	ExtraTrees: n_estimators = 51; max_depth = 5; min_samples_split = 3; min_samples_leaf = 1; max_features = 0.7; bootstrap = False. CatBoost: iterations = 488; learning_rate ≈ 0.1715; depth = 6; l2_leaf_reg ≈ 6.9543; random_strength ≈ 1.8210; bagging_temperature ≈ 0.0534; border_count = 184. RR (meta): alpha ≈ 31.68; solver = cholesky.
ExtraTrees+LightGBM+RR	ExtraTrees (base learner): n_estimators: 50–300; max_depth: 5–30; min_samples_split: 2–10; min_samples_leaf: 1–10; max_features ∈ {sqrt, log2, 0.3, 0.5, 0.7, 1.0}; bootstrap ∈ {True, False}. LightGBM (base learner): n_estimators: 50–500 (integer); learning_rate: 0.01–0.30 (log-uniform); max_depth: 3–12 (integer); num_leaves: 20–100 (integer); min_child_samples: 5–50 (integer); subsample: 0.6–1.0; colsample_bytree: 0.6–1.0; reg_alpha: 0–1.0; reg_lambda: 0–1.0. RR (meta-learner): alpha: 0.001–1000.0 (log-uniform); solver ∈ {auto, svd, cholesky, lsqr, sparse_cg, sag, saga}	ExtraTrees: n_estimators = 278; max_depth = 6; min_samples_split = 7; min_samples_leaf = 5; max_features = 0.7; bootstrap = False. LightGBM: n_estimators = 500; learning_rate ≈ 0.0610; max_depth = 12; num_leaves = 53; min_child_samples = 11; subsample ≈ 0.9580; colsample_bytree = 0.6; reg_alpha ≈ 0.3099; reg_lambda ≈ 0.3640. RR (meta): alpha ≈ 0.5471; solver = svd.
RF+GBR+XGBoost as meta	RF (base learner): n_estimators: 50–300; max_depth: 5–30; min_samples_split: 2–10; min_samples_leaf: 1–10; max_features ∈ {sqrt, log2, 0.3, 0.5, 0.7, 1.0}; bootstrap ∈ {True, False}. GBR (base learner): n_estimators: 50–300; learning_rate: 0.01–0.30 (log-uniform); max_depth: 3–10; min_samples_split: 2–20; min_samples_leaf: 1–10; subsample: 0.6–1.0; max_features ∈ {sqrt, log2, 0.3, 0.5, 0.7}. XGBoost (meta-learner): n_estimators: 50–300 (integer); learning_rate: 0.01–0.30 (log-uniform); max_depth: 3–10 (integer) min_child_weight: 1–10	RF: n_estimators = 122; max_depth = 17; min_samples_split = 7; min_samples_leaf = 10; max_features = sqrt; bootstrap = False. GBR: n_estimators = 256; learning_rate = 0.01; max_depth = 10; min_samples_split = 2; min_samples_leaf = 1; subsample ≈ 0.8590; max_features = 0.3. XGBoost (meta): n_estimators = 300; learning_rate ≈ 0.0211; max_depth = 3; min_child_weight = 5; subsample ≈

	(integer); subsample: 0.6–1.0; colsample_bytree: 0.6–1.0; reg_alpha: 0–1.0; reg_lambda: 0–1.0; gamma: 0–0.5	0.8784; colsample_bytree = 1.0; reg_alpha ≈ 0.7754; reg_lambda ≈ 0.9241; gamma = 0.5.
GBR+CatBoost+RR	GBR (base learner): n_estimators: 50–300; learning_rate: 0.01–0.30 (log-uniform); max_depth: 3–10; min_samples_split: 2–20; min_samples_leaf: 1–10; subsample: 0.6–1.0; max_features ∈ {sqrt, log2, 0.3, 0.5, 0.7}. CatBoost (base learner): iterations: 50–500 (integer); learning_rate: 0.01–0.30 (log-uniform); depth: 3–10 (integer); l2_leaf_reg: 1–10; random_strength: 0.1–10; bagging_temperature: 0–1; border_count: 32–255 (integer). RR (meta-learner): alpha: 0.001–1000.0 (log-uniform); solver ∈ {auto, svd, cholesky, lsqr, sparse_cg, sag, saga}	GBR: n_estimators = 300; learning_rate ≈ 0.0624; max_depth = 7; min_samples_split = 11; min_samples_leaf = 5; subsample ≈ 0.7021; max_features = 0.5. CatBoost: iterations = 500; learning_rate ≈ 0.30; depth = 3; l2_leaf_reg ≈ 6.8829; random_strength ≈ 1.0339; bagging_temperature = 0.0; border_count = 106. RR (meta): alpha ≈ 0.0168; solver = svd.
GBR+LightGBM+RR	GBR (base learner): n_estimators: 50–300; learning_rate: 0.01–0.30 (log-uniform); max_depth: 3–10; min_samples_split: 2–20; min_samples_leaf: 1–10; subsample: 0.6–1.0; max_features ∈ {sqrt, log2, 0.3, 0.5, 0.7}. LightGBM (base learner): n_estimators: 50–500 (integer); learning_rate: 0.01–0.30 (log-uniform); max_depth: 3–12 (integer); num_leaves: 20–100 (integer) min_child_samples: 5–50 (integer); subsample: 0.6–1.0; colsample_bytree: 0.6–1.0; reg_alpha: 0–1.0; reg_lambda: 0–1.0. RR (meta-learner): alpha: 0.001–1000.0 (log-uniform); solver ∈ {auto, svd, cholesky, lsqr, sparse_cg, sag, saga}	GBR: n_estimators = 300; learning_rate ≈ 0.0246; max_depth = 10; min_samples_split = 20; min_samples_leaf = 6; subsample = 1.0; max_features = 0.3. LightGBM: n_estimators = 500; learning_rate ≈ 0.30; max_depth = 3; num_leaves = 61; min_child_samples = 18; subsample = 1.0; colsample_bytree = 0.6; reg_alpha = 1.0; reg_lambda = 0.0. RR (meta): alpha ≈ 0.00137; solver = auto.
ExtraTrees+GBR+CatBoost+RR	ExtraTrees (base learner): n_estimators: 50–300 (integer); max_depth: 5–30 (integer); min_samples_split: 2–10 (integer); min_samples_leaf: 1–10 (integer); max_features ∈ {sqrt, log2, 0.3, 0.5, 0.7, 1.0}; bootstrap ∈ {True, False}. GBR (base learner): n_estimators: 50–300; learning_rate: 0.01–0.30 (log-uniform); max_depth: 3–10; min_samples_split: 2–20; min_samples_leaf: 1–10; subsample: 0.6–1.0; max_features ∈ {sqrt, log2, 0.3, 0.5, 0.7}. CatBoost (base learner): iterations: 50–500: learning_rate: 0.01–0.30 (log-uniform); depth: 3–10; l2_leaf_reg: 1–10; random_strength: 0.1–10; bagging_temperature: 0–1; border_count: 32–255. RR (meta-learner): alpha: 0.001–1000.0 (log-uniform); solver ∈ {auto, svd, cholesky, lsqr, sparse_cg, sag, saga}	ExtraTrees: n_estimators = 52; max_depth = 30; min_samples_split = 5; min_samples_leaf = 1; max_features = sqrt; bootstrap = True. GBR: n_estimators = 300; learning_rate ≈ 0.3000; max_depth = 9; min_samples_split = 18; min_samples_leaf = 1; subsample ≈ 0.9172; max_features = 0.7. CatBoost: iterations = 488; learning_rate ≈ 0.0719; depth = 7; l2_leaf_reg ≈ 2.515; random_strength = 0.1; bagging_temperature = 1.0; border_count = 196. RR (meta): alpha = 0.001; solver = saga.
RF+GBR+CatBoost+RR	RF (base learner): n_estimators: 50–300; max_depth: 5–30; min_samples_split: 2–10; min_samples_leaf: 1–10; max_features ∈ {sqrt, log2, 0.3, 0.5, 0.7, 1.0}; bootstrap ∈ {True, False}. GBR (base learner): n_estimators: 50–300; learning_rate: 0.01–0.30 (log-uniform); max_depth: 3–10: min_samples_split: 2–20; min_samples_leaf: 1–10; subsample: 0.6–1.0; max_features ∈ {sqrt, log2, 0.3, 0.5, 0.7}. CatBoost (base learner): iterations: 50–500; learning_rate: 0.01–0.30 (log-uniform); depth: 3–10; l2_leaf_reg: 1–10; random_strength: 0.1–10; bagging_temperature: 0–1; border_count: 32–255. RR (meta-learner): alpha: 0.001–1000.0 (log-uniform); solver ∈ {auto, svd, cholesky, lsqr, sparse_cg, sag, saga}	RF: n_estimators = 53; max_depth = 15; min_samples_split = 10; min_samples_leaf = 9; max_features = 0.5; bootstrap = True. GBR: n_estimators = 167; learning_rate ≈ 0.0865; max_depth = 9; min_samples_split = 8; min_samples_leaf = 7; subsample ≈ 0.9261; max_features = 0.5. CatBoost: iterations = 443; learning_rate ≈ 0.0756; depth = 6; l2_leaf_reg ≈ 6.334; random_strength ≈ 6.523; bagging_temperature ≈ 0.6171; border_count = 205. RR (meta): alpha ≈ 0.341; solver = sag.
RF+GBR+XGBoost+RR	RF (base learner): n_estimators: 50–300; max_depth: 5–30; min_samples_split: 2–10; min_samples_leaf: 1–10; max_features ∈ {sqrt, log2, 0.3, 0.5, 0.7, 1.0}; bootstrap ∈ {True, False}. GBR (base learner): n_estimators: 50–300; learning_rate: 0.01–0.30 (log-uniform);	RF: n_estimators = 83; max_depth = 29; min_samples_split = 7; min_samples_leaf = 10; max_features = 0.3; bootstrap = True. GBR: n_estimators = 266; learning_rate ≈ 0.0167; max_depth = 6;

	max_depth: 3–10; min_samples_split: 2–20; min_samples_leaf: 1–10; subsample: 0.6–1.0; max_features ∈ {sqrt, log2, 0.3, 0.5, 0.7}. XGBoost (base learner): n_estimators: 50–300; learning_rate: 0.01–0.30 (log-uniform); max_depth: 3–10; min_child_weight: 1–10; subsample: 0.6–1.0; colsample_bytree: 0.6–1.0; reg_alpha: 0–1.0; reg_lambda: 0–1.0; gamma: 0–0.5. RR (meta-learner): alpha: 0.001–1000.0 (log-uniform), solver ∈ {auto, svd, cholesky, lsqr, sparse_cg, sag, saga}	min_samples_split = 19; min_samples_leaf = 5; subsample ≈ 0.8612; max_features = 0.5. XGBoost: n_estimators = 184; learning_rate ≈ 0.1084; max_depth = 5; min_child_weight = 7; subsample ≈ 0.6851; colsample_bytree ≈ 0.6088; reg_alpha ≈ 0.8597; reg_lambda ≈ 0.9712; gamma ≈ 0.1373. RR (meta): alpha ≈ 0.0167; solver = cholesky.
RF+XGBoost+CatBoost+RR	RF (base learner): n_estimators: 50–300; max_depth: 5–30; min_samples_split: 2–10; min_samples_leaf: 1–10; max_features ∈ {sqrt, log2, 0.3, 0.5, 0.7, 1.0}; bootstrap ∈ {True, False}. XGBoost (base learner): n_estimators: 50–300; learning_rate: 0.01–0.30 (log-uniform); max_depth: 3–10; min_child_weight: 1–10; subsample: 0.6–1.0; colsample_bytree: 0.6–1.0; reg_alpha: 0–1.0; reg_lambda: 0–1.0; gamma: 0–0.5. CatBoost (base learner): iterations: 50–500; learning_rate: 0.01–0.30 (log-uniform); depth: 3–10; l2_leaf_reg: 1–10; random_strength: 0.1–10; bagging_temperature: 0–1; border_count: 32–255. RR (meta-learner); alpha: 0.001–1000.0 (log-uniform) solver ∈ {auto, svd, cholesky, lsqr, sparse_cg, sag, saga}	RF: n_estimators = 269; max_depth = 24; min_samples_split = 8; min_samples_leaf = 3; max_features = 1.0; bootstrap = False. XGBoost: n_estimators = 288; learning_rate ≈ 0.0652; max_depth = 7;min_child_weight = 8; subsample ≈ 0.7427; colsample_bytree = 1.0;reg_alpha = 0.0; reg_lambda ≈ 0.7217; gamma ≈ 0.2783.CatBoost: iterations = 192; learning_rate ≈ 0.3000; depth = 5;l2_leaf_reg ≈ 5.259; random_strength ≈ 1.056; bagging_temperature ≈ 0.2156; border_count = 130. RR (meta): alpha ≈ 0.2411; solver = sag.
ExtraTrees+GBR+LightGBM+RR	ExtraTrees (base learner): n_estimators: 50–300 (integer); max_depth: 5–30; min_samples_split: 2–10; min_samples_leaf: 1–10; max_features ∈ {sqrt, log2, 0.3, 0.5, 0.7, 1.0}; bootstrap ∈ {True, False}. GBR (base learner): n_estimators: 50–300; learning_rate: 0.01–0.30 (log-uniform); max_depth: 3–10; min_samples_split: 2–20; min_samples_leaf: 1–10; subsample: 0.6–1.0; max_features ∈ {sqrt, log2, 0.3, 0.5, 0.7}. LightGBM (base learner): n_estimators: 50–500; learning_rate: 0.01–0.30 (log-uniform); max_depth: 3–12; num_leaves: 20–100; min_child_samples: 5–50; subsample: 0.6–1.0; colsample_bytree: 0.6–1.0; reg_alpha: 0–1.0; reg_lambda: 0–1.0. RR (meta-learner): alpha: 0.001–1000.0 (log-uniform); solver ∈ {auto, svd, cholesky, lsqr, sparse_cg, sag, saga}	ExtraTrees: n_estimators = 66; max_depth = 27; min_samples_split = 9; min_samples_leaf = 10; max_features = log2; bootstrap = False. GBR: n_estimators = 147; learning_rate ≈ 0.08691; max_depth = 10; min_samples_split = 16; min_samples_leaf = 10; subsample ≈ 0.84293; max_features = 0.5. LightGBM: n_estimators = 189; learning_rate ≈ 0.01177; max_depth = 9; num_leaves = 39; min_child_samples = 42; subsample ≈ 0.79029; colsample_bytree ≈ 0.65641; reg_alpha ≈ 0.60368; reg_lambda ≈ 0.24637. RR (meta): alpha ≈ 0.00676; solver = cholesky.
RF+GBR+SVR+MLP+RR	RF (base learner): n_estimators: 50–300; max_depth: 5–30; min_samples_split: 2–10; min_samples_leaf: 1–10; max_features ∈ {sqrt, log2, 0.3, 0.5, 0.7}; bootstrap ∈ {True, False}. GBR (base learner): n_estimators: 50–300; learning_rate: 0.01–0.30 (log-uniform); max_depth: 3–10; min_samples_split: 2–20; min_samples_leaf: 1–10; subsample: 0.6–1.0; max_features ∈ {sqrt, log2, 0.3, 0.5, 0.7}. SVR (base learner): C: 0.1–1000 (log-uniform); epsilon: 0.01–1.0 (log-uniform); kernel ∈ {linear, rbf, poly} gamma ∈ {scale, auto}; degree: 2–5. MLP (base learner): hidden_layer_sizes: 50–300; alpha: 1e−6–1e−2 (log-uniform); learning_rate_init: 1e−4–1e−1 (log-uniform); batch_size: 32–256; activation ∈ {relu, tanh, logistic}; solver ∈ {adam, lbfgs}; beta_1: 0.8–0.99; beta_2: 0.9–0.999. RR (meta-learner): alpha: 0.1–10 (log-uniform)	RF: n_estimators = 260; max_depth = 6; min_samples_split = 6; min_samples_leaf = 4; max_features = 0.5; bootstrap = True. GBR: n_estimators = 239; learning_rate ≈ 0.01794; max_depth = 7; min_samples_split = 4; min_samples_leaf = 6; subsample ≈ 0.94905; max_features = 0.7. SVR: C ≈ 2.6894; epsilon ≈ 0.0716; degree = 3; kernel = rbf; gamma = auto. MLP: hidden_layer_sizes = 57; alpha ≈ 3.53×10^{-5}; learning_rate_init ≈ 0.07836; batch_size = 109; activation = logistic; solver = adam; beta_1 ≈ 0.95913; beta_2 ≈ 0.96043. RR (meta): alpha ≈ 4.215.

GBR+CatBoost+LightGBM+MLP+LR	GBR (base learner): n_estimators: 50–300; learning_rate: 0.01–0.30 (log-uniform); max_depth: 3–10; min_samples_split: 2–20; min_samples_leaf: 1–10; subsample: 0.6–1.0; max_features ∈ {sqrt, log2, 0.3, 0.5, 0.7} CatBoost (base learner): iterations: 50–500; learning_rate: 0.01–0.30 (log-uniform); depth: 3–10; l2_leaf_reg: 1–10; random_strength: 0.1–10; bagging_temperature: 0–1; border_count: 32–255. LightGBM (base learner): n_estimators: 50–500; learning_rate: 0.01–0.30 (log-uniform); max_depth: 3–12; num_leaves: 20–100; min_child_samples: 5–50; subsample: 0.6–1.0; colsample_bytree: 0.6–1.0; reg_alpha: 0–1.0; reg_lambda: 0–1.0. MLP (base learner): hidden_layer_sizes: 50–300; alpha: 1e−6–1e−2 (log-uniform); learning_rate_init: 1e−4–1e−1 (log-uniform); batch_size: 32–256; activation ∈ {relu, tanh, logistic}; solver ∈ {adam, lbfgs}; beta_1: 0.8–0.99; beta_2: 0.9–0.999. LR (meta-learner): No tunable hyperparameters.	GBR: n_estimators = 126; learning_rate ≈ 0.26081; max_depth = 8; min_samples_split = 16; min_samples_leaf = 3; subsample ≈ 0.92085; max_features = log2. CatBoost: iterations = 149; learning_rate ≈ 0.01192; depth = 8; l2_leaf_reg ≈ 8.7561; random_strength ≈ 5.9057; bagging_temperature ≈ 0.9037; border_count = 216. LightGBM: n_estimators = 500; learning_rate ≈ 0.21014; max_depth = 9; num_leaves = 20; min_child_samples = 7; subsample ≈ 0.92952; colsample_bytree ≈ 0.99109; reg_alpha ≈ 0.66679; reg_lambda ≈ 0.53445. MLP: hidden_layer_sizes = 280; alpha = 1×10^{-6}; learning_rate_init ≈ 1.08×10^{-4}; batch_size = 236; activation = relu; solver = lbfgs; beta_1 ≈ 0.93365; beta_2 ≈ 0.95984. LR (meta): No hyperparameters.
RF+GBR+ExtraTrees+CatBoost+RR	RF (base learner): n_estimators: 50–300; max_depth: 5–30; min_samples_split: 2–10; min_samples_leaf: 1–10; max_features ∈ {sqrt, log2, 0.3, 0.5, 0.7}; bootstrap ∈ {True, False}. GBR (base learner): n_estimators: 50–300; learning_rate: 0.01–0.30 (log-uniform); max_depth: 3–10; min_samples_split: 2–20; min_samples_leaf:1–10; ExtraTrees (base learner): n_estimators: 50–300; max_depth: 5–30; min_samples_split: 2–10; min_samples_leaf: 1–10; max_features ∈ {sqrt, log2, 0.3, 0.5, 0.7, 1.0}; bootstrap ∈ {True, False}. CatBoost (base learner): iterations: 50–500; learning_rate: 0.01–0.30 (log-uniform); depth: 3–10; l2_leaf_reg: 1–10; random_strength: 0.1–10; bagging_temperature: 0–1; border_count: 32–255. RR (meta-learner): alpha: 0.001–1000.0 (log-uniform); solver ∈ {auto, svd, cholesky, lsqr, sparse_cg, sag, saga}	RF: n_estimators = 271; max_depth = 15; min_samples_split = 9; min_samples_leaf = 1; max_features = 0.5; bootstrap = True. GBR: n_estimators = 299; learning_rate ≈ 0.10459; max_depth = 8; min_samples_split = 17; min_samples_leaf = 6; subsample ≈ 0.89797; max_features = 0.5. ExtraTrees: n_estimators = 294; max_depth = 26; min_samples_split = 9; min_samples_leaf = 5; max_features = sqrt; bootstrap = True. CatBoost: iterations = 500; learning_rate ≈ 0.18398; depth = 5; l2_leaf_reg ≈ 7.4146; random_strength ≈ 5.5278; bagging_temperature ≈ 0.2064; border_count = 32. RR (meta): alpha ≈ 0.01804; solver = auto.
GBR+XGBoost+CatBoost+LightGBM+LR	GBR (base learner): n_estimators: 50–300; learning_rate: 0.01–0.30 (log-uniform); max_depth: 3–10; min_samples_split: 2–20; min_samples_leaf: 1–10; subsample: 0.6–1.0; max_features ∈ {sqrt, log2, 0.3, 0.5, 0.7}. XGBoost (base learner): n_estimators: 50–300; learning_rate: 0.01–0.30 (log-uniform); max_depth: 3–10; min_child_weight: 1–10; subsample: 0.6–1.0; colsample_bytree: 0.6–1.0; reg_alpha: 0–1.0; reg_lambda: 0–1.0; gamma: 0–0.5. CatBoost (base learner): iterations: 50–500; learning_rate: 0.01–0.30 (log-uniform); depth: 3–10; l2_leaf_reg: 1–10; random_strength: 0.1–10; bagging_temperature: 0–1; border_count: 32–255. LightGBM (base learner): n_estimators: 50–500; learning_rate: 0.01–0.30 (log-uniform); max_depth: 3–12; num_leaves: 20–100; min_child_samples: 5–50; subsample: 0.6–1.0; colsample_bytree: 0.6–1.0; reg_alpha: 0–1.0; reg_lambda: 0–1.0<br>LR (meta-learner): No tunable hyperparameters.	GBR: n_estimators = 235; learning_rate ≈ 0.06534; max_depth = 9; min_samples_split = 8; min_samples_leaf = 1; subsample ≈ 0.68177; max_features = 0.3. XGBoost: n_estimators = 231; learning_rate ≈ 0.02401; max_depth = 3; min_child_weight = 9; subsample ≈ 0.90463; colsample_bytree ≈ 0.77873; reg_alpha ≈ 0.11343; reg_lambda ≈ 0.42576; gamma ≈ 0.21532. CatBoost: iterations = 427; learning_rate ≈ 0.13918; depth = 3; l2_leaf_reg ≈ 5.5471; random_strength ≈ 0.26094; bagging_temperature ≈ 0.13518; border_count = 55. LightGBM: n_estimators = 480; learning_rate ≈ 0.05900; max_depth = 11; num_leaves = 52; min_child_samples = 46; subsample ≈ 0.76944; colsample_bytree ≈ 0.95659; reg_alpha ≈ 0.52011; reg_lambda ≈ 0.50858. LR: No hyperparameters.

Table S5. Hyperparameter Search Spaces and Optimized Settings for all ML Models in Scenario 2

model	search space	best values
Random Forest (RF)	n_estimators: 50–300; max_depth: 5–30; min_samples_split: 2–10; min_samples_leaf: 1–10; max_features ∈ {sqrt, log2, 0.3, 0.5, 0.7}; bootstrap ∈ {True, False}	n_estimators = 300; max_depth = 26; min_samples_split = 4; min_samples_leaf = 1; max_features = 0.7; bootstrap = False
Gradient Boosting (GBR)	n_estimators: 50–300; learning_rate: 0.01–0.30 (log-uniform); max_depth: 3–10; min_samples_split: 2–20; min_samples_leaf: 1–10; subsample: 0.6–1.0; max_features ∈ {sqrt, log2, 0.3, 0.5, 0.7}	n_estimators = 300; learning_rate ≈ 0.0153; max_depth = 10; min_samples_split = 2; min_samples_leaf = 1; subsample = 1.0; max_features = 0.7
Extreme Gradient Boosting (XGBoost)	n_estimators: 50–300; learning_rate: 0.01–0.30 (log-uniform); max_depth: 3–10; min_child_weight: 1–10; subsample: 0.6–1.0; colsample_bytree: 0.6–1.0; reg_alpha: 0–1.0; reg_lambda: 0–1.0; gamma: 0–0.5	n_estimators = 265; learning_rate ≈ 0.0185; max_depth = 10; min_child_weight = 1; subsample = 1.0; colsample_bytree ≈ 0.7575; reg_alpha ≈ 0.5041; reg_lambda = 0.0; gamma = 0.0
CatBoost	iterations: 50–500; learning_rate: 0.01–0.30 (log-uniform); depth: 3–10; l2_leaf_reg: 1–10; random_strength: 0.1–10; bagging_temperature: 0–1; border_count: 32–255	iterations = 440; learning_rate ≈ 0.2991; depth = 8; l2_leaf_reg ≈ 8.39; random_strength ≈ 0.227; bagging_temperature ≈ 0.987; border_count = 209
LightGBM	n_estimators: 50–500; learning_rate: 0.01–0.30 (log-uniform); max_depth: 3–12; num_leaves: 20–100; min_child_samples: 5–50; subsample: 0.6–1.0; colsample_bytree: 0.6–1.0; reg_alpha: 0–1.0; reg_lambda: 0–1.0	n_estimators = 500; learning_rate ≈ 0.0418; max_depth = 8; num_leaves = 20; min_child_samples = 5; subsample = 0.6; colsample_bytree = 0.6; reg_alpha = 1.0; reg_lambda = 0.0
Decision Tree (DT)	max_depth: 3–20; min_samples_split: 2–20; min_samples_leaf: 1–20; max_features ∈ {sqrt, log2, 0.3, 0.5, 0.7, 1.0}; max_leaf_nodes: 10–100; min_impurity_decrease: 0.0–0.1	max_depth = 11; min_samples_split = 15; min_samples_leaf = 1; max_features = 1.0; max_leaf_nodes = 100; min_impurity_decrease = 0.1
k-Nearest Neighbours (KNN)	n_neighbors: 3–50; weights ∈ {uniform, distance}; p ∈ {1, 2, 3}; algorithm ∈ {auto, ball_tree, kd_tree, brute}	n_neighbors = 6; weights = distance; p = 1; algorithm = kd_tree
Support Vector Regression (SVR)	C: 0.1–1000 (log-uniform); epsilon: 0.01–1.0 (log-uniform); kernel ∈ {linear, rbf, poly}; gamma ∈ {scale, auto}; degree: 2–5	C = 1000.0; epsilon ≈ 0.0666; kernel = rbf; gamma = scale.
ExtraTrees	n_estimators: 50–300; max_depth: 5–30; min_samples_split: 2–10; min_samples_leaf: 1–10; max_features ∈ {sqrt, log2, 0.3, 0.5, 0.7, 1.0}; bootstrap ∈ {True, False}	n_estimators = 300; max_depth = 18; min_samples_split = 2; min_samples_leaf = 1; max_features = 1.0; bootstrap = False
Linear Regression (LR)	Not tuned (standard linear regression).	Not applicable (no hyperparameters)
Ridge Regression (RR)	alpha: 0.001–1000 (log-uniform); solver ∈ {auto, svd, cholesky, lsqr, sparse_cg, sag, saga}	alpha ≈ 156.44; solver = svd
LASSO regression	alpha: 0.001–1000 (log-uniform); selection ∈ {cyclic, random}	alpha ≈ 45.48; selection = random

Elastic Net (EN) regression	alpha: 0.001–1000 (log-uniform); l1_ratio: 0.01–1.0; selection ∈ {cyclic, random}	alpha ≈ 2.02; l1_ratio ≈ 0.82; selection = cyclic
Multilayer Perceptron (MLP) regressor	hidden_layer_sizes: 50–300; alpha: 1×10^{-6}–1×10^{-2} (log-uniform); learning_rate_init: 1×10^{-4}–1×10^{-1} (log-uniform); batch_size: 32–256; activation ∈ {relu, tanh, logistic}; solver ∈ {adam, lbfgs}; beta_1: 0.8–0.99; beta_2: 0.9–0.999	hidden_layer_sizes = 81; alpha = 1×10^{-6}; learning_rate_init ≈ 0.0116; batch_size = 256; activation = logistic; solver = lbfgs; beta_1 = 0.99; beta_2 = 0.90
RF+GBR+RR	RF (base learner): n_estimators: 50–300; max_depth: 5–30; min_samples_split: 2–10; min_samples_leaf: 1–10; max_features ∈ {sqrt, log2, 0.3, 0.5, 0.7, 1.0}; bootstrap ∈ {True, False}. GBR (base learner): n_estimators: 50–300; learning_rate: 0.01–0.30 (log-uniform); max_depth: 3–10; min_samples_split: 2–20; min_samples_leaf: 1–10; subsample: 0.6–1.0; max_features ∈ {sqrt, log2, 0.3, 0.5, 0.7}. RR (meta): alpha: 0.001–1000 (log-uniform); solver ∈ {auto, svd, cholesky, lsqr, sparse_cg, sag, saga}	RF: n_estimators = 185; max_depth = 23; min_samples_split = 5; min_samples_leaf = 1; max_features = 0.7; bootstrap = False. GBR: n_estimators = 59; learning_rate ≈ 0.0478; max_depth = 4; min_samples_split = 2; min_samples_leaf = 1; subsample = 0.6; max_features = 0.7. RR (meta): alpha = 1000.0; solver = auto
RF+XGBoost+RR	RF (base learner): n_estimators: 50–300; max_depth: 5–30; min_samples_split: 2–10; min_samples_leaf: 1–10; max_features ∈ {sqrt, log2, 0.3, 0.5, 0.7, 1.0}; bootstrap ∈ {True, False}. XGBoost (base learner): n_estimators: 50–500; learning_rate: 0.01–0.30 (log-uniform); max_depth: 3–10; min_child_weight: 1–10; subsample: 0.6–1.0; colsample_bytree: 0.6–1.0; reg_alpha: 0–1.0; reg_lambda: 0–1.0; gamma: 0–0.5. RR (meta): alpha: 0.001–1000 (log-uniform); solver ∈ {auto, svd, cholesky, lsqr, sparse_cg, sag, saga}	RF: n_estimators = 241; max_depth = 10; min_samples_split = 10; min_samples_leaf = 6; max_features = 0.3; bootstrap = False. XGBoost: n_estimators = 327; learning_rate ≈ 0.0116; max_depth = 10; min_child_weight = 3; subsample ≈ 0.912; colsample_bytree = 0.6; reg_alpha ≈ 0.965; reg_lambda ≈ 0.165; gamma ≈ 0.191. RR (meta): alpha ≈ 13.85; solver = cholesky
RF+CatBoost+RR	RF (base learner): n_estimators: 50–300; max_depth: 5–30; min_samples_split: 2–10; min_samples_leaf: 1–10; max_features ∈ {sqrt, log2, 0.3, 0.5, 0.7, 1.0}; bootstrap ∈ {True, False}. CatBoost (base learner): iterations: 50–500; learning_rate: 0.01–0.30 (log-uniform); depth: 3–10; l2_leaf_reg: 1–10; random_strength: 0.1–10; bagging_temperature: 0–1; border_count: 32–255. RR (meta): alpha: 0.001–1000 (log-uniform); solver ∈ {auto, svd, cholesky, lsqr, sparse_cg, sag, saga}	RF: n_estimators = 300; max_depth = 24; min_samples_split = 5; min_samples_leaf = 1; max_features = 0.7; bootstrap = False. CatBoost: iterations = 500; learning_rate ≈ 0.0527; depth = 4; l2_leaf_reg ≈ 5.07; random_strength ≈ 9.49; bagging_temperature ≈ 0.340; border_count = 132. RR (meta): alpha ≈ 22.32; solver = auto
RF+LightGBM+RR	RF (base learner): n_estimators: 50–300; max_depth: 5–30; min_samples_split: 2–10; min_samples_leaf: 1–10; max_features ∈ {sqrt, log2, 0.3, 0.5, 0.7, 1.0}; bootstrap ∈ {True, False}. LightGBM (base learner): n_estimators: 50–500; learning_rate: 0.01–0.30 (log-uniform); max_depth: 3–12; num_leaves: 20–100; min_child_samples: 5–50; subsample: 0.6–1.0; colsample_bytree: 0.6–1.0; reg_alpha: 0–1.0; reg_lambda: 0–1.0. RR (meta): alpha: 0.001–1000 (log-uniform); solver ∈ {auto, svd, cholesky, lsqr, sparse_cg, sag, saga}	RF: n_estimators = 50; max_depth = 19; min_samples_split = 4; min_samples_leaf = 1; max_features = 0.7; bootstrap = False. LightGBM: n_estimators = 50; learning_rate = 0.01; max_depth = 3; num_leaves = 37; min_child_samples = 5; subsample ≈ 0.972; colsample_bytree ≈ 0.653; reg_alpha = 0.0; reg_lambda ≈ 0.424. RR (meta): alpha ≈ 0.0709; solver = auto
ExtraTrees+GBR+RR	ExtraTrees (base learner): n_estimators: 50–300; max_depth: 5–30; min_samples_split: 2–10; min_samples_leaf: 1–10; max_features ∈ {sqrt, log2, 0.3, 0.5, 0.7, 1.0}; bootstrap ∈ {True, False}. GBR (base	ExtraTrees: n_estimators = 160; max_depth = 14; min_samples_split = 8; min_samples_leaf = 10; max_features = sqrt; bootstrap = False. GBR: n_estimators =

	learner): n_estimators: 50–300; learning_rate: 0.01–0.30 (log-uniform); max_depth: 3–10; min_samples_split: 2–20; min_samples_leaf: 1–10; subsample: 0.6–1.0; max_features ∈ {sqrt, log2, 0.3, 0.5, 0.7}. RR (meta): alpha: 0.001–1000 (log-uniform); solver ∈ {auto, svd, cholesky, lsqr, sparse_cg, sag, saga}	299; learning_rate ≈ 0.0152; max_depth = 9; min_samples_split = 12; min_samples_leaf = 1; subsample ≈ 0.947; max_features = 0.7. RR (meta): alpha ≈ 0.00163; solver = cholesky
ExtraTrees+XGBoost+RR	ExtraTrees (base learner): n_estimators 50–300; max_depth 5–30; min_samples_split 2–10; min_samples_leaf 1–10; max_features ∈ {sqrt, log2, 0.3, 0.5, 0.7, 1.0}; bootstrap ∈ {True, False}. XGBoost (base learner): n_estimators 50–300; learning_rate 0.01–0.30 (log-uniform); max_depth 3–10; min_child_weight 1–10; subsample 0.6–1.0; colsample_bytree 0.6–1.0; reg_alpha 0–1.0; reg_lambda 0–1.0; gamma 0–0.5. RR (meta): alpha 0.001–1000 (log-uniform); solver ∈ {auto, svd, cholesky, lsqr, sparse_cg, sag, saga}.	ExtraTrees: n_estimators = 50; max_depth = 30; min_samples_split = 2; min_samples_leaf = 1; max_features = sqrt; bootstrap = False. XGBoost: n_estimators = 300; learning_rate ≈ 0.0101; max_depth = 10; min_child_weight = 1; subsample ≈ 0.852; colsample_bytree ≈ 0.706; reg_alpha ≈ 0.357; reg_lambda = 0; gamma = 0.5. RR (meta): alpha = 1000; solver = auto.
ExtraTrees+CatBoost+RR	ExtraTrees (base learner): n_estimators 50–300; max_depth 5–30; min_samples_split 2–10; min_samples_leaf 1–10; max_features ∈ {sqrt, log2, 0.3, 0.5, 0.7, 1.0}; bootstrap ∈ {True, False}. CatBoost (base learner): iterations 50–500; learning_rate 0.01–0.30 (log-uniform); depth 3–10; l2_leaf_reg 1–10; random_strength 0.1–10; bagging_temperature 0–1; border_count 32–255. RR (meta): alpha 0.001–1000 (log-uniform); solver ∈ {auto, svd, cholesky, lsqr, sparse_cg, sag, saga}.	ExtraTrees: n_estimators = 236; max_depth = 15; min_samples_split = 5; min_samples_leaf = 10; max_features = 0.7; bootstrap = True. CatBoost: iterations = 500; learning_rate ≈ 0.0952; depth = 7; l2_leaf_reg ≈ 1.45; random_strength = 0.1; bagging_temperature ≈ 0.428; border_count = 197. RR (meta): alpha ≈ 0.135.
ExtraTrees+LightGBM+RR	ExtraTrees (base learner): n_estimators 50–300; max_depth 5–30; min_samples_split 2–10; min_samples_leaf 1–10; max_features ∈ {sqrt, log2, 0.3, 0.5, 0.7, 1.0}; bootstrap ∈ {True, False}. LightGBM (base learner): n_estimators 50–500; learning_rate 0.01–0.30; max_depth 3–12; num_leaves 20–100; min_child_samples 5–50; subsample 0.6–1.0; colsample_bytree 0.6–1.0; reg_alpha 0–1.0; reg_lambda 0–1.0. RR (meta): alpha 0.001–1000 (log-uniform); solver ∈ {auto, svd, cholesky, lsqr, sparse_cg, sag, saga}.	ExtraTrees: n_estimators = 50; max_depth = 30; min_samples_split = 9; min_samples_leaf = 5; max_features = 0.3; bootstrap = False. LightGBM: n_estimators = 499; learning_rate ≈ 0.0705; max_depth = 9; num_leaves = 82; min_child_samples = 5; subsample = 1.0; colsample_bytree = 0.6; reg_alpha ≈ 0.420; reg_lambda ≈ 0.648. RR (meta): alpha = 0.001.
RF+GBR+XGBoost as meta	RF (base learner): n_estimators 50–300; max_depth 5–30; min_samples_split 2–10; min_samples_leaf 1–10; max_features ∈ {sqrt, log2, 0.3, 0.5, 0.7, 1.0}; bootstrap ∈ {True, False}. GBR (base learner): n_estimators 50–300; learning_rate 0.01–0.30; max_depth 3–10; min_samples_split 2–20; min_samples_leaf 1–10; subsample 0.6–1.0; max_features ∈ {sqrt, log2, 0.3, 0.5, 0.7}. XGBoost (meta): n_estimators 50–300; learning_rate 0.01–0.30; max_depth 3–10; min_child_weight 1–10; subsample 0.6–1.0; colsample_bytree 0.6–1.0; reg_alpha 0–1.0; reg_lambda 0–1.0; gamma 0–0.5.	RF: n_estimators = 180; max_depth = 26; min_samples_split = 6; min_samples_leaf = 1; max_features = 0.5; bootstrap = False. GBR: n_estimators = 273; learning_rate ≈ 0.0167; max_depth = 6; min_samples_split = 20; min_samples_leaf = 9; subsample ≈ 0.938; max_features = 0.7. XGBoost (meta): n_estimators = 225; learning_rate ≈ 0.0139; max_depth = 8; min_child_weight = 10; subsample = 1.0; colsample_bytree = 1.0; reg_alpha ≈ 0.872; reg_lambda = 1.0; gamma = 0.

GBR+CatBoost+RR	GBR (base learner): n_estimators 50–300; learning_rate 0.01–0.30; max_depth 3–10; min_samples_split 2–20; min_samples_leaf 1–10; subsample 0.6–1.0; max_features ∈ {sqrt, log2, 0.3, 0.5, 0.7}. CatBoost (base learner): iterations 50–500; learning_rate 0.01–0.30; depth 3–10; l2_leaf_reg 1–10; random_strength 0.1–10; bagging_temperature 0–1; border_count 32–255. RR (meta): alpha 0.001–1000 (log-uniform); solver ∈ {auto, svd, cholesky, lsqr, sparse_cg, sag, saga}.	GBR: n_estimators = 199; learning_rate = 0.01; max_depth = 10; min_samples_split = 7; min_samples_leaf = 3; subsample ≈ 0.891; max_features = log2. CatBoost: iterations = 471; learning_rate ≈ 0.0845; depth = 7; l2_leaf_reg ≈ 6.29; random_strength = 0.1; bagging_temperature ≈ 0.200; border_count = 255. RR (meta): alpha ≈ 0.0607.
GBR+LightGBM+RR	GBR (base learner): n_estimators 50–300; learning_rate 0.01–0.30; max_depth 3–10; min_samples_split 2–20; min_samples_leaf 1–10; subsample 0.6–1.0; max_features ∈ {sqrt, log2, 0.3, 0.5, 0.7}. LightGBM (base learner): n_estimators 50–500; learning_rate 0.01–0.30; max_depth 3–12; num_leaves 20–100; min_child_samples 5–50; subsample 0.6–1.0; colsample_bytree 0.6–1.0; reg_alpha 0–1.0; reg_lambda 0–1.0. RR (meta): alpha 0.001–1000.	GBR: n_estimators = 300; learning_rate ≈ 0.0119; max_depth = 9; min_samples_split = 14; min_samples_leaf = 1; subsample = 1.0; max_features = 0.7. LightGBM: n_estimators = 441; learning_rate ≈ 0.151; max_depth = 6; num_leaves = 30; min_child_samples = 5; subsample ≈ 0.896; colsample_bytree = 0.6; reg_alpha ≈ 0.860; reg_lambda ≈ 0.932. RR: alpha = 1000.
ExtraTrees+GBR+CatBoost+RR	ExtraTrees (base learner): n_estimators 50–300; max_depth 5–30; min_samples_split 2–10; min_samples_leaf 1–10; max_features ∈ {sqrt, log2, 0.3, 0.5, 0.7, 1.0}; bootstrap ∈ {True, False}. GBR (base learner): n_estimators 50–500; learning_rate 0.01–0.30; max_depth 3–10; min_samples_split 2–20; min_samples_leaf 1–10; subsample 0.6–1.0; max_features ∈ {sqrt, log2, 0.3, 0.5, 0.7}. CatBoost (base learner): iterations 50–500; learning_rate 0.01–0.30; depth 3–10; l2_leaf_reg 1–10; random_strength 0.1–10; bagging_temperature 0–1; border_count 32–255. RR (meta): alpha 0.001–1000.	ExtraTrees: n_estimators = 213; max_depth = 6; min_samples_split = 5; min_samples_leaf = 1; max_features = log2; bootstrap = True. GBR: n_estimators = 394; learning_rate = 0.01; max_depth = 10; min_samples_split = 19; min_samples_leaf = 1; subsample = 1.0; max_features = 0.7. CatBoost: iterations = 141; learning_rate ≈ 0.0186; depth = 6; l2_leaf_reg ≈ 3.81; random_strength ≈ 8.74; bagging_temperature ≈ 0.063; border_count = 129. RR: alpha ≈ 6.72.
RF+GBR+CatBoost+RR	RF (base learner): n_estimators 50–300; max_depth 5–30; min_samples_split 2–10; min_samples_leaf 1–10; max_features ∈ {sqrt, log2, 0.3, 0.5, 0.7, 1.0}; bootstrap ∈ {True, False}. GBR (base learner): n_estimators 50–500; learning_rate 0.01–0.30; max_depth 3–10; min_samples_split 2–20; min_samples_leaf 1–10; subsample 0.6–1.0; max_features ∈ {sqrt, log2, 0.3, 0.5, 0.7}. CatBoost (base learner): iterations 50–500; learning_rate 0.01–0.30; depth 3–10; l2_leaf_reg 1–10; random_strength 0.1–10; bagging_temperature 0–1; border_count 32–255. RR (meta): alpha 0.001–1000.	RF: n_estimators = 290; max_depth = 29; min_samples_split = 4; min_samples_leaf = 1; max_features = 0.7; bootstrap = False. GBR: n_estimators = 500; learning_rate ≈ 0.0209; max_depth = 9; min_samples_split = 11; min_samples_leaf = 3; subsample ≈ 0.800; max_features = 0.7. CatBoost: iterations = 369; learning_rate ≈ 0.0522; depth = 8; l2_leaf_reg ≈ 1.34; random_strength ≈ 9.26; bagging_temperature ≈ 0.546; border_count = 255. RR: alpha ≈ 5.72.
RF+GBR+XGBoost+RR	RF (base learner): n_estimators 50–300; max_depth 5–30; min_samples_split 2–10; min_samples_leaf 1–10; max_features ∈ {sqrt, log2, 0.3, 0.5, 0.7, 1.0}; bootstrap ∈ {True, False}. GBR (base learner): n_estimators 50–500; learning_rate 0.01–0.30; max_depth 3–10; min_samples_split 2–20; min_samples_leaf 1–10; subsample 0.6–1.0; max_features ∈ {sqrt, log2, 0.3, 0.5, 0.7}. XGBoost (base learner): n_estimators 50–500; learning_rate 0.01–0.30; max_depth 3–10; min_child_weight 1–10; subsample 0.6–1.0; colsample_bytree 0.6–1.0; reg_alpha 0–1.0; reg_lambda 0–1.0; gamma 0–0.5. RR (meta): alpha 0.001–1000.	RF: n_estimators = 274; max_depth = 10; min_samples_split = 8; min_samples_leaf = 4; max_features = 0.7; bootstrap = True. GBR: n_estimators = 220; learning_rate ≈ 0.0378; max_depth = 10; min_samples_split = 8; min_samples_leaf = 3; subsample ≈ 0.920; max_features = 0.7. XGBoost: n_estimators = 343; learning_rate ≈ 0.0103; max_depth = 5; min_child_weight = 8; subsample ≈ 0.756; colsample_bytree ≈ 0.828; reg_alpha ≈ 0.445; reg_lambda ≈ 0.170; gamma ≈ 0.483. RR: alpha ≈ 0.032.

RF+XGBoost+CatBoost+RR	RF (base learner): n_estimators 50–300; max_depth 5–30; min_samples_split 2–10; min_samples_leaf 1–10; max_features ∈ {sqrt, log2, 0.3, 0.5, 0.7, 1.0}; bootstrap ∈ {True, False}. XGBoost (base learner): n_estimators 50–300; learning_rate 0.01–0.30; max_depth 3–10; min_child_weight 1–10; subsample 0.6–1.0; colsample_bytree 0.6–1.0; reg_alpha 0–1.0; reg_lambda 0–1.0; gamma 0–0.5. CatBoost (base learner): iterations 50–500; learning_rate 0.01–0.30; depth 3–10; l2_leaf_reg 1–10; random_strength 0.1–10; bagging_temperature 0–1; border_count 32–255. RR (meta): alpha 0.001–1000.	RF: n_estimators = 214; max_depth = 27; min_samples_split = 2; min_samples_leaf = 1; max_features = 0.7; bootstrap = False. XGBoost: n_estimators = 93; learning_rate ≈ 0.123; max_depth = 10; min_child_weight = 1; subsample = 1.0; colsample_bytree ≈ 0.960; reg_alpha = 0; reg_lambda ≈ 0.525; gamma ≈ 0.458. CatBoost: iterations = 50; learning_rate = 0.30; depth = 10; l2_leaf_reg ≈ 9.61; random_strength = 0.1; bagging_temperature ≈ 0.514; border_count = 255. RR: alpha = 0.001.
ExtraTrees+GBR+LightGBM+RR	ExtraTrees (base learner): n_estimators 50–300; max_depth 5–30; min_samples_split 2–10; min_samples_leaf 1–10; max_features ∈ {sqrt, log2, 0.3, 0.5, 0.7, 1.0}; bootstrap ∈ {True, False}. GBR (base learner): n_estimators 50–500; learning_rate 0.01–0.30; max_depth 3–10; min_samples_split 2–20; min_samples_leaf 1–10; subsample 0.6–1.0; max_features ∈ {sqrt, log2, 0.3, 0.5, 0.7}. LightGBM (base learner): n_estimators 50–500; learning_rate 0.01–0.30; max_depth 3–12; num_leaves 20–100; min_child_samples 5–50; subsample 0.6–1.0; colsample_bytree 0.6–1.0; reg_alpha 0–1.0; reg_lambda 0–1.0. RR (meta): alpha 0.001–1000.	ExtraTrees: n_estimators = 75; max_depth = 19; min_samples_split = 10; min_samples_leaf = 4; max_features = 0.7; bootstrap = True. GBR: n_estimators = 132; learning_rate ≈ 0.0511; max_depth = 7; min_samples_split = 5; min_samples_leaf = 1; subsample = 1.0; max_features = 0.7. LightGBM: n_estimators = 58; learning_rate = 0.01; max_depth = 10; num_leaves = 56; min_child_samples = 50; subsample = 0.6; colsample_bytree = 1.0; reg_alpha ≈ 0.392; reg_lambda ≈ 0.861. RR: alpha ≈ 1.25.
RF+GBR+SVR+MLP+RR	RF (base learner): n_estimators 50–300; max_depth 5–30; min_samples_split 2–10; min_samples_leaf 1–10; max_features ∈ {sqrt, log2, 0.3, 0.5, 0.7}; bootstrap ∈ {True, False}. GBR (base learner): n_estimators 50–500; learning_rate 0.01–0.30; max_depth 3–10; min_samples_split 2–20; min_samples_leaf 1–10; subsample 0.6–1.0; max_features ∈ {sqrt, log2, 0.3, 0.5, 0.7}. SVR (base learner): C 0.1–1000; epsilon 0.01–1.0; kernel ∈ {linear, rbf, poly}; gamma ∈ {scale, auto}; degree 2–5. MLP (base learner): hidden_layer_sizes 50–300; alpha 1e−6–1e−2; learning_rate_init 1e−4–1e−1; batch_size 32–256; activation ∈ {relu, tanh, logistic}; solver ∈ {adam, lbfgs}; beta_1 0.8–0.99; beta_2 0.9–0.999. RR (meta): alpha 0.001–1000.	RF: n_estimators = 300; max_depth = 5; min_samples_split = 7; min_samples_leaf = 1; max_features = log2; bootstrap = False. GBR: n_estimators = 500; learning_rate ≈ 0.0240; max_depth = 10; min_samples_split = 6; min_samples_leaf = 1; subsample = 1.0; max_features = 0.7. SVR: C = 0.1; epsilon ≈ 0.291; degree = 2; kernel = linear; gamma = auto. MLP: hidden_layer_sizes = 50; alpha = 0.01; learning_rate_init ≈ 0.0658; batch_size = 32; activation = logistic; solver = adam; beta_1 ≈ 0.952; beta_2 ≈ 0.942. RR: alpha ≈ 17.05.
GBR+CatBoost+LightGBM+MLP+LR	GBR (base learner): n_estimators 50–500; learning_rate 0.01–0.30; max_depth 3–12; min_samples_split 2–20; min_samples_leaf 1–10; subsample 0.6–1.0; max_features ∈ {sqrt, log2, 0.3, 0.5, 0.7}. CatBoost (base learner): iterations 50–500; learning_rate 0.01–0.30; depth 3–12; l2_leaf_reg 1–10; random_strength 0.1–10; bagging_temperature 0–1. LightGBM (base learner): n_estimators 50–500; learning_rate 0.01–0.30; max_depth 3–12; num_leaves 20–100; min_child_samples 5–50; subsample 0.6–1.0; colsample_bytree 0.6–1.0. MLP (base learner): hidden_layer_sizes 50–300; alpha 1e−6–1e−2; learning_rate_init 1e−4–1e−1; batch_size 32–256; activation ∈ {relu, tanh, logistic}; solver ∈ {adam, lbfgs}; beta_1 0.8–0.99; beta_2 0.9–0.999. LR (meta): not tuned.	GBR: n_estimators = 500; learning_rate = 0.01; max_depth = 8; min_samples_split = 2; min_samples_leaf = 1; subsample = 1.0; max_features = 0.7. CatBoost: iterations = 500; learning_rate ≈ 0.0105; depth = 3; l2_leaf_reg = 10; random_strength = 10; bagging_temperature = 1.0. LightGBM: n_estimators = 50; learning_rate ≈ 0.300; max_depth = 12; num_leaves = 100; min_child_samples = 5; subsample = 0.6; colsample_bytree = 0.6. MLP: hidden_layer_sizes = 50; alpha = 0.01; learning_rate_init = 0.0001; batch_size = 32; activation = logistic; solver = lbfgs. LR: no tuned hyperparameters.

RF+GBR+ExtraTrees+CatBoost+RR	RF (base learner): n_estimators 50–500; max_depth 5–30; min_samples_split 2–10; min_samples_leaf 1–10; max_features ∈ {sqrt, log2, 0.3, 0.5, 0.7}; bootstrap ∈ {True, False}. GBR (base learner): n_estimators 50–500; learning_rate 0.01–0.30; max_depth 3–12; min_samples_split 2–20; min_samples_leaf 1–10; subsample 0.6–1.0; max_features ∈ {sqrt, log2, 0.3, 0.5, 0.7}. ExtraTrees (base learner): n_estimators 50–500; max_depth 5–30; min_samples_split 2–20; min_samples_leaf 1–10; max_features ∈ {sqrt, log2, 0.3, 0.5, 0.7, 1.0}; bootstrap ∈ {True, False}. CatBoost (base learner): iterations 50–500; learning_rate 0.01–0.30; depth 3–12; l2_leaf_reg 1–10; random_strength 0.1–10; bagging_temperature 0–1. RR (meta): alpha 0.001–1000.	RF: n_estimators = 484; max_depth = 5; min_samples_split = 6; min_samples_leaf = 8; max_features = 0.7; bootstrap = False. GBR: n_estimators = 500; learning_rate = 0.01; max_depth = 12; min_samples_split = 18; min_samples_leaf = 1; subsample ≈ 0.955; max_features = 0.7. ExtraTrees: n_estimators = 332; max_depth = 18; min_samples_split = 20; min_samples_leaf = 7; max_features = log2; bootstrap = False. CatBoost: iterations = 289; learning_rate ≈ 0.0582; depth = 7; l2_leaf_reg ≈ 8.79; random_strength ≈ 9.03; bagging_temperature ≈ 0.621. RR: alpha = 0.001.
GBR+XGBoost+CatBoost+LightGBM+LR	GBR (base learner): n_estimators 50–500; learning_rate 0.01–0.30; max_depth 3–12; min_samples_split 2–20; min_samples_leaf 1–10; subsample 0.6–1.0; max_features ∈ {sqrt, log2, 0.3, 0.5, 0.7}. XGBoost (base learner): n_estimators 50–500; learning_rate 0.01–0.30; max_depth 3–12; min_child_weight 1–10; subsample 0.6–1.0; colsample_bytree 0.6–1.0. CatBoost (base learner): iterations 50–500; learning_rate 0.01–0.30; depth 3–12; l2_leaf_reg 1–10; random_strength 0.1–10; bagging_temperature 0–1. LightGBM (base learner): n_estimators 50–500; learning_rate 0.01–0.30; max_depth 3–12; num_leaves 20–100; min_child_samples 5–50; subsample 0.6–1.0; colsample_bytree 0.6–1.0. LR (meta): not tuned.	GBR: n_estimators = 216; learning_rate ≈ 0.0143; max_depth = 4; min_samples_split = 14; min_samples_leaf = 2; subsample ≈ 0.634; max_features = 0.5. XGBoost: n_estimators = 472; learning_rate ≈ 0.0149; max_depth = 12; min_child_weight = 1; subsample ≈ 0.941; colsample_bytree ≈ 0.925. CatBoost: iterations = 500; learning_rate ≈ 0.0804; depth = 4; l2_leaf_reg ≈ 5.23; random_strength ≈ 6.90; bagging_temperature ≈ 0.981. LightGBM: n_estimators = 301; learning_rate ≈ 0.0140; max_depth = 12; num_leaves = 100; min_child_samples = 5; subsample = 0.6; colsample_bytree ≈ 0.759. LR: no tuned hyperparameters.
